# Insights into the Preservation of the Homomorphic Sex-Determining Chromosome of *Aedes aegypti* from the Discovery of a Male-Biased Gene Tightly Linked to the M-Locus

**DOI:** 10.1093/gbe/evu002

**Published:** 2014-01-06

**Authors:** Andrew Brantley Hall, Vladimir A. Timoshevskiy, Maria V. Sharakhova, Xiaofang Jiang, Sanjay Basu, Michelle A.E. Anderson, Wanqi Hu, Igor V. Sharakhov, Zach N. Adelman, Zhijian Tu

**Affiliations:** ^1^Department of Biochemistry, Virginia Tech; ^2^Program of Genetics, Bioinformatics, and Computational Biology, Virginia Tech; ^3^Department of Entomology, Virginia Tech; ^4^Fralin Life Science Institute, Virginia Tech

**Keywords:** sex-determination, sex chromosomes, chromosome quotient, *myo-sex*, nonrecombining region, sexual antagonism

## Abstract

The preservation of a homomorphic sex-determining chromosome in some organisms without transformation into a heteromorphic sex chromosome is a long-standing enigma in evolutionary biology. A dominant sex-determining locus (or M-locus) in an undifferentiated homomorphic chromosome confers the male phenotype in the yellow fever mosquito *Aedes aegypti*. Genetic evidence suggests that the M-locus is in a nonrecombining region. However, the molecular nature of the M-locus has not been characterized. Using a recently developed approach based on Illumina sequencing of male and female genomic DNA, we identified a novel gene, *myo-sex*, that is present almost exclusively in the male genome but can sporadically be found in the female genome due to recombination. For simplicity, we define sequences that are primarily found in the male genome as male-biased. Fluorescence in situ hybridization (FISH) on *A. aegypti* chromosomes demonstrated that the *myo-sex* probe localized to region 1q21, the established location of the M-locus. *Myo-sex* is a duplicated myosin heavy chain gene that is highly expressed in the pupa and adult male. *Myo-sex* shares 83% nucleotide identity and 97% amino acid identity with its closest autosomal paralog, consistent with ancient duplication followed by strong purifying selection. Compared with males, *myo-sex* is expressed at very low levels in the females that acquired it, indicating that *myo-sex* may be sexually antagonistic. This study establishes a framework to discover male-biased sequences within a homomorphic sex-determining chromosome and offers new insights into the evolutionary forces that have impeded the expansion of the nonrecombining M-locus in *A. aegypti*.

## Introduction

In *Aedes* and *Culex* mosquitoes, male development is initiated by a dominant male-determining locus (M-locus) located on a homomorphic sex-determining chromosome (McClelland 1962; Newton et al. 1974). On the other hand, *Anopheles* mosquitoes have fully differentiated heteromorphic sex chromosomes where the male-determining locus resides on the nonrecombining Y chromosome (Clements 1992; Marın and Baker 1998). Evolutionary models suggest that homomorphic sex-determining chromosomes may eventually progress into heteromorphic sex chromosomes (Charlesworth et al. 2005). After the acquisition of a sex-determining locus, linkage between sexually antagonistic genes and the sex-determining-locus is favored and may lead to an initial suppression of recombination followed by progressive expansion of the nonrecombining region, transforming a homomorphic sex-determining chromosome into a heteromorphic sex chromosome (Bull 1983; Charlesworth B and Charlesworth D 2000; Charlesworth et al. 2005; Bachtrog 2006; Charlesworth and Mank 2010; Bachtrog 2013). However, this fate is not inevitable because examples of old homomorphic sex-chromosomes have been observed (Charlesworth and Mank 2010).

*Aedes aegypti* has three pairs of chromosomes, with chromosome 1 the shortest, chromosome 2 the longest, and chromosome 3 of medium length (Rai 1963; Sharakhova et al. 2011; Timoshevskiy et al. 2013). The M-locus has been mapped to chromosome 1, band 1q21 (McClelland 1962). For simplicity, we follow the nomenclature proposed by Motara and Rai (1978) and refer to the copy of chromosome 1 with the M-locus, the M-chromosome, and the copy without the M-locus, the m-chromosome. Genetic evidence suggests that there is a nonrecombining M-locus in *A. aegypti* (Severson et al. 2002; Toups and Hahn 2010). There are also cytological differences between the M-locus and the m-locus, consistent with clear differentiation between the loci (Motara and Rai 1977; Motara and Rai 1978). The M-locus can be found in noncanonical locations in other *Culicinae* mosquitoes (Ferdig et al. 1998; Venkatesan et al. 2009), indicating either translocation or turnover of the sex-determining gene. However, the M-locus of *A. aegypti* and *Culex pipiens* are linked to the same markers, suggesting that the *A. aegypti* M-locus likely shares a common origin with *C. pipiens* (Malcolm et al. 1998; Mori et al. 1999). Thus, the sex-determining chromosome of the yellow fever and dengue fever mosquito *A. aegypti* may have remained stubbornly homomorphic because of the evolutionary divergence between the *Aedes* and *Culex* lineages. It is suggested that the common ancestor of the mosquito family had a homomorphic sex-determining chromosome (Toups and Hahn 2010).

Despite the publication of the *A. aegypti* genome, sequences from the M-locus remain uncharacterized (Nene et al. 2007). In species with well-defined heteromorphic sex chromosomes such as *Drosophila* and *Anopheles*, Y chromosome sequences have proven resistant to traditional methods of sequence assembly because of the repetitive nature of the Y (Carvalho 2002; Krzywinski et al. 2004, 2006). In fact, Y chromosome sequences are rarely annotated in published genomes of nonmodel organisms (Carvalho et al. 2009). Recent advances in sequencing technologies have allowed for the identification of more Y chromosome sequences. We identified a single Y chromosome gene in *Anopheles* mosquitoes by comparing male Illumina data to female Illumina data (Criscione et al. 2013). We also identified six more Y chromosome genes in *Anopheles* mosquitoes using a more efficient approach called the chromosome quotient (abbreviated CQ) (Hall et al. 2013).

In this study, we applied the CQ method to *A. aegypti*, a species with a homomorphic sex-determining chromosome, and identified sequences that were primarily present in the male genome but were sporadically present in the female genome due to recombination. In this study we call sequences primarily present in the male genome male-biased sequences, without implying patterns of gene expression. The male-biased sequences discovered by the CQ method include: a novel gene, *myo-sex*, and the full-length bacterial artificial chromosome (BAC) clone NDL62N22. Based on homology, strong purifying selection, and differential expression between males and recombinant females, *myo-sex* appears to be a functionally important myosin heavy-chain gene with the possibility for sexually antagonistic effects. *Myo-sex* is a product of an ancient duplication event, and it is tightly linked to the M-locus. We discuss alternative scenarios that may begin to explain why *myo-sex* has not been incorporated into the nonrecombining M-locus and shed light on the maintenance of homomorphic M chromosome in *A. aegypti*.

## Materials and Methods

### CQ Analysis

The CQ method was originally devised as an approach to identify Y chromosome sequences (Hall et al. 2013), and it is adapted here to identify male-specific or male-biased sequences in *A. aegypti.* The CQ method is based on the principle that male-specific or male-biased sequences should be present in male sequence data and absent from female sequence data. This simple principle is complicated by the presence of nearly identical autosomal paralogs and repetitive sequences. Thus, instead of searching for sequences exclusive to the male sequence data, female alignments are permitted as long as there are at least five times more alignments from the male (CQ ≤ 0.2). The selection of this CQ cutoff is described below and at the beginning of the Results section. Interference from repetitive sequences is reduced by using extremely strict alignment parameters. For an alignment to be valid, it must align with 100% nucleotide over the entire extent of the Illumina (Illumina, San Diego, CA) read. Bowtie, the ultrafast short-read aligner, is used for alignment (Langmead et al. 2009). To reduce the rate of false positives, a threshold for the number of male alignments is used. For this study, the threshold was set at 30.

Male and female Illumina sequence data were generated to perform the CQ method in *A. aegypti*. Genomic DNA was isolated separately from ten male and six virgin female Liverpool strain *A. aegypti*. The Qiagen (Hilden, Germany) DNeasy Blood and Tissue kit was used to isolate the DNA following the manufacturer-suggested protocols. The male and female genomic DNA samples were each subjected to two lanes of Illumina sequencing on a HiSeq 1000 producing 99-bp paired-end reads. The coverage of the resulting reads was approximately 63× for males and 65× for females. The male and female sequence data were deposited to the National Center for Biotechnology Information Sequence Read Archive (NCBI SRA) (SRP023515).

A control for the CQ method was performed on known *A. aegypti* autosomal sequences for normalization and to quantify the rate of false positives. Known autosomal supercontigs (Nene et al. 2007) were retrieved and repetitive sequences annotated by RepeatMasker, and gaps represented by *N*s were removed generating 7,713 sequences. CQs were calculated for the 7,713 sequences with the male and female Illumina sequence data. These autosomal sequences had a median CQ of 1.274, slightly higher than the expected CQ of 1, indicating there was more female sequence data than male sequence data. Subsequently, we normalized all CQs by 1.274.

We then calculated the CQs for the *A. aegypti* IB12 strain supercontigs (version AaegL1), contigs (version AaegL1), transcripts (version AaegL1.3), expressed sequence tag (ESTs) (*Aedes-aegypti*_EST-CLIPPED_2012-12.fa.gz), and BAC-ends (*Aedes-aegypti*-Liverpool_BAC-ENDS_2012-12.fa.gz). These sequences were all downloaded from VectorBase (Vectorbase.org). We noticed that there was a relatively high rate of apparent false-positive sequences, so we set CQ 0.2 as the cutoff for further analysis of male-specific or male-biased candidates.

### Further Bioinformatics Analysis of Candidate Male-Biased Sequences

Two additional steps were implemented to identify male-specific or male-biased genes among the sequences mentioned above with CQs less than 0.2. First, these sequences were compared with RNA-seq data spanning developmental time points from embryo to adult, including separate adult male and female sequence data (SRA SRP009679) (Biedler et al. 2012). The transcriptome sequence data was compared with all the sequences with BlastN requiring 100% nucleotide identity with an e-value less than 1 × 10^−^^5^. Second, to further ensure male-specificity or male-bias, candidate sequences were compared with the male and female Illumina sequence data, using BlastN (e-value cutoff set at 1 × 10^−^^5^). This step employs relaxed search parameters and thus allows us to filter sequences that only have slight variations between males and females.

### Obtaining the Sequence of the Full-Length *Myo-sex* Transcript

Reverse transcription PCR (RT-PCR) was used to connect the five myosin ESTs. Phire II DNA polymerase (Thermo Scientific, Pittsburgh, PA) was used for RT-PCR following the manufacturer-suggested protocol. cDNA was made from Liverpool *A. aegypti* pupa. RNA was isolated using the mirVana RNA isolation kit (Life Technologies, Carlsbad, CA) following the manufacturer protocol for total RNA isolation. cDNA was then synthesized with SuperScript RT (Life Technologies). The PCR products were run on 1% agarose gels and gel-purified with the GE gel purification kit (GE Healthcare, United Kingdom) following the manufacturer-suggested protocol. The RT-PCR products were cloned into the pGEM-T Easy vector (Promega, Madison, WI) or CloneJET vector (Thermo Scientific) and sequenced. The 5′ and 3′ rapid amplification of cDNA ends (RACE) was performed to obtain the terminal ends of the *myo-sex* transcript. The SMARTer RACE cDNA Amplification kit (Clonetech Laboratories, Mountain View, CA) was used to perform RACE following the manufacturer-suggested protocol. RACE-ready cDNA was synthesized from pupa cDNA. The RACE PCR products were cloned into the pGEM-T EASY vector and sequenced. Vector sequences were removed from the sequence results using NCBI VecScreen. Assembly was performed with Cap3 (Huang and Madan 1999). The sequence was corrected with the consensus of pupa RNA-seq using CLC Genomics Workbench (www.clcbio.com, last accessed January 14, 2014). The assembled *myo-sex* transcript was submitted to GenBank (KF150020) and is available in the supplementary materials (supplementary file S1, Supplementary Material online). The primers used for RT-PCR and RACE are available in the supplementary materials (supplementary table S1, Supplementary Material online).

### Generating Transgenic Lines with Transgenes Flanking the M-Locus

The methods for the generation of the sensor transgenic strain are detailed in Adelman et al. (2008). To generate transgenic line J2, *A. aegypti* Liverpool strain embryos (*n* = 663) were injected with 300 ng/µl pGL3-PUb-Mos1 and 500 ng/µl PUb-DsRED construct as previously described (Carpenetti et al. 2012). G_0_ injection survivors with DsRED somatic transient expression (10.7%) were crossed to Liverpool strain of the opposite gender, resulting in the establishment of five pools designated J1–J5 (two female, three male). Eight positive individuals out of 400 total screened were identified from line J2. Mosquitoes for this study were reared using techniques and conditions described in Aryan et al. (2013). Before crossing with the 3xP3-sensor strain, the J2 transgene insertion was moved into the *kh^w^* genetic background.

### Fluorescence In Situ Hybridization

Fluorescence in situ hybridization (FISH) was performed on mitotic and polytene chromosomes using the methods described in Timoshevskiy et al. (2012). Polytene chromosomes were prepared using salivary glands of the fourth instar larvae and mitotic chromosomes were prepared using imaginal discs from the fourth instar larvae. Slides were placed in 2× saline-sodium citrate buffer (SSC) for 30 min at 37 °C, pretreated with 0.1 mg/ml of pepsin for 5 min at 37 °C, denatured in 70% formamide in 2× SSC at 72 °C for 2 min, and dehydrated in a series of cold (−20 °C) ethanol (70%, 80%, 100%) for 3–5 min each. Hybridization mix contained: 50% formamide, 10% dextran sulfate, 100 ng of each probe per slide, and 3 µg of unlabeled repetitive DNA fractions per probe. DNA/probe mix was precipitated by adding one-tenth volume of sodium acetate and 2 vols. of 100% ethanol. The DNA pellet was dissolved in “master mix” (10 µl per slide) that contained 50% formamide, 10% dextran sulfate, and 1.2× SSC. After that, DNA was denatured at 96 °C for 7 min. Denatured DNA was placed on ice for 1 min and incubated at 37 °C for 30 min for prehybridization with unlabeled repetitive DNA fractions (C_0_t3 DNA). Repetitive DNA fractions were isolated from *A. aegypti* genomic DNA. DNA was denatured and allowed to reassociate at 60 °C in 1.2× SSC for 15–150 min depending on concentration. Single-stranded DNA was digested using S_1_ nuclease (Invitrogen Corporation, Carlsbad, CA). Ten microliters of hybridization mix was placed on a slide, which had been preheated to 37 °C, under a 22 × 22 mm coverslip, and glued by rubber cement. Slides were hybridized at 37 °C in a dark humid chamber overnight. After hybridization, slides were dipped for washing in a Coplin jar with 0.4×SSC, 0.3% Nanodept-40 at 72 °C for 2 min, and then in 2× SSC, 0.1% Nanodept-40 at room temperature for 5 min. Thereafter, slides were counterstained using Prolong with 4′,6-diamidino-2-phenylindole (DAPI) (Invitrogen Corporation) or incubated with 1-µm YOYO-1 solution in 1× PBS for 10 min in the dark, rinsed in 1× phosphate buffered saline (PBS), and then enclosed in antifade Prolong Gold (Invitrogen Corporation) under a coverslip. Slides were analyzed using a Zeiss LSM 510 Laser Scanning Microscope (Carl Zeiss Microimaging, Thornwood, NY) at 1,000× magnification.

### *Myo-sex* Expression Analysis

The RNA used for the expression profile of *myo-sex* was isolated using the mirVana RNA isolation kit (Life Technologies) following the manufacturer protocols for total RNA isolation. cDNA was then synthesized with the SuperScript III RT kit (Life Technologies) following the manufacturer-suggested protocols. Using cDNA spanning developmental time points, RT-PCR was performed on *myo-sex* using Phire II DNA polymerase (Thermo Scientific). The PCR products were verified to be *myo-sex* by sequencing. For the expression profile, 27 cycles were used with a melting temperature of 63 °C. To further assess *myo-sex* expression in Liverpool adult females, cDNA samples from virgin females and blood-fed females were amplified for 32 cycles (supplementary fig. S1, Supplementary Material online). To assess the expression of *myo-sex* in recombinant females that acquired *myo-sex*, both RT-PCR and quantitative RT-PCR (qRT-PCR) were performed. qRT-PCR were performed using the SYBR Green-based GoTaq qPCR kit from Promega (Madison, WI) on a ABI 7300 real-time PCR system (Life Technologies). Three biological replicates were included and a ribosomal protein gene *RPS7* was used to normalize expression. Relative expression levels were quantified using ΔCt relative quantification method with *RPS7* as the endogenous control (Sengul and Tu 2010). All primers are available in supplementary table S1, Supplementary Material online.

### *Myo-sex* Evolutionary Analysis

The d*N*/d*S* ratio of *myo-sex* was calculated using JCoDA using a sliding window size of 200, and a jump size of 20. The Yang and Neilson d*N*/d*S* substitution model was used. The *myo-sex* phylogeny was generated with MrBayes (Huelsenbeck and Ronquist 2001) using MUSCLE (Edgar 2004) for the alignments. The alignments and parameters used for phylogenetic inference are provided in supplementary file S2, Supplementary Material online. For the synteny figure, orthologs were assigned by OrthoDB (Waterhouse et al. 2011) and relative positions assigned by VectorBase.

### An Extremely Male-Biased BAC Clone

CQ analysis of BAC-ends identified several BACs as male-specific or highly male-biased candidates, and one such BAC was sequenced after PCR verification with male and female genomic DNA as templates. DNA was isolated from BAC NDL62N22 and was sequenced with PacBio sequencing. A single SMRT cell of PacBio sequencing (Pacific Biosciences, CA) was performed on the *A. aegypti* BAC clone NDL62N22 along with five other BACs from different species for a different project. The resulting PacBio sequences were assembled by Russell Durrett at the Weill Cornell Medical College using the Hierarchical Genome Assembly Process (HGAP) from PacBio. The resulting contig contained the BAC cloning vector, which was subsequently removed. The resulting BAC was 94,552-bp long. The sequence of BAC NDL62N22 is provided in the supplementary materials (supplementary file S1, Supplementary Material online).

### Male-Specific Amplification of *Myo-sex* and BAC Clone NDL62N22

DNA was isolated from adult mosquitoes using the DNAzol (Life Technologies) reagent following the manufacturer protocols. Genomic DNA samples were isolated from pools of five individuals. PCR was performed in 25 males and 25 females from both the Liverpool and *kh^w^* strains using 30 cycles with a melting temperature of 63 °C. The PCR products were verified to be the expected product by sequencing. Phire II DNA polymerase (Thermo Scientific) was used for the PCR following the manufacturer-specified protocol. All primer sequences used in this study are available in supplementary table S1, Supplementary Material online.

## Results

### Identification of Candidate Male-Biased Genomic Sequences in *A. aegypti*

**Fig. 1.—:**
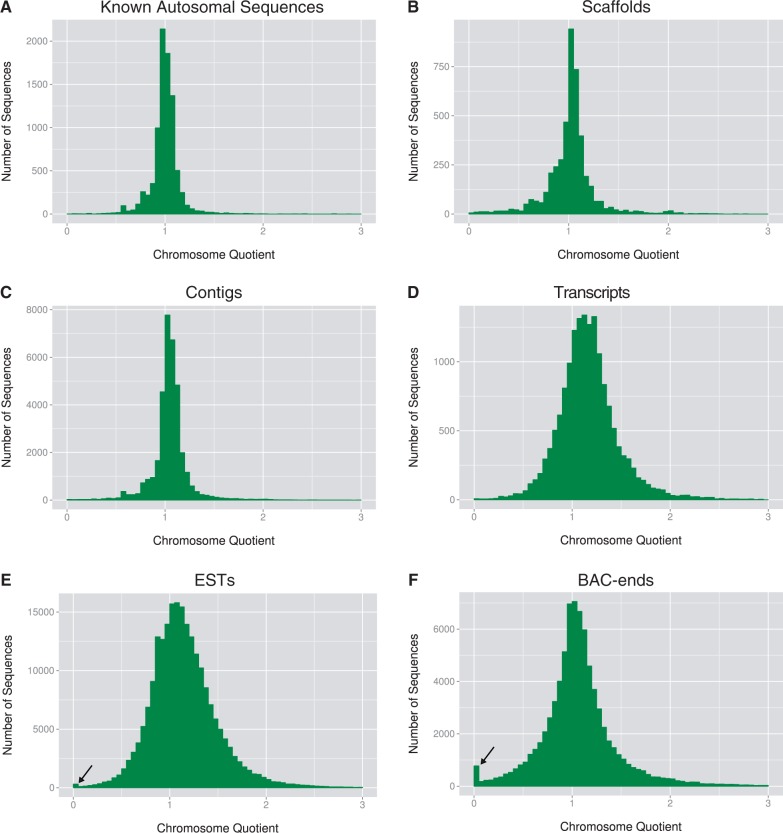
The distribution of CQs from *A. aegypti* including: (*A*) known autosomal sequences, (*B*) all scaffolds, (*C*) all contigs, (*D*) all transcripts, (*E*) ESTs, and (*F*) BAC-ends. Arrows in (*E*) and (*F*) indicate peaks in the distribution of CQs near zero, the CQ expected for male-biased sequences.

Separate male and female Illumina sequence data were generated from adults of the Liverpool strain of *A. aegypti,* resulting in coverage of 65× and 63×, respectively (SRA: SRP023515). To select a cutoff CQ for screening candidate male-specific sequences, we calculated CQs for supercontigs with known autosomal positions (Nene et al. 2007). Each known autosomal supercontig was split at gaps denoted by *N*s and bases masked by repeat masker. Chromosome quotients were calculated for 7,713 of the resulting autosomal sequences (fig. 1). Only seven of the split supercontig sequences had CQs less than 0.2, and only one had a CQ of zero. Thus, 0.2 was chosen as the cutoff for further analysis of male-specific or male-biased candidates.

We searched for sequences with alignments only from the male sequence data using various reference data sets from *A. aegypti* including: the *A. aegypti* IB12 strain supercontigs, contigs, annotated transcripts, Rockefeller strain ESTs, and Liverpool strain BAC-ends (fig. 1) (Krebs et al. 2002; Jiménez et al. 2004). As expected, the overwhelming majority of sequences had CQs distributed around one, the CQ expected for sequences present in equal numbers in males and females. Each set of reference sequences had sequences with alignments only from the male sequence data or many more male alignments than female alignments (table 1). In the current study, our focus was the identification of male-specific or male-biased genes, not slight sequence variations that may exist between the male and female samples nor highly repetitive sequences. Thus, putative genes were identified by comparing the sequences with CQs less than 0.2 to the NCBI nonredundant protein database and transcriptome sequence data spanning developmental time points from embryo to adult, including separate adult males and females (SRA SRP009679; Biedler et al. 2012). No genes were identified in the supercontigs or contigs with CQs less than 0.2. We identified 28 transcripts with CQs less than 0.2. However, further analysis showed that none of the transcripts had characteristics of male-biased genes. Twenty-two either showed no evidence of transcription or had transcription in females according to our transcriptome analysis. The six remaining transcripts derive from contigs that do not appear to be male-biased based on CQ analysis. None of the 28 transcripts were male-biased when relaxed BlastN parameters were used for comparison to the male and female Illumina data. Due to our lack of success finding male-biased genes in the published genome sequences, we focused on the unassembled ESTs and BACs: the ESTs because they were automatically candidates for genes, and the BACs because sequencing full-length male-biased BAC clones could result in the identification of genes.

**Table 1:** The Total Number of Sequences and the Number of Sequences with CQs less than 0.2 from the *A. aegypti* Scaffolds, Contigs, Transcripts, ESTs, and BAC-Ends

Sequences	Total Sequences	CQs < 0.2
Known autosomal sequences	7,713	7
Supercontigs	4,505	40
Contigs	35,292	106
Transcripts	16,106	28
ESTs	221,753	747
BAC-ends	79,413	1,423

Note.—In Table 1, total sequences is the number of sequences for which CQs were calculated, with greater than or equal to 30 alignments from the male sequence data.

Candidate male-biased EST and BAC sequences were further narrowed down by using relaxed alignment parameters to map the male and female Illumina data to the potential male-biased sequences with BlastN. Even with the relaxed alignment parameters, 15 of the ESTs and 17 BAC-ends appeared unique or highly biased to the male sequence data. Analysis of the BAC sequences is described in a later section. Eight of the ESTs were removed because they had alignments from the adult female transcriptome data. The seven remaining ESTs were compared with the genome with BlastN. One of the seven aligned with 97% nucleotide identity to a long contig that is not male biased. BlastX was used to compare the remaining six ESTs to the NCBI nonredundant protein database. One EST was eliminated as bacterial contamination. The remaining five ESTs aligned to myosin heavy chain genes, with e-values less than 1 × 10^−^^40^ and had CQs of zero (table 2).

**Table 2:** The CQs and the Ratio of Alignments Based on Relaxed BlastN Parameters to the Five Male-Biased Myosin ESTs and the Assembled *Myo-sex* Transcript (KF150020)

Sequence	Female Alignments Bowtie	Male Alignments Bowtie	CQ	Female Alignments BlastN	Male Alignments BlastN	Ratio of Alignments
BQ789600.1	0	34	0	0	125	0
BQ789612.1	0	63	0	5	175	0.028
BQ789634.1	0	74	0	36	147	0.245
BQ789633.1	0	79	0	5	177	0.028
DV248113.1	0	91	0	0	149	0
*Myo-sex* transcript	0	899	0	180	1,820	0.098

Note.—Sequences starting with BQ and DV are ESTs from VectorBase.

### Discovery of *Myo-sex*, an Extremely Male-Biased Myosin Heavy-Chain Gene

When compared with the *A. aegypti* transcripts with BlastN and BlastX, the five remaining ESTs all aligned to the *A. aegypti* gene *AAEL005656* with approximately 83% nucleotide identity and 97% amino acid identity. RT-PCR and subsequent sequencing confirmed that all five ESTs derived from the same novel male-biased gene. Primers were also designed for 5′ and 3′ RACE to identify the terminal portions of the transcript. The full-length 5,990 bp myosin-gene transcript was assembled using the sequencing results of the RT-PCR and RACE products. We call this gene “*myo-sex*” because it is homologous to a myosin heavy chain gene and is male-biased. *Myo-sex* is not represented in the current *A. aegypti* genome assembly. However, a BlastN search of the *A. aegypti* trace database used for genome assembly found 20 alignments with greater than 98% identity, indicating that its absence from the current assembly is likely a reflection of poor assembly or low coverage near the M-locus. FISH experiments described below are consistent with our interpretation.

**Fig. 2.—:**
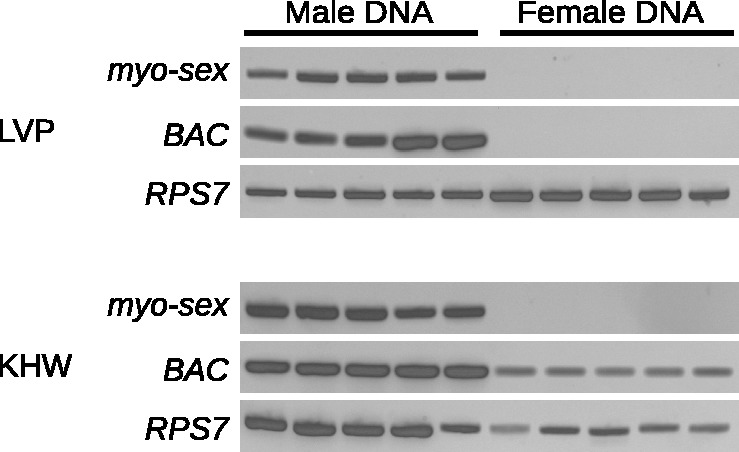
Genomic DNA amplification of *myo-sex* and BAC NDL62N22 from five pools of five male and female mosquitoes from the (*A*) Liverpool and (*B*) *kh^w^* strains of *A. aegypti*. A ribosomal protein gene (*RPS7*) was amplified in both male and female samples to verify the integrity of the genomic DNA.

To verify that *myo-sex* is a male-specific gene, PCR was performed on five pools of male (*n* = 5 per pool) and five pools of female (*n* = 5 per pool) genomic DNA from both the Liverpool and *kh^w^* strains of *A. aegypti*. Primers for *myo-sex* amplified a PCR product in male-genomic DNA from both the Liverpool and *kh^w^* strains of *A. aegypti* but not from female genomic DNA from either strain (fig. 2). Amplicons were cloned and sequenced, and verified to be *myo-sex*. A ribosomal protein gene (*RPS7)* was amplified in both males and females to validate the integrity of the genomic DNA. The CQ of the assembled *myo-sex* transcript is zero, and even with relaxed BlastN parameters, few female reads align (table 2), further asserting that *myo-sex* is male-specific in our sequencing data.

### *Myo-sex* Hybridizes to the M-Locus and Can Undergo Recombination with the M-Chromosome

**Fig. 3.—:**
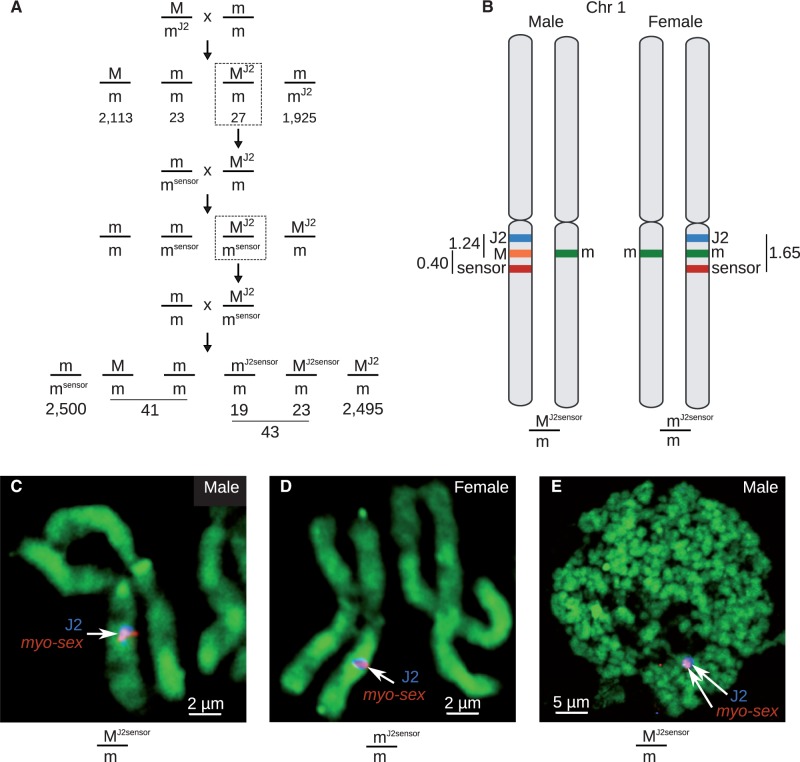
*Myo-sex* hybridizes to the location of the *A. aegypti* M-locus and can still recombine with the m-chromosome. (*A*) Using two transgenic strains of *A. aegypti* with sex-linked insertions, sensor and J2, a transgenic strain of *A. aegypti* was generated that has transgenes flanking the M-locus on the M-chromosome, and a separate transgenic strain was generated with the two transgenes corresponding positions on the m-chromosome. Note that one of the 43 transgenic recombinants died before we can determine its sex. (*B*) A representation of the relative position of the transgenes with respect to the M-locus and the m-locus. Numbers represent linkage in centimorgans. FISH on mitotic chromosomes from the *kh^w^* strain of *A. aegypti* in males (*C*) and females (*D*) with probes for J2 and *myo-sex*. In (*C*), the blue J2 signal spans the entire presumed M-locus and the surrounding region. (*E*) FISH on male polytene chromosome shows that the *myo-sex* signal is fully within the J2 signal, indicating that *myo-sex* is located between the J2 and sensor transgenes. *Myo-sex* was present in the transgenic strain with the transgenes on the m-chromosome. When J2 recombined with the m-chromosome to generate this transgenic line, *myo-sex* also moved to the m-chromosome with J2 indicating that *myo-sex* can still recombine.

To further verify that *myo-sex* was truly male-specific, we sought to determine its physical location within the *A. aegypti* genome. We took advantage of several transgenic strains in our possession. Adelman et al. (2008) described a transposon insertion strain (referred to here as “sensor”) marked with a green fluorescent protein that resides 0.40 cM from the sex-locus on the m-chromosome. The 0.40 cM recombination distance is an average of three replicates that range from 0.12 to 0.64, and the variability may be related to the location of the sensor transgene in the rDNA repeats. During the course of other experiments, we obtained a second transgenic strain (J2, marked with DsRED) that appeared to be sex-linked as well. The J2 insertion recombined from the m-chromosome to the M-chromosome at a frequency of approximately 1.24% (fig. 3*A*). Subsequent crossing with sensor mosquitoes allowed us to establish both M-chromosome and m-chromosome strains containing both transgenes (fig. 3*A*). Calculated recombination distances between sex/sensor, sex/J2, and sensor/J2 established that these two transgenes flank the M-locus (fig. 3*B*). There are fewer J2-M and more M-sensor recombinants in the F3 than expected according to their respective average recombination frequencies, which may in part result from the previously observed variability in recombination distance (Adelman et al. 2008). FISH was performed on mitotic chromosomes of both M^J2sensor^/m and m/m^J2sensor^ individuals (fig. 3*C* and *D*). In both cases, *myo-sex* and the J2/sensor transgene colocalized to only one copy of the q-arm of chromosome 1, band 1q21, the established location of the sex-determining locus. The fact that *myo-sex* hybridized to a single m^J2sensor^ chromosome suggests that this gene recombined alongside the J2 transgene during the creation of the double-marked strain. In contrast, if *myo-sex* was truly autosomal, the *myo-sex* probe should have hybridized to both copies of chromosome 1. Hybridization to polytene chromosomes confirmed the colocalization of the sex-linked transgenes and *myo-sex* (fig. 3*E*). We conclude that *myo-sex* is a novel *A. aegypti* gene that is proximal to the M-locus and therefore present primarily in the male genome but can sporadically appear in the female genome due to recombination.

### *Myo-sex* Expression Profile, Evolutionary Origin, and Selective Pressure

**Fig. 4.—:**
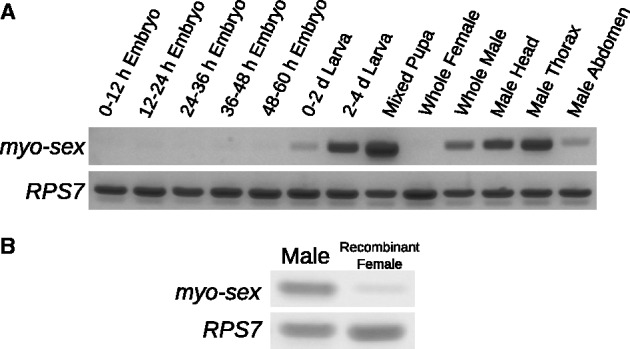
*Myo-sex* expression. (*A*) The expression profile of *myo-sex* from RT-PCR on cDNA spanning developmental time points. PCR products indicate expression of *myo-sex* in the larva, pupa, and adult male. A ribosomal protein gene (*RPS7*) was amplified in both male and females samples to verify the integrity of the cDNA. (*B*) RT-PCR on males and recombinant females. cDNA were synthesized from ten pooled individuals in each sample. The recombinant females were the m/m^J2sensor^ females shown in figure 3*A*. Thirty-two cycles of amplification were performed.

The expression profile of *myo-sex* was generated with cDNA spanning developmental time points (fig. 4*A*). RT-PCR was performed using total RNA isolated from: 1–12-h embryos, 12–24-h embryos, 24–36-h embryos, 36–48-h embryos, 48–60-h embryos, 0–2-day larva, 2–4-day larva, 0–2-day pupa, adult females, adult males, male heads, male thorax, and male abdomens. The highest level of expression from RT-PCR appears in the pupa, with dark bands also appearing in the adult male, and male thorax. No bands were observed in the female samples where *myo-sex* was absent (fig. 4*A* and supplementary fig. S1, Supplementary Material online). The general expression profile shown in figure 4*A* was consistent with RNA-seq analysis (supplementary table S2, Supplementary Material online). As expected, genomic DNA PCR indicated that *myo-sex* is present in all five of the recombinant m/m^J2sensor^ females tested (data not shown). In the recombinant females, however, RT-PCR showed only a very faint *myo-sex* band (fig. 4*B*). Similarly, qRT-PCR analysis indicated that *myo-sex* transcript level in the recombinant females that acquired *myo-sex* was equivalent to the background level observed in females without the *myo-sex* gene, both of which were hundreds of fold lower than that of the males (supplementary fig. S1, Supplementary Material online).

**Fig. 5.—:**
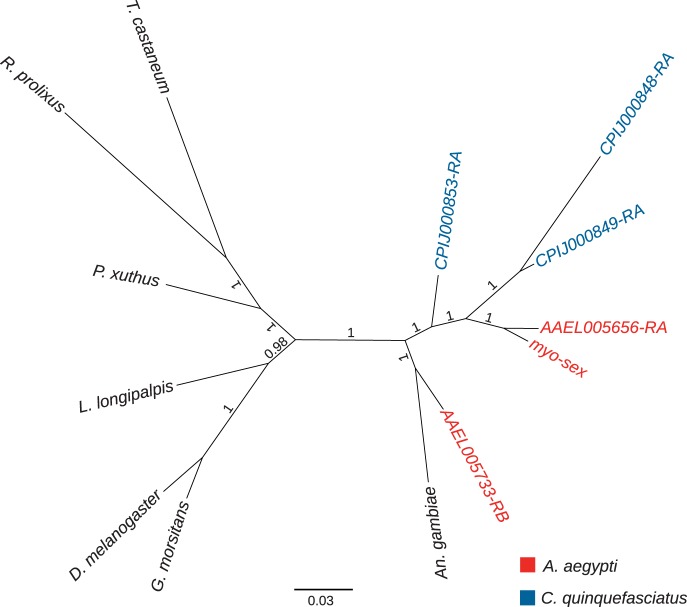
The phylogeny of *myo-sex* and other myosin heavy genes in insects. The phylogeny suggests that both *myo-sex* and *AAEL005656* originated after the evolutionary divergence of *Aedes* and *Culex* mosquitoes. The protein IDs for the genes represented in the phylogeny are as follows: *A. aegypti* (*AAEL005656-PA*), *A. aegypti* (*AAEL005733-PB*), *C. quinquefasciatus* (*CPIJ000848-RA*), *C. quinquefasciatus* (*CPIJ000849-PA*), *C. quinquefasciatus* (*CPIJ000853-PA*), *Anopheles gambiae* (*AGAP010147-PA*), *Rhodnius prolixus* (*RPRC012274-PA*), *Tribolium castaneum* (*XP_001814139.1*), *Glossina morsitans* (*GMOY005703-PA*), *Drosophila melanogaster* (*FBpp0080463*), *Lutzomyia longipalpis* (*LLOTMP009501-PA*), and *Papilo xuthus* (*BAG30740.1*). We note that the current *CPIJ000853* protein model in Vectorbase lacks approximately 350 residues at the N-terminus and contains a 200 residue insertion compared with other proteins in the alignment (supplementary file S2, Supplementary Material online). A semimanual reannotation of the *CPIJ000853* genomic sequence recovered a protein sequence that is 94% identical to *AAEL005733* (supplementary file S1, Supplementary Material online). The reannotated *CPIJ000853* grouped together with *AAEL005733* with credibility value of 1 in the new phylogeny (not shown).

**Fig. 6.—:**
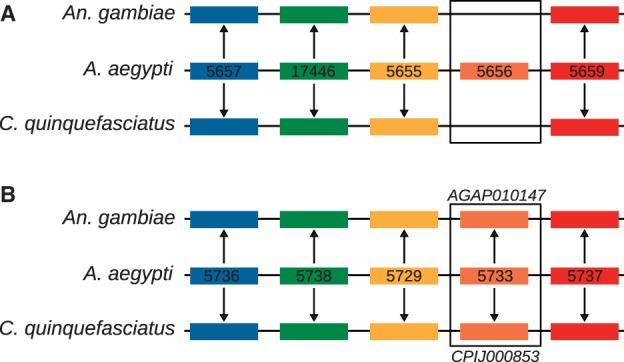
The synteny of the paralogs of *myo-sex*. The synteny of the closest paralog of *myo-sex*, *AAEL005656* (*A*) suggests that *AAEL005656* is inserted into a synteny block conserved among all three mosquitoes. The synteny of the then next closest paralog of myo-sex, *AAEL005733* (*B*), is conserved in *A. aegypti, An. Gambiae,* and *C. quinquefasciatus*. *A. aegypti* gene names preceded by the VectorBase convention AAEL00. Genes of the same color within each panel are orthologs assigned by OrthoDB. The gene names of the orthologs of *AAEL005733* are shown in (*B*).

Phylogenetic analysis suggests that *myo-sex* is a close paralog of *AAEL005656* (fig. 5). We also examined the gene synteny of its paralogs: *AAEL005656* and the slightly more distant *AAEL005733*. Based on the assignment of scaffolds to chromosomes in the *A. aegypti* genome, *AAEL005733* is located on chromosome 2, while the position of *AAEL005656* is unknown (Nene et al. 2007). *AAEL005656* appears to be an insertion that occurred after the divergence of *Aedes* and *Culex* mosquitoes. The gene synteny around *AAEL005733* is conserved in *A. aegypti, Anopheles gambiae* and *C. quinquefasciatus* (fig. 6). These results suggest that *AAEL005733* represents the ancestral gene, and that a duplication of *AAEL005733* produced *AAEL005656*/*myo-sex.* Although it is not clear whether *AAEL005656* or *myo-sex* came first, it is likely that the duplication happened after the divergence between *Aedes* and *Culex* (figs. 5 and 6).

*Myo-sex* and *AAEL005656* align well along their entire open reading frames and have a nucleotide identity of 83%. The predicted amino acid sequences of *AAEL005656* and *myo-sex* have an amino-acid identity of 97%. Using the coding sequences of *AAEL005656* and *myo-sex,* we calculated the ratio of nonsynonymous to synonymous mutations (d*N*/d*S*). The d*N*/d*S* ratio of *myo-sex* to *AAEL005656* was 0.0107, indicating strong purifying selection.

### The Sequence of BAC-Clone NDL62N22 Is Male-Biased in the Liverpool Strain But Not the *kh^w^* Strain of *A. aegypti*

**Fig. 7.—:**
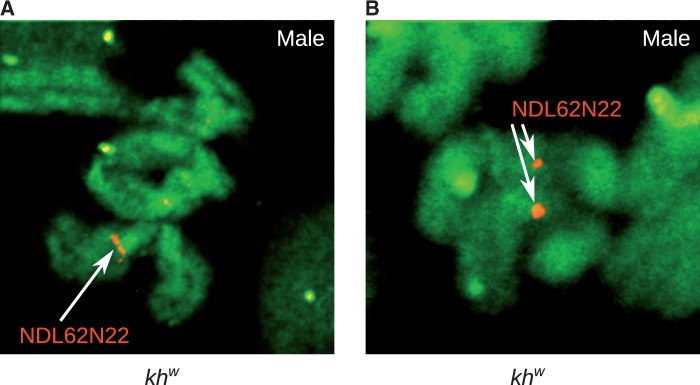
FISH on mitotic chromosomes with probes for BAC NDL62N22 in males hybridize to both the M-chromosome and the m-chromosome, indicating that the BAC is not male-biased in the *kh^w^* strain of *A. aegypti*. The signal is observed at band 1q21, the established location of the sex-determining locus.

Nine of the 17 BACs that had male-specific or highly male-biased BAC-end sequences were from a library that is no longer available. Four of the remaining eight had BAC-end sequences that were nearly identical to multiple other sequences in the genome, which makes specific PCR for these BAC-ends very difficult. We tested all four BACs for which we could design specific PCR primers. One primer set designed to amplify the BAC clone NDL62N22 (CC867386.1) amplified a male-specific PCR product from Liverpool genomic DNA (fig. 2), while the other three BAC-ends did not produce male-specific PCR products. Genomic DNA was isolated from BAC NDL62N22 and PacBio sequencing was performed. The PacBio sequencing reads were assembled using the PacBio HGAP assembler resulting in a single 94,552-bp contig. The T7 and SP6 BAC-ends align to the beginning and end of the contig, respectively. No genes were found in the 94,500-bp contig. Although primers for the BAC NDL62N22 amplified a male-specific PCR product in the Liverpool strain of *A. aegypti,* the same primers amplified a PCR product in both males and females in the *kh^w^* strain of *A. aegypti* (fig. 2). To further verify that BAC NDL62N22 is male-biased in the Liverpool strain, the full-length BAC sequence was cut into 1,000-bp fragments, and CQs were calculated for each of these fragments. The CQs for the 1,000-bp pieces clearly show male-biased segments throughout the length of the BAC with Liverpool sequence data (supplementary fig. S2, Supplementary Material online). Using FISH on mitotic chromosomes of transgenic *kh^w^* mosquitoes, the BAC hybridized to 1q21, the established location of the sex-determining locus (fig. 7). The BAC NDL62N22 hybridized to both the M-locus and the m-locus, confirming that it is not male-biased in *kh^w^* strain of *A. aegypti*, consistent with the PCR results (fig. 2*B*).

## Discussion

### Discovery of the *Myo-sex* Gene and Implications to Finding the M-Factor in a Homomorphic Sex-Determining Chromosomes

We identified extremely male-biased sequences around the *A. aegypti* M-locus using the CQ method, which was originally designed to identify Y chromosome sequences. Thus, we have shown that such a differential genomics approach can be used to identify male-biased sequences in species with a homomorphic sex-determining chromosome.

In this study, we identified two extremely male-biased sequences: *myo-sex*, a novel myosin heavy chain gene, and a full-length BAC clone NDL62N22. *Myo-sex* is the first *A. aegypti* gene that is passed primarily from fathers to sons and not inherited equally between males and females in the manner of a typical autosomal gene. However, *myo-sex* is not male-limited because recombination can still occur. Earlier work suggests that there is a nonrecombining M-locus in *A. aegypti* (Severson et al. 2002; Toups and Hahn 2010). Thus, neither *myo-sex* nor BAC NDL62N22 are located within the *A. aegypti* M-locus. Genes in the nonrecombining M-locus such as the M-factor are more likely to have male-specific sequences than sequences in the recombining regions of the genome. In theory, genes in the nonrecombining M-locus should be easier to detect with the CQ method than *myo-sex* because they contain male-specific rather than male-biased sequences. However, we did not identify the dominant male-determining gene from the M-locus of *A. aegypti*.

The genome of *A. aegypti* was sequenced with Sanger technology, which has a well-known bias against heterochromatic sequences. Sequencing the M-locus is further complicated by the fact that the genomic DNA used for genome sequencing was derived from both male and female genomic DNA, relegating the M-locus to one-quarter the coverage of autosomal sequences (Carvalho et al. 2003). The estimated coverage of the *A. aegypti* genome sequencing used in genome assembly was only 7.63×, meaning that the M-locus has less than 2× coverage. Combined with the bias against heterochromatic sequences, the actual coverage of the M-locus may be considerably lower. Low coverage of the M-locus is probably a contributing factor as to why we did not identify male-biased genes from the supercontigs, contigs, or transcripts of the *A. aegypti* genome. Thus, the current assembly of the *A. aegypti* genome is uninformative when looking for candidates for the M-factor.

CQ analysis of unassembled sequences, in this case ESTs and BAC-ends, proved more successful than CQ analysis of the assembled genome for the identification of a male-biased sequence. However, future analysis undertaken on new data sets may be more effective than ESTs or BAC-ends. Given that the M-factor is likely expressed in early embryos, CQ analysis of assembled transcripts from deep-coverage early embryonic RNA-seq data may lead to candidates for this sought-after male-determining factor. CQ analysis of genomic assemblies obtained from deep coverage, clone-independent methods such as Illumina sequencing may also lead to identification of gene fragments from the M-locus (Hall et al. 2013).

We cannot rule out that the *A. aegypti* M-locus was recently derived because there is evidence that the M-locus can be found in noncanonical locations in other *Culicinae* mosquitoes, a subfamily that includes *Aedes* and *Culex* (Ferdig et al. 1998; Venkatesan et al. 2009), indicating either translocation or turnover of the sex-determining gene. If so, the M-factor may be an allelic gene variation that is unique to the males or it is duplicated from a nearly identical autosomal paralog, either of which will be difficult to detect with the CQ method. However, the two scenarios described above are less likely in *A. aegypti* because previous studies suggest that the M-locus of *A. aegypti* and *C. pipiens* are linked to the same markers (Malcolm et al. 1998; Mori et al. 1999). There are also cytological differences between the M-locus and the m-locus, consistent with clear differentiation between the loci (Motara and Rai 1977, 1978). Given sufficient genomic coverage and transcriptome sequence data, the M-factor is likely to be discovered with the CQ method.

### Is *Myo-sex* an Example of a Sexually Antagonistic Gene near the Nonrecombining M-Locus?

Our findings suggest that *myo-sex* is functionally important because it is under strong purifying selection. Although the nucleotide identity between *myo-sex* and its closest paralog, *AAEL005656*, is only 83%, the amino acid identity is 97%. Another indication of the potential functional importance of *myo-sex* is its apparently high level of temporally regulated expression. *Myo-sex* is highly expressed in adult males but not in females as *myo-sex* is rarely found in the female genome. However, in the rare females that acquired *myo-sex* through recombination, *myo-sex* is expressed at a much lower level than it is expressed in males (fig. 4). Such reduction of transcript level could result from a number of mechanisms, including the loss of a distant *myo-sex* enhancer during recombination, repression of *myo-sex* expression near the m-locus, or simply that *myo-sex* had evolved a male-specific pattern of expression. Regardless of the mechanism, such an intriguing expression pattern may suggest that *myo-sex* is sexually antagonistic, being advantageous to males and/or deleterious to females. The impact of loss of *myo-sex* function in males and gain of *myo-sex* function in females may or may not be easy to discern in the laboratory. It is also important to point out that the recombinant individuals shown in figure 3 may not be good subjects to investigate the effect of gain or loss of *myo-sex* function because any observed phenotype could not be solely attributed to *myo-sex*. As a significant portion of the chromosomal arm participated in the recombination event, other yet-to-be-discovered genes in the M region may also have been gained or lost. Thus, true gain-of-function experiments such as ectopic expression of the *myo-sex* transgene and true loss-of-function experiments such as site-specific knockout of *myo-sex* are needed to determine the function of this gene in male-specific morphology or behavior. *Myo-sex* is tightly linked to the M-locus and rarely found in females (figs 2 and 3). The potential role of *myo-sex* in the expansion of the nonrecombining, sex-determining region is discussed below in the context of sexual antagonism (Charlesworth et al. 2005; Charlesworth and Mank 2010).

### Recombination Dynamics Near the M-Locus and the Preservation of Homomorphic Sex-Determining Chromosomes

As discussed above, the *A. aegypti* M-locus shares a common ancestor with *C. pipiens* suggesting that the M-locus is ancient (Malcolm et al. 1998; Mori et al. 1999). The evolutionary forces that limit the expansion of the nonrecombining M-locus and thus maintain chromosome homomorphy are unknown (Toups and Hahn 2010). We have shown that *myo-sex* still undergoes recombination, and recombination was detected by screening several thousand individuals. Our transgene-aided analysis of recombination is much more sensitive than the traditional linkage mapping analysis, in which often less than a few hundred individuals are analyzed. Sexually antagonistic or not, *myo-sex* is extremely male-biased and shows drastically different allele frequencies between the sexes and favors reduced recombination with the M-locus (Bull 1983; Rice 1984, 1987; Patten and Haig 2009; Charlesworth and Mank 2010). The duplication that produced *myo-sex* likely happened long ago because *myo-sex* has diverged 17% at the nucleotide level from its closest autosomal paralog despite strong purifying selection. It is thus fascinating that *myo-sex* has not been incorporated into the nonrecombining M-locus in the intervening time. This could be caused by the lack of sexually antagonistic genes around the M-locus that would benefit from incorporation into a nonrecombining region. Sexual antagonism could be resolved with sex-specific expression, thus alleviating the need for a large nonrecombining region (Vicoso et al. 2013). The lack of significant expression of *myo-sex* in the females that acquired *myo-sex* is interesting in this regard. Even if *myo-sex* is sexually antagonistic, the selective pressure to remove *myo-sex* from the female genome by incorporating it into the nonrecombining M-locus may be low because *myo-sex* expression is hardly detectable when it occasionally found itself in the females. On the other hand, leaky expression of *myo-sex* when present in females may confer sufficient selective pressure to keep *myo-sex* male-biased or closely linked to the M-locus.

Another scenario that could contribute to the persistence of recombination between *myo-sex* and the M-locus is that the M-locus may be flanked by recombination hotspots, which may have a high intrinsic rate of recombination. Recombination hotspots are well known in yeast and humans (Gerton et al. 2000; Myers et al. 2005), and they are also recently characterized in detail in *Drosophila* (Chan et al. 2012; Comeron et al. 2012). Minisatellites are associated with such recombination hotspots in insects and spiders (Mita et al. 1994; Sezutsu and Yukuhiro 2000). A genomic analysis of chromosomally mapped supercontigs demonstrated that the band 1q21, in which the *A. aegypti* M-locus resides, has an elevated coverage of minisatellites when compared with the neighboring regions (Sharakhova M, unpublished data). Although recombination hotspots can change locations, the M-locus of both *A. aegypti* and *C. pipiens* are proximal to the tandem repeated ribosomal genes (Timoshevskiy et al. 2013), which could promote recombination. Thus, it is possible that the nonrecombining M-locus is near recombination hotspots, which sets a higher threshold against the expansion of the nonrecombination region.

We also identified a BAC clone that is extremely male-biased in the Liverpool strain of *A. aegypti*, but not male-biased in the *kh^w^* strain of *A. aegypti*. Even in *kh^w^* where the BAC is not male-biased, the BAC is located directly adjacent to the M-locus. Interstrain variation in male-biased sequences is noteworthy as it suggests ongoing plasticity near the M-locus. Comparative analysis between strains of *A. aegypti* will likely allow us to narrow in on the M-locus, which may be conserved between different strains.

## Supplementary Material

Supplementary files S1–S2, tables S1–S2, and figures S1–S2 are available at *Genome Biology and Evolution* online (http://www.gbe.oxfordjournals.org/).

Supplementary Data
