# Supplementary material for: Insights into the Preservation of the Homomorphic Sex-Determining Chromosome of Aedes aegypti from the Discovery of a Male-Biased Gene Tightly Linked to the M-Locus
Source: Genome Biol Evol. 2014 Jan 6;6(1):179–91. doi: 10.1093/gbe/evu002 (PMC3914700; doi:10.1093/gbe/evu002)
Supplement: Supplementary Data [file supp_6_1_179__index.html]

Insights into the preservation of the homomorphic sex-determining chromosome of Aedes aegypti from the discovery of a male-biased gene tightly-linked to the M-locus — Insights into the Preservation of the Homomorphic Sex-Determining Chromosome of Aedes aegypti from the Discovery of a Male-Biased Gene Tightly Linked to the M-Locus — Supplementary Data 

# Insights into the Preservation of the Homomorphic Sex-Determining Chromosome of *Aedes aegypti* from the Discovery of a Male-Biased Gene Tightly Linked to the M-Locus

## Supplementary Data

files

**Files in this Data Supplement:**

- Supplementary Data - pdf file
- Supplementary Data - pdf file
- Supplementary Data - pdf file
- Supplementary Data - pdf file
- Supplementary Data - pdf file
- Supplementary Data - pdf file
