# Supplementary material for: Insights into the Preservation of the Homomorphic Sex-Determining Chromosome of Aedes aegypti from the Discovery of a Male-Biased Gene Tightly Linked to the M-Locus
Source: Genome Biol Evol. 2014 Jan 6;6(1):179–91. doi: 10.1093/gbe/evu002 (PMC3914700; doi:10.1093/gbe/evu002)
Supplement: Supplementary Data [file supp_evu002_supplemental-figure-S2-NDL62N22-CQs.pdf]

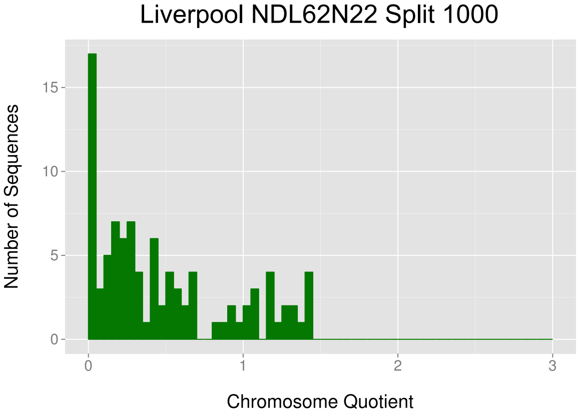

Supplemental Figure S2. BAC NDL62N22 is male-specific across the entire length of the BAC. BAC NDL62N22 was cut into 1,000 bp segments, and CQs were calculated for each segment. A histogram of the CQs of the 1,000 bp segments shows that the majority of the sequence have low CQs indicative of sequences from around the M-locus. Segments with higher CQs are likely repetitive sequences with similarity to other sequences elsewhere in the genome.
