# Supplementary material for: Insights into the Preservation of the Homomorphic Sex-Determining Chromosome of Aedes aegypti from the Discovery of a Male-Biased Gene Tightly Linked to the M-Locus
Source: Genome Biol Evol. 2014 Jan 6;6(1):179–91. doi: 10.1093/gbe/evu002 (PMC3914700; doi:10.1093/gbe/evu002)
Supplement: Supplementary Data [file supp_evu002_supplemental-file-S1-sequences.pdf]

Supplemental File S1

This file contains 3 sequences: 1) the myo-sex full cDNA sequence (GenBank KF150020); 2) the NDL.62N22 BAC sequence; and 3) the re-annotated CPIJ000853 coding sequence.

```
>myo-sex-full-cDNA-04182013-RNAseq
ATCATCCTTCAACGATTCCAGTTTAGCGATCCTTTCATAGACCCCAACCTCGAACCAGCTCAAGATGCCTAAA
CCAGTAGTACAAGTTGGTGACGATCCCGATCCATCCGAATGGTTATACATTTCGGAGGAAATGAGACGCATTG
ATCAGAGCAAGCCTTACGATGCGAAAAAGGCTTGCTGGGTTCCAGATGAAGCCGAAGGATACGTTCAAGGTGA
AATTAAGGCCACCAAGGGTGACTTGGTAACCGTTGCTTTACCAGGTGGCGAGACCAAAGATTTCAAGAAAGAT
CTTGTGGGCCAGGTAAATCCTCCCAAGTACGAGAAATGTGAGGATATGTCGAATTTAACCTACCTCAACGATG
CCTCTGTTTTACATAACTTGCGAGAACGATACAGAGCTAGATTGATTTATACATATTCTGGGCTGTTCTGTAT
TGTCATCAACCCATATAAACGTTGGCCTTTGTACACATTACGAGTCGCCAAAATGTACCGTGGCAAGCGACGT
AACGAGATTCCTCCTCATTTATTCGCAGTTTCGGATGGTGCTTACGTTAATATGCTGACAAATAAGGAAAATC
AGTCAATGCTGATTACCGGTGAATCTGGTGCAGGGAAGACTGAAAATACTAAAAAGGTCATTGCGTACTTTGC
AACCATTGGTGCGAGCAAAAAAGACACAGAAGGAAAACCATCTTTAGAAGATCAAGTTGTTCAGACAAATCCT
GTGCTTGAAGCCTATGGTAACGCAAAAACTGTTCGTAATGACAATTCATCTCGTTTCGGTAAATTTATTCGAA
TTCATTTTACTGCTTCTGGAAAACTGGCTGGTGCTGATATTGAGACTTATTTATTGGAAAAGGCTCGTGTTAT
TTCTCAACAGACTTTGGAGCGTTCTTATCACATATTCTATCAAATGATGTCAGGATCTGTAAAGGGATTAAAA
GAGATGTGCTTCTTGTCCAACGATATCTACGATTATGTTAACGTGTCCCAGGGTAAAATCACTATTCCAAATG
TTGATGATGGTGAAGAATGCATGCTCACTGATGTTGCCTTTGATGTATTAGGATTTACTCAAGATGAAAAAGA
CAACATCTACAAAATAACTGCTGCTGTTATGCACATGGGAGGCATGAAATTTAAACAGAAGGGACGAGAAGAA
CAAGCAGAAGCTGATGGAACGGATGAAGGAGATCGAGTTGCTAAGCTATTGGGATGCGTTACAGATGATCTAT
ATAAGAACTTGCTGAAACCAAGGATAAAAGTCGGTACCGAATACGTTACGAAGGGACAAAACAAAGATCAGGT
TTCCAATGCCGTTGGTGCCCTTTGCAAGGGTATTTTTGATCGTTTGTTTAAATGGTTGGTGAAAAAATGTAAC
GAAACCTTAGATACGAAACAAAAACGTGCTCAGTTTATTGGTGTACTGGATATCGCGGGTTTCGAAATTTTTG
ATTTTAATGGATTCGAGCAGCTGTGTATTAATTTCACCAACGAGAAATTACAACAATTCTTCAATCATCACAT
GTTTGTACTGGAACAAGAGGAATACAAAAAGGAGGGCATTAATTGGGCTTTCATTGATTTTGGTATGGATTTG
CTAGCCTGCGTCGAGCTAATTGAAAAGCCTATGGGTATACTTTCAATTCTTGAAGAAGAATCTATGTTTCCAA
AGGCCACAGACCAAACATTTGTAGAGAAGCTGATTACCAATCATTTAGGAAAGTCTGCTCCATTCATGAAACC
ACGACCACCAAAACCAGGTATTCCAGCTGGCCATTTTGCCATTGGTCACTATGCAGGAGTTGTTTCGTATAAT
ATCACTGGCTGGCTAGAAAAAAATAAGGATCCATTGAATGATACTGTTGTTGAGCAATTCAAAAAAGGTGAAA
ATAGTCTTTTGGTTGAAATCTTCAGTGAAGCCGCAGCCGCTCCGGCGGAAGGGGCAGCACCTGCTGCAAAAGG
AGGCCGTGGTAAAAAAGGTGCAGCTTTTGCTACAGTGTCATCAGCTTACAAAGAGCAGCTCAACAATTTGATG
ACTACATTACAGTCAACCTCACCTCATTTTGTGCGTTGCATCATTCCAAATGAATTGAAACAAACAGGCCTCA
TTGATGCTAAATTGGTTATGCATCAGCTAACATGTAACGGTGTGCTTGAAGGCATTCGTATCTGTCGTAAAGG
ATTCCCAAACAGAATGATGTACCCTGATTTCAAGCAGCGATACCTTATTTTGGCTCCAGCCGCTATGCAAGCT
GAACAAGATTCGAAAAAGGCAGCTGAGAAATGTTTTGAAGCAATTCAACTGGATCCTGATTCCTACCGTATTG
GTCACACAAAGGTATTTTTCCGTGCCGGTGTTTTGGGTCAAATGGAAGATTTCCGGGATCAACGGTTATCTAA
GATTATGTCATGGATGCAGTCCTGGTGTCGTGGCTATTTGTCTCGCACAGAGTTCAAAAAAATGCAGTCGCAA
CGTACCGCGCTAGAAGTCGTGCAGCGCAATTTACGAAAGTACCTCAAACTTCGTACATGGGCTTGGTGGAAGT
TATGGCAAAAAGTAAAACCACTTTTAAACGTTTCTCGAGTAGAGGATCAAATCGCTAAACTAGAGGAAAAAGC
ACAGAAAGCCACAGAAGCATTTGAAAAGGAAGAAAAACTTCGTAAGGAACTTGAAGCGTTAAACAGTAAACTA
CTAGCTGAAAAGACCGCTCTATTAGATTCGCTTTCTGGGGAAAAGGGAGCTCTACAAGAGTACCAGGAAAAGG
CAGCAAAATTAACCGCTCAAAAGAATGATCTTGAAAATCAGCTTCGTGACACCCAAGAACGTTTGGCACAAGA
GGAGGATGCTCGAAACCAGCTATTCCAAACAAAAAAAAAACTTGAACAAGAAATTTCTGGGCAGAAAAAAGAT
GCAGAAGATCTTGAGCTTCAAATACAAAAGATTGAACAGGACAAGGCCTCCAAAGATCATCAAATTCGCAATC
TGAACGACGAAATTGCTCATCAAGATGAACTTATCAACAAGTTAAACAAAGAAAAAAAAATGCAAGGTGAAGT
TAATCAGAAAACTGCTGAAGAGCTGCAAGCAGCAGAAGATAAGGTAAATCACTTAAACAAAGTAAAAGCAAAG
CTAGAGCAAACTCTTGATGAGTTAGAAGATTCTTTAGAACGTGAAAAAAAATTACGTGGTGATGTCGAAAAAG
CAAAACGCAAAGTTGAAGGAGACCTTAAAATAACCCAAGAAGCCGTTTCTGATCTCGAACGCAATAAAAAGGA
ATTAGAACAAACGATTATGCGAAAAGACAAAGAGATCTCAGCATTGTCTGCCAAGCTGGAAGATGAACAAAAT
TTGGTAGGAAAACTTCAGAAGCAAATTAAAGAACTACAAGGTCGTATTGAAGAGCTAGAAGAAGAAGTTGAAG
```

CGGAACGACAAGCGCGTGCAAAGGCTGAAAAGCAACGTGCAGATTTAGCTCGAGAGCTTGAAGAACTTGGCGA
GCGTCTGGAGGAAGCTGGTGGGGCTACATCTGCACAAATTGAACTGAATAAAAAACGGGAAGCTGAACTAGCA
AAGTTGCGGAGGGATTTAGAAGAAGCCAATATACAACATGAAGGTACATTAGCCAATCTGCGTAAAAAACATA
ACGATGCTGTTGCTGAAATGGCAGAGCAAGTTGATCAGCTAAATAAACTGAAAACAAAAGCGGAGCACGACCG
TGCCAATATGTATAATGAGCTAAACAACACTCGTTCTGCATGTGATCAGTTAGCACGTGAAAAGGCTGCTCAA
GAGAAAATTGCAAAACAACTACAACATACCTTGAATGAAGTTCAAGGAAAGCTAGATGAAACAAATCGTACTT
TGAATGACTTTGATGCTGCTAAAAAGAAGCTTTCTATCGAGAACTCAGATTTGCTTCGTCAATTGGAGGATGC
CGAATCACAAGTTTCACAACTAAGTAAGATAAAAATTTCCCTAACTCAACAGCTTGAAGACACGAAACGTTTG
GCCGATGAAGAGGCCAGAGAACGAGCTACTTTGCTTGGCAAATTCCGTAACCTGGAGCACGATCTCGATAATC
TTCGTGAACAGGTTGAGGAAGAAGCGGAAGGTAAAGGAGACATTCAACGTCAGCTAAGCAAAGCTAATGCTGA
AGCACAGTTGTGGCGCAGTAAGTATGAATCTGAAGGAGTTGCTCGTGCAGAAGAACTGGAGGAAGCCAAGAGA
AAACTTCAAGCACGTCTTGCTGAAGCCGAAGAAACAATTGAATCTCTAAATCAAAAGTGTGTAGCTCTAGAGA
AGACTAAGCAACGACTATCTACAGAAGTTGAAGATCTGCAACTTGAAGTCGATCGAGCTACATCAATAGCAAA
TGCAGCAGAAAAGAAACAAAAGGCATTTGATAAAATCATTGGAGAATGGAAACTTAAGGTCGATGACCTAGCT
GCTGAATTAGATGCATCACAAAAAGAATGCCGCAATTATTCTACAGAATTATTCCGGTTGAAAGGTGCATATG
AAGAAGGCCAAGAACAACTTGAAGCCGTTCGTCGAGAAAATAAAAATCTAGCTGATGAAGTTAAGGATCTATT
GGATCAAATTGGAGAAGGAGGTAGGAACATTCATGAAATCGAGAAGTCTCGTAAACGTTTAGAGGCGGAAAAA
GGTGAACTACAAGCAGCTCTGGAAGAAGCAGAAGCTGCCTTAGAACAGGAAGAAAACAAGGTACTACGAGCTC
AGTTAGAACTTTCTCAAGTTAGACAAGAAATCGATCGGCGCATTCAAGAAAAAGAGGAAGAATTCGAAAATAC
CCGTAAAAATCACCAACGGGCACTTGATTCTATGCAGGCTTCATTAGAAGCTGAGGCTAAGGGAAAAGCCGAA
GCTCTACGTATGAAAAAGAAGTTGGAGGCGGATATCAATGAGCTAGAAATTGCCTTGGATCATGCAAATAAGG
CAAATGCAGAGGCTCAGAAGAATATTAAACGTTACCAACAGCAACTCAAGGATGTTCAGGGGGCTCTTGAAGA
GGAACAGAGGGCACGCGATGATGCTAGAGAACAACTTGGCATTTCTGAACGCCGCGCTAACGCTTTGCAAAAT
GAACTAGAGGAATCAAGAACCTTACTCGAACAAGCAGACCGTGGCCGTCGACAAGCTGAACAAGAACTTAGCG
ATGCACATGAGCAGTTAAACGAAGTTTCAGCACAAAATGCATCTATTGCAGCTGCTAAAAGAAAACTTGAGTC
TGAATTGCAAACTTTGCACTCTGATCTAGATGAGTTATTAAATGAGGCTAAGAATTCAGAAGAAAAGGCTAAA
AAAGCTATGGTTGATGCTGCTCGACTAGCTGATGAGCTAAGAGCAGAACAGGATCATGCACAAACGCAAGAAA
AACTACGAAAAGCTCTAGAGCAGCAGATCAAAGAACTCCAAGTGCGGTTAGATGAAGCAGAAACAAATGCGTT
GAAGGGAGGAAAGAAAGCAATCCAGAAACTGGAACAGCGTGCGCGAGAATTAGAATCTGAATTAGATAGCGAA
CAAAGGAGGCATGCTGATGCACAAAAGAACCTCCGTAAATCAGAAAGGCGTATTAAAGAGCTTACTTTCCAAT
CGGAAGAAGACCGTAAAAATCATGAACGAATGCAGGATCTCGTTGATAAACTGCAGCAAAAAATCAAGACATA
TAAGAGGCAGATTGAAGAAGCCGAAGAAATAGCTGCCTTAAACCTTGCAAAGTTCCGTAAGGCACAGCAAGAA
TTAGAGGAAGCAGAGGAACGAGCTGACATTGCTGAACAAGCCGCAACAAAATTCCGTTCCAAGGGAGGCCGAT
CTGGGTCGGTTCAACGGGGTGTCAGTCCAGCAGTGAGTACAGTGTAAGTATCTATAGAACGTCTATTCTTATT
TAATTTGTATAGATAATCATCAGAAGTCAGCAAGAAGTAGCTTAATTGAGGCAATCCAATTGTATGGTACAAC
TTCC
>NDL.62N22 05-03-2013 (BAC-ends 33227393, 33227396) (Whole sequene
Generated from PACBio sequencing and assembled by HGAP. corrected by
removing the cloning vector and assembling with megamerger)
CATTAGCGCTTGTAAAATGTTTGGTAAGTATGAAAACCATTTGGGCAGATTTGATTTAACGGAACTTGTGCCC
TTTTTTCACTATGATCAATCATCTTTTTCAAAGCGATTAGGCTCAAATGCAGATGCGTCGATAAGAAAAAAAA
AATTTCATTGCATCTAAACCAAAGCACCAAAACATCAAATCAAAAGCCTTTCTAAAGGGGGAAATGATTAATG
CATCTTCTTGCCGCACAGTCCATCAGCAGTTAACAAGCAAGAGGCTAGGGCTCCGAGTCGGGAAAAAAGCGAT
ACAATGCATCGAAAAGAGGGAAGAAACAAAGCCTACTTCTCCAGCATTGGGCGATTGGAGCTGGCCGATCGAT
GGAATAATAGGCGTAAATGTGGAATGTGGGAAACAACCTTTGCTCGCCGGAAACTGCTCCACTTTTCGGATCA
AGTGGAAGAAGGGGCGAGAAAAGCGGACCACTGATGAATGGAGCATGCATTCTGATCGCGGAAGAACCCAGTT
TACGGGAGGTTTCTCTACTCTCAGCTGATGGTATTCAATTTTGAGGGAGGAAGTTAAAATGTGGGTCACGAGA
TTTTTTCGGGGGTAATAACAAACCAGCTGAGCGTCAAAAGCGATGCTTTCGTCGGCATTAGCCGACATTAGAG
GTTGGAACGATATGCGTAGAATGATTGAAAAGTGTACTTACGCCCACGCGTATTTCGAAGCGTTCCGTTTGCC
GGTGAAGAGCGGTGACAGTTCTGCCCGTAGTCGTATTAGAGTTTTCACTTTGTCTGCTTCCCCTGCAAGAAAA
GATGATATAAATGGAAATGAAATTAGTTTTGTTGGATTCATAAATAATTGAAGTTATTTGTTAGTATTCTAGG
CTGAGCTCGTGTTGCTGCTTTAGATAACATCCCTATCTCTGATTTTGTGCGATAGAACTATACAAATTTATCG
TTTTTTCCTTCAGAACCAAGATGTTGTTCTTAAGGTGTTTATTTCTCTACCCCTTCCTCTACATATGAAATCT
GGATAACATTGTCAAACGATAAAGTTCCAATAATTGACCACCATCGTACCATTCATTGAATTGTAGAAACAGC

ATTTAAACTACAGTTCAAACGACACGGTTAAATCATCTAGTTTTTAATGAGTGCTAGTTTGACAGTAAATCGA
TAAGATGGATCATAAAACTTATCGATGGTCTGTTAAACAATACAAAGCTAATATCAGGTTCTACCAGAAAGTG
TGTTTGGATGCTTTGACCATTGACAATAATTAATTAGATAATTTTTCACAACAATCGTGGAAAAAATGAAGAA
CAAAGAGTGGTTTTTGGGTAATCGATAAAAATTGATTTACGAGTTATGAACTCAATACTCAAGTTTTTTTTTG
GATTGTTATCATACGGAAACCGTTTCCGTATGAAAACAACAAACACACGATGATAGTTCCTTTCGAGGTTTTC
TGTTGGAGTGAGTGCTTTGTGGAATCTCTTTTTGCTTCGTAGTCGCGAATAGGTCTTTTCATGTTACAGGTCC
CTCTATGCATTTATTTACATCGGTGGAAGATCAGTGCAAACAGTAGAAGCATTAACCTAAGAATGCTAATGTC
GCTGCTGTTCGTGTTACCAACATCTAGTTTGCCACGAACTGACAGCATGAGTACCGGGCATCAAAGTGTCATA
TTAATTACAACAACCAAAAAAACGACATGGAATTGAACAAACAATGAAAAACTATAATTTAAGGTAATAATAA
TTAAAATCAATTTTGTGACAACAGCGCACCTAGCGTTCTACTACTAATATTTCTATTGTGCAAACACGAGGCT
TTCATTTCCATAGAAGAACTGTCAAAGAGCTTCCCGATCAGCTGATTTGGAACGTCTTGTGAAAAGGCCTATA
GCGTTGTTCCTATACACGGAGACGGAATTCAACTCAATTTTGGGTTGTTTCAACGCAATTCCGTAGTTGAGCC
CAATAACTCAATTTTGGCTAAATTTCCAAGGTCCCCACTAGGTAGTTTGGCTTTAATTCCATTAGAAAAATAT
ACTCAATTTTGGGTTGATTTAACGCAAAATTGAGTTCTTTAGACCCAAACATAAGAAAAGTTGCATTTACCCA
AATTTGGCTTATTGGGCCAAAGTACCCCCGTTGGGTAGATGCAGCTCTCTCTATTTTTGACAACATTCGTGTG
GGAGAAAGAGAAAACCGAGAGATTTTGGGTTGAAACTAAAATCGATATACTTAATTTTGGGTAAAGTAAACTC
AAAAATTAGGTAAAAATATTTCTCCGTGTAGGATATATCTGACAGTCCGAAAGGTACACGGAAAACATAATTC
AACTAAAATCTGAGTTTGAATCAAGGGTTGCGGCAAAGTCTTAAAAACCAGATATATAAGATTTGGTACTTAA
ACAAATACAAACAATTTAGAAATTAAAAAAAAAACATGTATTTTTGGCCTGAGCTAAAGCATTTAGTAGAAAC
ATTGAGATAGGGTTTTATGACTCAATTGTAAAGAATTCCCATTCAATGTTCATAGAACAACAATTTGAAAACC
GTTTTAGTGAAATGTATTCATAAAAATTTCCTTAGATGAAATCAACCACATCTACACCCATAGAACAACAATT
GATCTTTTTTTTCAGTAGACCCTCAGCTTGTTTTGACAACTGACCTAGATTCCATGTTAGCCAGTGTCCAACC
AAACAGAGTGAATACTGAGAAACACGCTTTTATCTCTTCTACTTTCTTCACACACAATCAATCCTAACCCGTT
TTGGTTGACTGTCCATCCTTTCGGGTATAAAGAGCAGTGCCATTGCACGTATACGACTTTCTGTGTGAGTGTA
GCGATCTTATGTGCGCACCCCGAAGAAGGGAAAGCTTTCCCTCTCAATGGTGACGACGATCGAGCTGACGATC
ATCGCGCGCCGAGCGATCGAGTAACAAAAGCCACCCTCTTGCGCTAGTGGTAGCCCGAGCAAAACTCGTCCAG
GCATCCACCCCTTCCATAGCAGCATTCGAAAACATCAACAACAACAACGACTATGCAAGGTGAATCAAAGTCG
AGTTTATTGACTTTTGCCAAGTCCGTCTTGGCTCGCCTGGCGAGGTCCGTAGTGGCAGGGCATCTACTAACTC
TCGGTAATAGGCCATAGTAGCCACACGTACAGCAACTGCGTCATCAGCTGACGACAACGAATGGCACAGTTAG
TGGAGAAAGGGCAATCGGTTGGTGGAGGTAAAACCACTACTGAAATTCATTAGGACGTAGTTCTTCGGCTTCA
GGGTAGTATCCGGAGATTTGAGTGTTGATAAGGGGTCAAAACAACAGTATATGATATGATTGACTAATTTTTA
ATGTATGAATATGTTAAACCATTCTGGAAATAGTGCAAACCATTCAGCAAATGGCACAAACCATTCAAGAAAT
GGTAAAACCATTTAGGAAATGGTGAATGACTCACTCCTACGAAAATGAACCATTTCGTGACCCCAACGGCTTC
AACCCTGATTCCACGCAGTAGCAAAACTCCCAAATGAAAGGAAATGTGCGCCACTAGGAGGAGGGAGGGAAGT
AATATTTACAGAAGCAATCCGTTTTCTAAGCAACTACCAAAAAAAAAAACGAAAACAGAAATCGTGGGGATTG
TGAAACCCTGCTCGACTGCGACTTGCTAGTAGGTAGTGGCTCGACCAAAAAATCATTTCGATCATTTCGCTTC
TCGGAGCGTGGAAGGGGTTGCGTTTTTAAAACCATTTGTTATTTTGGGGTTTGTTGCTGTGACTGTTGACTGC
TTGTTGTGGTGAACAGATTTTTTTTTTGGGGCCATTCGTTGGACCTCTTAACGGACACCAACAACGGGGGACC
TATTACATCAGTCACCGCAGCAGCAGCTGTTGTTTGTATGCGCGAGACGAAATCGCTATAGGATAAATTAAGT
TGTTGTTATTTGTCCAACGGTTGGCGCTGTTTGATGGGGTAGTTTTGCTTGGGAAAACCCCCTCGGTCAGAGT
GAGAGAGAAAGGTTCGGATTATTGGTGGTATGGTTCTGAGATCGACCACCAGTTGATGACCATAGGCACGGCA
GGCCATGTATGACAGCGATCACGTATAGAAGTCTTATGTAATATAGAGTTTCGTGTGCATGGCGATGACTCAG
TTCGTAATGCATTATGCTTCTTATGGCCGAGGAAATTGCGGGAATGTTTTCGAGTGATTCATGTGAGTGAGGA
ACAGGGCTTGAATCCTAGTTACGGTGAGTCAGAACAACATATTTTGCGATCAGGTGTAATCTGACTCATCTTA
TGGCAGAGATTATTGTCTATTTGACATTAAAGTGTGATGTGATGTATGGCTTTAATTTTCTGGCATAAAAACG
ATGAGATCAATCAAATATCATATGAAAGTCTGTACTGTATAAATCATAATCATTTCGGCTATAATTATTACAA
ATTTAAATTGCCTTCGATTGGAATTCCATCTGTTTGTTAAATTTTCAACGTTTTCTTTAGATTTTTTGATTTG
AATCAATATTTTTACTTCCAACAGATTTAACTAATTCAATTCAAAATTAACTGATTTCTGTAATTTATGTAAG
ATTTACATCAATTCTTATTCCATACTCAGTTGTAAATGCAAATAAGTCCTTATCCAGCTATATACGAGCGCCA
ATGAAAACTCAAACTCACTTTCTGGCAGGGTTCAATCGATCTGAAATTTGGCTTGGGTCATTTGACAGCAAAT
CTATAGGCACGATCATCAAACTTATCGATCAGCTGTCAAATGACACACACGGAGAAATCGAAAAACTCAAATT
TGTGTATTTTTAAACTTATTTTTGAGTTATTTTTCTCTCCCCATTTCATTCGCTCCTTCTATTGTTGTCAGAG
AGCGAAGAGCAAAACAACCCAAAAACCGAACCTTTGTGCATTACCTCAAATTTGAGGCATAGTACTGGAAGGA
GTTATTAGATCTGCCGGTTGAGTGAAAAAAAAACTTAGCCAGTAGGTATTTTTTACATGGCTGCAAATGAAAA

CAACTTCTACCCCATCAACTTAAAATAAGGTTAACGCATGGGGGGTTTATTGGGGGGAATTGAGTGGAAACGA
CTCTTTTTTGAGTATTTAATTTTCTCCGTGTACTGTCTAATTGACATTAACGGGTGATCTGATGTAAAAACGA
TAAGATCGATCAAATATCCAAAGAAAGTCTGTACCACAAAGCACAACCATTTAGGCTATTTTATTAGAAAATT
TCAATCACTTCCGATTAAATTTTATGTTTTTTGATTGAATTTTATCTGTTTATTACATTTTTATGATTTTGTT
TCGATTTGTTTTCAATTTTTTTGGTTTGAATCAATATCTTCACTTTCAACAGATTTTAATAATTCAATTCAATG
TTGTCCGATTCCTTTAATTCCCATTAGATTTATATCAATTGTTATTTCATACCAAGTTGTAACTGCAATCAAG
TTCTTCGATCTTTTTACTTACAACATTCTAGACAATAGTGTTCTAAAACTTTTGAAAATCCTTTAGTTATATT
CAATAACACTGTTGAGCTTGTTCTTGCAAAAAAAAAATCTTATCGAATTATGGAAGAATACTAATGACGCTTG
AGAAACCGCTTAATGATATCAGAAAGTAGCACTTTAAAAAAAAATCGGTTTTAGAGAGGTCACCCTATCCTTC
TTTAGCCGAGTGGTTAGAGTCCACGGCTACAAAGCAAAGCCATGCTGAAGGTGTCTGGGTTCGATTCCCAGTC
GGTCCAGGATCTATTCGTAATGGAAATTTCCTTGGCTCCCCTGGGCATATAGTATCATCGTACCTACCACACG
ATATACGAATGCGAAAATGGTATCTTTGGCAAAGGAAAACTAGAAGACTAGAACACTAGAAAACTAGAAAACT
AGAAGACTAGAAAACAAGAAGGCTAGAGGACTAGAAGACTGAAAGACTAGAAAACTAGAAGACTAGAATACAA
GAAAACTAGAAGACATTAAGACTAGAAGACAAGAAGATTAGGAGACTAGAAGCCTAAAAAACTAGGGTACTTG
AAGACTGGAAGACTAGAAGACAAGAAGACTAGAAGACTAGAAGATTAAAAGACTAGAAGATTAGAAGACTAGA
AGACTAAAAGACTAGGAGACTAGGAGACTAGAAGACTAGAAGACTAGAAGACTAGCAGACTAGAAGACTAGAA
GACTAGAATAATAGAAGACTACAAAGACTAGAAGACTAAAAGACTAGAAGACTAGAACATTAGAATCCTAAAA
GACTAGGGGACTTGAAGACTGGAAGACTAGAAGATGAGAAGACTAGAAGACTAGAAGACTAGAAGGCTAGAGG
ACTAGAAGAGTGGAAGACTTGAAGAATATAAGACTAGAATAATAGAAGACTAGAATACAAGAAAACTAGAAGA
CATTAAGATAAGAAGACTAGAAGACTAGAAGCCGAAAATACTAGGGGACTTGAAGACTGGAAGACTAGAAGAC
AAGAAGACTAGAAGACTAGAAGACTAGAAGATTAAAAGACTTGAAGATTAGAAGACTAGAAGACTAGAAGAAT
TGAAGACTAGAAGAATAGAAGACTACAAGAATAGAAGACTAGAAGACTACAAGAATAGAAGACTAGAAGACTA
CAAGAATAGAAGACTACAATACTAGAAGAATAGAAGACTAGAAGACTAGAAAACTAGAAGAAAAGAAGACTAC
AAGACTAGAAGACTAGAAAACTAAAAGACTGGAAGACTAGAAGACTAGGAGACTAGGAGACTAGGTGACTAGA
AGATTAAAAGACTAGAGGAGTAGAAGACTGAAAGACTAGAAGACTAGAAGACCAGAGGCTATGAAGGCTATGG
AAGTGCTCATAAGAACGCTAAGCTAAGACGCAGAAAAAGGCCACCCTATCCAAAAACTATAAGTAATATCGTC
TAAATTCTTCTTCGTTTTGGTGTCATAGGCATAATTTTCTAATGATGCTATGGAATAATACTGAACGATCAAT
AATTCTCCACAAATAACGTAAGAATTATTAATTCTCAAACGAGCGTAGGAAAAAAAAATGAATAAAGTGATGT
GACTTCCGATGATACTATGGGATTGAAGTGAGAGTTTCAGTTTCAACTCAGTTTCGCCTTAGTACACACATCA
ATCTTATCGATAAAATGTCAAATGAGGCAAGATGTATTATTGTTTAGTCGCCCTCGGAATTCGAATTTGATTA
GCCAGGGTTCAAATTGTTTTTAGTGAACTTTCAATTGATTTGAAATGGCTTTCACATTGTGAAATAAGATTTT
GATGACTTTTGATCGTTTATTGGAAATTATCATTCTCCATTGCCTATATCCGTCGTATCCATATCTATTTCGG
TCACATTCAGTTTTGATTCGTCTAAGGGAGGATTAAAAATGACATCCATCGTTAAGGGGAGAGGGTTTCTACA
AAAGTGTGACATTACATGTATTAGGTATTGAAAAAGGCGTGACAGAGGGAATCCAGAAAACCAGGAAACGAAA
GACGTAATTTTTGAATTTTCCTTCACAATTGACGGTTCACTACCCACCATAAGTATGGAATCGCATCAGCTAG
TATTGCAACACCCCAGTTGAATATTGCAGCACCGCGAAATTAAAACTATAATGTTTATGAAGCAGTATTTCGG
ACTCATTACTTTCGGCAAAGTTGCGAACTTCAGCAAAGTTGTTCAGCAGCTTTCTGGCGCCCATTAGGTAATA
TTTTCAGATGCGCAATTTTGCCGCTACGTGGCACTAGTATGCATGTAATTTGGTCATATTATAGCAAGCTCTA
GCTCATAATCCAGACCACCTAAAAAGTTCGTGTTTTCAGCAAAGTTGTTCAGAAGCTTGAAAGCAATTCTACT
GATTCTACTAAAATATGACAAAATTACATATACAATAGCGCCACGTAGCTGCATAATTGCTAACTAAACAGTG
CTGGTTCCAATTTCTTTCAAGCTTCTGACCAACTTTGCTTAAGTCGCGAACTTTCTAGGGGGTCGGAGTCATG
AACTTGAGTCTGCTAGAATATAATCAAATGACATGCACGCTAGTGCCTTGTAGCGGCAAAATTACGCACTCAA
AACACCAATTTATGAACACCATAAGGCTTTTGAACGACTTCGCTGAAGACCACAACTTTGTAGAAAGGGCTCA
ACAGCAACGTTTCTGAAAAAAAGACTCAAGCCCTTTTGGGCCCTATTCAAATGTTTGTCCAGAAATGCATGAC
AACTACTCACAGTGCTGCAAAAAAAATCTTTGACACAACAATCAAAAAAATCGTTTTCTACTCCGAACATTACG
CACACATTGATGCGAAAAAAATTGTTTTCTATGTATGGACGATAAAAACACATTGTTTACATTCTGAATTACA
TCCAGTCGTTAAGGGAAATGTATTAAAATTTCGTGAATTTTAGAAGTTCTACGGCGTTTGATTCGAATGAAAT
CGTTAAGTTTACAGAAGGAGAGGTGATCAGAAATTTGAATCGTAATGTGACGTCGCTTAGTCGATCGCTAATC
AGATCTTGTTCGGTACGTCTTCGTCCATGCGATTTCGGATGAAAAGTGCGAAGTTGATAATTTAACTAAAGGT
TATTTACCAAAATACACTATTATCATTGCAGCTTTTATTAAATTGCACTTGGAAATTCCACTTGGTAAATGTA
TACTGTAGATTAGTTCCAATTTCCGCTCTACGACGGGAATTAGCACTTGCGACGAAATAGATCAAAACTCTAA
TGGTCACAATAATCAAATATAAGTAATTGTTATGATCAAACGATTTGATGTTTGATTTGTGTCGTTTGACAGT
TCAGCGATAAGGTCTATAAACAATCTTACCGATCAACTGTCAAAGGACCGATGCCAAGGGTCAGTTTCTTTGA
TATTTTCCAGAAAGTGCGATTGATTGTGACGACTTCCTTCAACGCACGTAAAATTCTTAATGTTCATTTGCCT

AGACTCAAATGGTGATATTCCACAATTGATTAAAGAAACGAATCAGAAACTCAAGTAACCACCCTGACGTTTC
GATTTTCAACCCGCTCGTGTGATCGTTGCAACTAAACTGCAGTTCTCCTACCTCCACAAGGCGACGGGGGCCA
ACTAGATGGATTTGCGTGGTGCTTCTCGTCAGAAATCAGCCATCGTGATCATGACCAACTGGTTGCCGTTTCT
TCAAGCCAGGTTCAAGTTCAAGACTAGCATCGGCCTACCTAGATGCAATTGCGCTCGAGCTCGCGCCGTCGAG
TGCACTGCACTTTTGGCGTAGACCGCAGCACGAAGCCAAGGTTCGCCGCTTGCTACGACGAAGGCGATCGATC
GAGTGCGGAAAAACGATTTTCCTCGCACTTGCATTTTCACCCTGCAGCAGCAGACTACCACCGAACCGTGTTG
GCCGTATTCGCCAAAAAGCGGTGCAACGTTGCAACGTTTGGATTCTATTCCAAGAGCCGAGCGAGCAACAACA
ACTCTACAAAGATGGAATTTCCTCCAACGACCAACGACCACGATCATCGCCGTCGTTATGTAGGCGAAATCTA
GCGCTGCTGTCAGAGCATTCCATGCTTCTTGCCTGTGAGATGCACTAAACACAGAAAAACTTTCACCACCAAG
AGTTCGATGATCAGCGTGAATTATTACCCCGCAGCTCGGTCTTCTGACAGAGCCCGGTATGGACGAGGGGTCT
GCGGAGTGTGAGTGTATCGCTCAGTAATAAAATATGAAACTATGCTACTAACGGCGAACTGCAGCTGCTGAAT
GCTCTTTTGTAAGTGGAGATGCATAGATTCCTAGATATTTGATGCACTTTCCATTTTTTTTAAGTAGCTAGAT
GGAACAAACAATCGAGCACTTCAGCACCGATGCGCACGGTGGCTAATGAATTGGAGAGCTAGCACTGAAAGAA
ATTGTTACATTATTGGAAACGATAAAATTTGATCGTTTTACTGTCAAGATGTATCGTTTGTTTGCAAAAGTCG
ACACGACAGAAATTTTACGAAGAATCGAATTTAATTACTTCTGTTACTTGAACTCCACAACCTGATTGTGTGC
GCTTGTTCGCTCCATGCCAGAAGCTATTAATCAATCATTTTCACTTTCGCCTGGGGACGTGTACGATTCCATC
GGGTTGAAGAAGCGTAAGCAATTTTGAAATTATTCCTCGCTGATTACACGTGTCTCGACCGATGGAAGAATAA
TGCTGCAAACCTAGCGTCGATTGGTATCATTTTTTTCCGGGGAAACAACTTTCCAAAAATTACCTCTTCCCGA
AACCCACTTCTTTTGTTTGCCAACCTGCAATGCGAGTGTGTACGCGCAAGAAGTGTACGATGAGCATACACCC
ATCAGCCTGCGGCACCTCTCCACCGCCATTCTCGCTGTGGTCTTCTGCGCGGTTCTCTACACACAGCATAAGT
GGAGACCGCGGCGCGCAACAAGCGAAGCCATTCTTTCCACCGTGGCATGCCATGGCTCCGTCCGGATCGACGG
CGTGACCCACTTTAGGCTACTTTTCGAACAGAGCAGCAGGCGCACAGGGAAGTTCATTCCGAAAGCGGAGGGT
AACCATTTGTCATACGACCCAGTTTTGGTGACATGTTCTTGCGAGACAATTTGGGGAAGTTTTAACAGGTGAT
TTGGAGGGCGTGCCTAACAATTACATTTGCGAGATGGGAGACTATTCTGAAACTTATCGTTTTTCTGTCAACC
TCTCTTGTTTGATCGTTTTTACGAATAATTAGTGTTGCAGTCTCTATCATCAAAGCCAAATAATCACTTATCG
TTCTGTTGCTATCAGATCATTATGATCCCTTGAGCATCAAATGTTATCGTTTTCTTGTTAGTTATTCATTATC
ACTCTTAAATGTAACGTTTCCAGCATTTTGGTAGCAAAAATAGTCATCAAAATAGAGCTCATTTTCTGGTACC
AATCTACCTGGATTGTCTAGAAAAGTTGATCTTTATGATTAAGAATGAATATTATTGATCATCTGTCAAAATA
CACAAGCCAGACGTCAGATTTTACAATATCTACCTGACTCCAGGTTGGATTGTAGTCATTATTGATCACGCTG
TCAAGTTGATCGACTGTTTACCTGCAAAGTCTTTCTGCAACCGATAGCAATATGGCGTTAAAATCTCGTAATC
GCACTCATCAAACATCTATAGTTACTGCCATTGCTAACTCCATGGCGCTAGAAAAGCATAGTAAACGTCATCC
GCCACTAGACGAATAGATACTTTCATTGTCACCAGCAACAGCCGTAGTGGTAGATATGTAAATTTTTACTTTC
ATCGGAGAATTGCGCGTCTCTTACCGCGCCCTCTGTAGACTTCTTCCCAGTAATTATATTGAGGTTGACAGTA
AACCTCGTTGCTAGAGTTTGGCGGGGACTTTAGGCGTATGGCAAACTATTTTCGAACCGATTAACATATTTTA
TTGGCCGCTGCTGTTGCGACAGAGTCGGGCACCGCCGCCCATATATGTGTGATATGAGTCAGCATCAATACCA
ACTGAAGATGCGGGTTGTGCTTGAGAAGAATTGTTCAAATCGCCCGTGAGAACTGAAACCCATTTCGCGGCGA
GGAAACTGTGTCAATGGTTACGGCTTTGGCAATGACGAGCATCTGAAAGTTCTTCGAATGCGGCTGGTATAGG
GTGTTCAGGCGTTATGCCGATGATTTGACTTAAATCGTCTAGTTTTCATATTTTTGATTCAGCTTATGTTCTA
CGATTTAAAGTTTGGCTTGTGCAGTTTGAAAGCAAATCGAAAATGTTAATGGACGATTTTATAGTTTTGCTGT
CAAACGATAAAAACTAAAAGTTAGCTCGTTTCATCCTATATCGACTGACAGTTGATCGTCACGACGGCAGATT
GTTTGATTCCTACCGGATGATAACATGACTAGATTGTAACTAGGTCAGTAGCCTGCGGTGTGGAGCAGAAGCC
CTGTTGTTTTTGTGTAGTTAGTTTATTTCATCAGGTTATGGACCCAGGAACGTCTGGAGGACGCTGAGGCAAC
TCCGATGAGGAACCCGAACCACGAGGAAGCTCTTAGACGATGAGGCAGAATCTCACTGAGGGAGTAGAGCCTG
GACAGCAGGTGGCTTGGACGAGATTGGAAGAGCAGAAGAAACTTGAAAGTCAGGAAGAGCTTGGAGGACGGCG
TGGTTTGCCCTGAGGAGTTCGGCAGCTATGAGAAGCTCGGTGCGAGAAGGAGCTCAAAGGACAGATGGAGCTC
GTTTGCAGATCTGTGGTTCGAAGGTTGTATGATGAGGAGAAGCGTTGGAGAGAGGTACATCGCACACCCTGCA
GCGGACATTTCTGGGGGTAAACGTATTGTAAGAAACCGGTAAGCAAATTAAATTGTAATGTATACCCAGACAT
GCAATAAAAGGAGAAGTGCTGATATGGTAGCGATATCAGTCTGTAGCCTGCGGTGTGGAGCAGAAGCCCTGTT
GTTTTTGTGTAGTTAGTTCATTTCAACAGGTTATGGGCCCAGGAACGCCTGAAGGACGCTGAGGCAACTCCGA
TGAGGGACCACGAGGAAGCACCTGGACGCTGAGGCTGACCCAGACAACCAAAATGTACGTATAACGAAATCAC
CTGGAGGCTTTATATGTGCAAAATTTCACTTATAAGATGTCGCGAAAAGGCCTTCTACGTACAAAAGTGGAGG
CGATATGCGTGCATATATTATGTGATGAATAATAACATACAATGCGAGTGTATAAATATTCTACCGAACCAAC
CTGTGATCTTATGCGACTTAACTTATGATAACAAATATTGTTTTGACAGCTGCTACGGATTGATGCGACTTAC
TTTGTACGAGTGTACGAGACGAAACAAAATGTATAAATTAACACAGAAAAGAAGTGTTTTATGCGAGGAAACT

CATCAAACTGACAAGTTTATCCACAGTGTTTTGCGGGTTTGATGTAATTAGTATGAATAAACGAGTTGTAACG
TGAACTTTCATGCGATTTCTGGTTGTCTGGGGAATCTCGCTGAGGGGTTACGGGAGGTGGCTTGGACGAGGTG
CCCAAGAAGTTGGAAGCCAAGATGCTGAGAACGAGGCTGAAGGTGCGGATGCTGGCTAATCGGAGAGGACGAG
CTTGGAAGAGCTGGAGGAACTCGAAAGTCAGGAGGAGCTCGGAAGACGGCGCGGTTTGCCAGGAGAAGCTCGG
TGCCAGAAGGAGCTCAGAGGACAGATGGAGCTCGTTGGCAGATGTGCGGTTCGAAGGATGTGTGATGAGGAGA
AGTGTTGGAGAGCAGGTTCATCACACACTCTGCAGCAGAGATGACTGTAGGTAAACTGGAGGTATTGTAAGAA
AGCGATAAGCGAATGAAATTGTAATATGTACTCAGACATGGAATAAAAGGACAGGTGCTGATATAGTAGCAGA
AACCTTAAAGCTTTTATTTCAACATGATCAAGGAAAGAATGAGTCGAAAATCCGCGTCTTCTCATTTCAAGAG
AGAGAAGGATGTCCATTAGGGTGCGGCTTATTTTTCAAAAGTTCTCAAAACCGAAAATTCGTGTGCTCTACTG
AATTCAAATCACATTAAAAGAGAAACCTCAAAATCTGAGCCAAAAATATTAACATTTAGAGGTGGCGCAAGCG
TGTTGAAGGTGAATTTTCAAGTTATAAAAAATGACCTGCAGTTAAGTAAACATAACTTTGTTATTTTTCAACC
GATTTCAAAACTTTTAGCACTATTTTCTTTGAAATTAAATAAAAATTTTGTAGAACATCGAATTTGTCTAAAA
TCAAAACTAGTCTTAGATATTTTTATCCAAATTTTTACTCATCTTTTTGGCAATCATTTTTGTTTGACCATAA
CTTCATTAAAACTAAATCGATTCCAAATCTTTTGACATACTTTTGAAGCAAATTTATTTGTTTTCAAACTGTA
TATACAACATTTTTTTTCTAAAAAATATTTTTGACTTCAACAAAAACTACCCAAAAACATGATTTTTCAATGA
AAACATCAAATTTCTTTGGATTATTTAAGTTTTTTTTGCTGAAAATAGCATTTTTTCTATAAAATTTAAATAT
GTAAAGCATCTTGCCTTAATTACGAGTTGAATAGTGCATATATTTTTTAACATATGCAAACATAATTTTGTTT
CAAAATTATTTAAAAATAGTTGCAAAGTCCTTCCCAAAGGTTTAACAAATGAGAAAAATCAGTTTCAGCTTAA
GAGACAATATTTAACTGCCAAAGATTGGACAAAACTGGCATTTTTCGTTAAAAAATAATGTTTTTGTGCAGTT
TTTGCTGAATGACTTGGTCAAAACATGTTTTTAGTGAAATGTGATATATGAAAGGTTTGAAAACAATTAAATT
AGCTTCAAAAGCATGTAAAAATATTTGGAATCGGTTGAGTAATCATGAAGTTATGGTCAAACAAAGTTGAAAT
TTTTAGTAAAATAAGGAAAAATTTGGAATAAATTACTTTTAACTAAAACTTGTTTTGATTTTAGACAAATTTG
GTGTTCGACAAAGTTTTTACAAATTTAATTTTGAAGGTATTGATGCTAAAAGTTTTAAAATCGGTTGAAAAAT
AACAAAGTTATGTGTACTTCACTGAAGGTCATTTTTTATAACCTGAAAATTCAACTTCAAGACGCTTGCGCCA
CCTCTTAATGTTAATATTTTTGGTTCAGATTTTGAGGTTTCTCTTTTAATGTGATTTGAATTCAGTAGAGCAC
ACGAATTTTTGGTTTTGAGAACTTTTGAAAAATAAGCCGCACCCTAATGTCCATTCGCTTCATACATACGCGC
CCAACAAATTGATCTATATGATGTTGAAATGTCGTCTACTGAGATCAATTATAGTCCATGTCGATTAGCACTG
TTGAAGAATTGCCCGCTACAGTGGTGAAGTCTGTCGAAAGATGGCATTGACTAATCGTCCCTTGAAACAGTTG
ACGTTCGGATGTAGAAGAATAGTCCGATGAACTGCGCATCACATAGCTTCATCGAAAAATAGTTTGTTCAATT
TTACATAGGATACGGTCAACATTTCCCACTGAATTAATTGATGCTCAAGTGGCATCTCTTCTTTAAGGATTCA
CCACTACACAAGTACGGTGTAGTTCGCTGTATCTAGCTAACTGTTTGTCCATCAGCTCAAGAACTTGTTCAAT
TGCTATCCAATCGTACCATAGAAGTTTCAAGATATAGTGACATGCTAGGTGACGGACGGAGAGTTGAACGCAT
ACGGGATAGACTTGTATAATCTTGGTGAGCGAACACGTTGAACCCTGGTCACGTCCTTGCAACAGCTAGGCTA
TTCTATATGAGAAATACGGACTAGTATTTAACTCGATAGCAGAAGCGTTAGTCATTAGTCCACCGGCACTGTC
TACCAAATGCCTTCTGGCTTCAAACGCTGAAGATCGTCTGATCTCCAATGACGAGCTTTTAGTAGCGGCGAAA
GCATTGAAGGTGAAAAAGGCTCCGGACCGGATGAAATACCGAATGTGGCATATAAAAGGGCCAACTCTAGCGT
ACCAGGATATGTTCCGGAAAGCGATGAAGAAATTCCTGGCCGAAGGTCACTTCCCAGATTTATGGAAGATCCA
GATGCTGGTGCTGCTACCGAAGCCAGGAAAACCACCCGGCGATCAATCATCATACATGCCTATATGCTTGCTG
GATACACTGGGCATACTCTTGGAACGGGTCATCCTTAGTAGGATGGCTAAATGTACGAAGAGAGATAACGGAC
TATCGGAAAGGCAATTCGGATTCTAGAAAGAAAAGTCGACGGTAGACGTAATTCGGACGGTCTTGGAGATGGC
CAAGAAAGCATCGAATCAAAAACGGAGAGGAAATTGTTACTGCGCCTTAGTCACTGGAGTTACCCATAAGCGT
AGAGATCGTCGGCTTTGCTGGTGATGTCGTCCTAACAGTAATAGGCGAAACACTCGAGAAAGTGTAAGTGCTT
GCCACGGAGGCAATGCGCATGGTCGAAGATTGGATGAATGGAGTAATGCTGAAGATAGCCCAAAGGTGCTACT
AGTTAGCAATTGCAAAGTGGTTCAACACGTTGAGATTGTCGTCGGTGGACATGGCATAGCATCACAACAGTCG
CTCAGACACCTGGACGTGAAGATAGACGATGGGCTAAACTTCAACAACCAAAGCACTTACACGGATCATGCCG
AACGCTCATAGTCCGAGAAGCAGTAGGAGGCGTCTTCTGGCTAGCGTATCATTGTCGATACTGAGATACGGAG
TTCCGGCCTGGGGTGCAGCACTGCAAACCAAGCGCAATCAGGATGAGCTCAACAACACGTTTCGGCTCATGGC
CATGAGAGTAGCAAGCGCATATAGAACAATATCATCGGAGGCAGTATGTGTGATCACCGGGATGAACCGGGAG
AAGACATCGAGTGCTATCAGTGGAGAGGCACTAGGCAGTTGAGAAAATTTACGAGGATCGGCTCGCTGGTCAA
GTGGCAGCAAGAATGGGATGTCTCTGAGAAAGGTAGTTGGACCCACAGACTCATCCCGAATGTTGGTTGGTTG
GTTTGACTTTATTAACGAGATTTTTAGCCCTAGGCTAGTTCATCTCGGGACCAACGGCTTTACTTCCTTTCCG
AAGGAAGTCATCACTATAACTTTTTACGTCATAAGTGACTATGTCGGGGATGGGATTCGATCCCAGGTCCTCG
GCGTGAGAGGCGAGTGTTCATCCCGAATGTGTCGACCTGGGTAAACAAGGAACATGGCGAAGAGAACTTCCAC
CTGACACAGTTCCTGTCAGGTCACGGATGCTTCAAACAGTATCTGCATCGGTCCGGCCACGCGCTGTCACCGT

TATGTCCCAAGTGTAATGATAGAGAGGAAACGCCAGAACACGTTGTCTTCGATTGCCCGAGGTTCAACATGGA
ACAAAGCGCGACGGCGACAGGACTTCAGTGCAGACAGAATGGTAAGCGATCCTAACCTTCGGAATCTAGCGAA
CAATATGGTGGTGGAGATAACGTCTATCTTACAGAGAAACTGGCGGGAAGAGCAGCAAACCGAGAGGCAGAGA
ACAGTGGCTGGCATCTGGTCGTCGGGTCGCTAAGGAACCGGAAGTCAACCCCCATCCGGAATCGTTTGATCGA
CCTCGGCACTCGAGTCAGCCTGACGAAGAAAGAAGACGAAGATCTCCCCTGCGATAGCTGCCGGTAGTCGGGG
CACCATGAGCTCAGACGTCTCCCTGACGCTTGGTGACGCCGGGGGAAGTCAGGAGGAGAAGGAAGCGAGAAGG
GAAGAAGAGGGTAAACGAAGGAAGGAGACAGAGATGTGGAGGAAGATGAAAAGGCAGCCTGCTCAACATGCAA
GAGCAAATCACTGGCAGACTGCGCAGAGTGCAAGAATACAGAGCGGGAAAACGTCAATAGCGTAATAGAAGTG
CAGATTCAGAAGTCGCATCTAGTAGAATCCGGGAGGATCCAGGTTACCGCCGAAAATGAGGACTAGGGTTTAG
TGGAGGGGCACGCAAGTGAATCCCACACAGTGTCAATTCTCTACAGGGCAGGTTTTTGAAACTTTTTGATACC
CTACTAGGGTTTAGTGGCATCAAGATCTAGAAGAAACTCAATCGTGGTATTCTCTCCCAACCTTCCTGAAACA
GACGATTGAAGTCTTTTCGAAACGTTGGAGCATTCCCTCTCAATAGGTACTGAGATTGAAAGCAGAAAAAATC
ATCTACTCGATCAGGTTAGTGTCGTAGCTCACGCCTACTCCGATCGCTAGGAAATTTCACTCTCAATTTCGTC
TCGCATCTCGCGCCCCGTCAAGTGCTAATTAGAAGTCAAACAAGAAGTGTCACTTTTTAAGCGTTCGATGCCC
ACCCAAACATTCCTGCAATATGTTGACGAACGACGACGGTTTCTCTACCTGCCAACCCGGATCGGTCACTTTC
CGAATTTTGGACAGCTGCTAAGGTGCTGTGTGACGATTAACTCTACTTGAAGTGGACATTGGTACCCCCTTCG
CTACGACCTGTTTCCACACAGCCAAACGGGAAGGGATCGCTAAAATCTAGACTGACACGCCAACCAACCTTTT
GAAATCTAAACTGCCCCGTGTGTGTATCTGAAGGCAAGTGTGTCTCTCCGCCCAAGATGCGAAGAATAATTGT
GCACGGGGCAAAAACTTTCACCGAAAGTATTGGAATATGATTAAGGTTTGTTTTGCTTTTTTTTACGATCATC
GTCCGCGTTAACTCGCTTGCAATAACGACGGCGATGATGACACATACCTACCTACATATCTACCTACGAAGCC
TTTGTCTTGAGAACGATTCGCGTTGTCTGCTGAATTGCTCTCGTCGGTTCGGGAAAGACTTGAATGCTCGAAC
TCGAGTGTGTGTGTTTGCGGACTGAACCTCGGTGAAAGTTTCTCAGTCTTCGGGGGATTTCAGCTCCTATCTC
ACAAGTGGAATCCAGAATGTGCTTGAGCAGGCTTTAAAGGACTTGTCTACCGTGGATGTGCGCGGTTTCTTCT
ACATACTCGCGGTGGAATTAGTCAACAGGCATCGAAAAAAAAAGAGTATGACCGGTTAACGGTACCAATAAGT
GGCTATTTCGAGTTGCCTTTTTTCAGCGGCTTTCGGAAGAATGTTTACCAGGGAAACCAGTTCCCAGCCAGTT
CGGTTGTGGGAACAATCGGATTCGATGGCGGTGGTGATAGGGGTAAGAGATGGAGGAACGTAGAAAAGAAAGT
GAAATGGATTATTTAGGTTACTCAGCAAAGTGCTTCTCACACGCAGAAAGCGGATGACACACCTGTATGCAAG
TTCCGTCGTCGTCGATGTCGTTGAGGTCGTTGAATGTGCATTTTGCCAGGTGGAAATGCAACGTATATTGAAT
GGGTGAGTGGATTGATTTCACAATCTGGTTTGAAGAAACGCAATTAGTAAAACTTTGTTTGAATATTAAATAG
CGCAATGAGAGGTCTGTTTCTTTTTGCCTAAATCTCAGTTGTTCATGGACCGATTTTAATAAAATTTTCACAG
CACGTCAGACATTTTTGAAGATTTGACTAGAGAAAAGTTTTTGACAAAAAATATAAGCAGTTTATCTCAAATT
AGGAAAGCCATACACAAAAACAGGCTTCTCGGAATCTACAATTTTACCAGGTCCGTCCTACTCGGGTTCAGAT
TGGGCACCACTTCTAGTACTGCATTTGGTCCCTTGGTACTGCAAAAGGTACCCAAGTTTGACAGATCGCAGTA
CATTTTACACGGCATTCGAGATTTTGTTCTATTACCTTATGTGCCATAAAATTTTGGGCAACTGCAAGGGGCA
TGGAGGTCTTTGGAATAGCTGCTTCTTATTAAATTATGGTGTGGAACTTATGAACTCATGACTTCTGTTTTTA
AAGCAATCAATTCGATAAACTACATTAAGGAGGAGGATCTGGAAAAACTTCTTGAAGACTACAAATTACCTAT
GAGAAAAATTAAAACCAAATCCTGCAAAAGTCTCTACATGATATCGTAGACAATTTCCGGGGGAATTTCTGTC
TGATTCTTTGCAGGTATACTTGCGAGATGCCTGGAAAAAATTTAGGAGTCATTCCAATAGAACATGTATTTGAA
ATCTATTTTAGGGAATTCTTAGATAAACTAAACGTGACTGGACTGCCTTTTAATGGGACTTTTACCAATCGAA
TCTTAAGGCGTTTTCCATTCCGGAAATTTTTAAGGCGCTAATGGAGCCATTATTCAGATATAAAAACAAAACC
GAGGCACGTTTCGAACTCCACTTTGATTACTATGAAAGGAAAAGGATGTAGATGACCTTCAAAGTTTAACGTT
TATGCAAAAAGGTTGTGTGTAAAGATCGTTTTGCATGAAATTCGTAAATATGTTTATGAGGTCACTCGTCCCC
CTCTAATGGAAATCCTGGCTACGCCCATGAGTATATTCAAATGTTCTCAGTGACACATTGCTTTTATATGCCG
CAAAATCTTCCACAATTAACTGGTTTTCTATGGGCTCAAGCGCTATAAGGCATTACGGAGCCCATTTCACCAT
GTAACGACGTAGAAAGCAGCAATACAAATGTTTGATTTCATCAGATTGGAAAAGTTTGTGTAGATCCCAAAGT
ATGATTTTTTTTACACAGAACTCTTCAGTTTGTGTAGAATTCAAACCATGATATGGTAGATAGACCAGCAGGT
AATCAACAGGGCCACAGAACAATTTACGAAATTGGAGATTGGAAGACCACTACCTCGAGCTCCAATTCATAGA
ATGGCATTATTTCGAATTCTATTATTACGGGACAATGTCGTTCAAGGTATTCAAGACAATAACATTCAGCGTA
ATGGTGCGTCAGGGTGATGGCAATCGGGAATCTGCTTTATAGAGTTTTGATTTGAGTATCGATTTTTAAGGAA
GACATGTAAAATTAAGCTCTGCTAGTGTTATGGCTTTGAAATCTATATAAAAAATAATAACGTATATTCCTTA
TTTTTTTCAAGCGTATTTCATGTATTGAGACTTCCTCGTAAAGCTAACTCGTGAAGGCTTGAGATTGGGATTG
ATTACAGTTCTTTTGCTATTGTATTTTTCCAGGACTGTGTTTGTGTGATAGAAAAGAACGGGAAACCCACAGG
TAAAACCGTAGATTTTTGAAAAAAATAAGTGTAGTAATGATTGCTACAACTGATCACAAAAGTATTGACAGTG
GTCTAGAGTGCCATGGTTTAATCAAAAGTGCATTTCTGAGCAATCGAAGTTAAAGTTCTGCGGCACTGCAAAG

TCAACCGTAAACCTCGAAAATTGTCTTACGTAATTAATGGAGCGCCCCTAAGATGTAAACATGTTCCAATCAT
CTGGAAATTTTTTGAAAATTTGTATGGAAAAAGAGTTAGGAAACTTCACAGCCCATTTTTTCAATGCCTACTT
TTTCAGATGGGACTGACTTGCGATTCACGGCAGTACGGACCTGAGAAGTAGGAGTCCGTTTGGTCGAAATATT
CAAGGGTTCTGTGTTTGAATTCCGTTGATCCAGGATCTTTTCTAAACGGAAATTTTCTCTACTTCCTGGGGCA
TAGCGTATCTTCGTGCTTGTCATACGATATTCAAGTGCAAAAAATGGTCATTTCACAAAGAAAACTCTCGGTTA
ATAACTGTGGGAGTGTTCAATATAATAGTATAAGTCTGAGAAGCAGGCTCTCTTCCAGTTCGAATTTAAAACC
AGAAAAAAAAAGAAGAAGTATGGAATGGATGGATCGAAAATGCCAAGAATATGAGTTTTTTTTTACCAATTTT
CGGGTTCTTACAGATAAGTTTGCGACGTTTTTTCTTAGATATTTATCAGCAGCTTGTCTCCCAGAACTCTCTT
AATGTGGATATTAAATCTTCGAGGTCTTAATCCTCAACATAACTCAAATAAAATGGCATTACGATGGTCAAAT
ATGACCAAACGACAAAAACCATCAAAAATGATAGAATACAATAAAAAAATACCAGAAACAATCGAAATATGAT
AAGAAACGGCCTGCCAATGGTCCAAAATTGACAAAAATGATGAAAATGTGACCGTTGTTAAAAAACATTAAAA
CCCATCAAACGACGGCTAAAAGCTTTCAAACAATGAATATAATCGATCAAGTTGATTGAAAACAGCCAAAAAC
TGCCTAAAACGATAGAAATATGATAAAAAAGGGCTTGGCCATGGTCAAAAACTGACAAAATGACGAAAATATG
ATCGAAAAATGGAACCCATTGAACGTCGGCCATAAACTATCAAACAACGGTCAAAATCAAACAATCATCGGTT
GATCAAACAACGGTTGATCGAAAACGGTCAAAAATTACCTAAAACGAAAAAAATATGATGAAAATGGGCTTGG
CAATGGTCAAAAACTGATAAAAATGATGAAAAACGGACAAGAATATCCCGAAACTATGAAGTATTGTTAAAAA
CACATTTAAATCCATCAAATGGCAGCCAAAAACTATCAAATGATACCCATAAACTATCAAACAACAGTTGAAA
TAGATCAAATTGATCGAAAACGGCCAAAAACTACATAAAACGATGAAGATATGATCAAAAACAGACATAACCA
TCCAAAAAATTATAAAACGTTGTTAAAAACGGATAAAAACTGTCCAACAATGGTCAAAATCGATTAAGTTGAC
CGAACACGGTCCAAAATTACCTAAAACGATAAAAATATGGCTTGGCAATGGTCAAAAACTGACACTTATGATG
ATAAACGGACAAGAACATCCCGAAACTATGAAGCGTTGTTAAAAAAACATTATACCCATCACAACGGCCAAAA
GCCATCAAACAACGGTCAAAATCGATCAAGTTGATCGAAAATGGCCAAAAACTACAAAAAGACTAGCAATGGT
CAAAAAACCCAAAAACTATAAAAAAAGCTACCGTAAATTCGGGTGAAATTGATCAGTAGGGTAAAATTGATCA
CTGTGTCACTCGATTTTATTTCTTCCTAATGGAGCACAAATATCAATTTAACCTGCAGTGAATGAACGTTGTT
TGTCGTAAGTATGTCCAAATTGTGTATTGTGAAGTTTTTTGCGTTCAAAAATGTTTATTTATATGAAAATAAT
GTAAAATTTCAAAATCTTGTACGGTGCATAGTTGACAAATATCAATAAACTTCTAGTTCTAGATAATGATTTG
GACATGCTATACTGCCGTGAATCGCAAGTCAGTCCCATCTGCATTTTCGTCAAAATTGAGTTGAGTTCAGTTT
TCAACATATCTTCCAATGCTCTTTCAGCTGCCCTATTGAGATAATAAGATTTGTTCGGAAAAAGTAGGAAAAA
ACAGCAAGTCAAATTGTCCCATAATGAAACGTAACCGCATATCAGTCCAACAACAGATTTCCGCACCGTGTAA
CACAAAAATGAACCGTGTTATGATCATTTTTATATTTTTCTCAGAAAGATATCGTAGTGAAAAGCAAAATGTG
AAAGTTTCATTAAAATTTGAACATATTCCAATCCTCTGGAATTTTTTGAATGTTCATATGGAAAACGAGTTTG
GAAACTTCACACCCCATTTTCTCAATGCCTACTTTTTACAGATGGGACTGACTTGCGATTCACGGCAGTATAA
TCCTGAAAGTTTGTGAGGATACTAACGATATATCCCCAAACTAGATTTCATAACCAAAACTCGTACCAATTCG
TTGATTTGGTTGAAATAATTGCATTTCTGTAATATATCAAGAAATTCCTAAGGAAATTGCATACATTTAGGCG
TTTTCTGCGTTATTCTTGAAATTAAACATTCATTATTTCTATAAATATTGTATTGTTAAGAATTTGGCAAGCA
TATTCGGATTCAGGGAGCTCAAATTTATTATGTAGACTTGTTTTGAAAACTAACAATAATGGCATTGATAAGT
GATCAATTTCACCCCGAAATGAGATCTCCCGATTTATTATTTTAGACATATTTTTTAGCACTAAAATTACATT
TGTTAGAAAATTTCGGCACTTGAGTCAATGAGGCTCACCTTCGTACTTGTTTTCCTGCATTCAGTTGTTTGGC
ATTGTCAACATTATAGAAATCAGACAAGAAAAACCTTGAAAAGTGATCAATTTCACCCGAAATTACGGTAATC
AAAACGATTAAAATCTAATCAAATTCAGCCTGGAAACAGTCAAGAGTTTCCAAAAGTCAGCAAGCGTGGTCGT
AAACATTAAAAACACCCAAAACGAATGACATATGATAATTAATGGCATGAAGATGGTCAAACACTACCAAAAA
TGATCGAAAACAATAAAAATCGATTGAATACAATAACAAAAAATCTACCAACAACTATCAAAACGTCATAAGA
GACGGCCTAACATTAAAACCTTTCAAATGGCCAAAAACTGTCAAACGACGGTCAGAATCGATCAAAAACAGGC
TTCCGAATTCTCACTCACTCTCACGATAATGGATTTATCCCTCACAGTAATTTTCAGCGATAAGCTGCCTTGC
GATTTTATCCGTCCACAAAACAAAGCGAAAATAGATAATAGGGCCCTAAAGCCCTGTCCCAATTTTGAAACCA
AACGCTTAAGTTTAGGTCAAAAACACATGTTTACTCAATTTATAAATGGTTTTCGTTGGTTAAAGTCAATAAA
ACATTTTTTTATTGTTTTTGAGATTTTGTCACACCCCTTGGTTTAAACTCAAATTTATGGGTATGTTTTGTTG
TCCGTGTCCCTTACGAAATGTCAGATAGGAACAACCCCAGTGTTAAAACTAAAAGCCCTGTGGTATTTTTGTC
GACAAAGTGAACGTCAAACATGATCAAAAGTGTCAAGATTCATATTTGGAACAATTTTTGAAATTAAATTTTA
AATATGTTATAGCCGTTATTTGAGCCGGAAAAATTTCTCAAGTAGTTTCAAAAACATGCTCTTTCGTATTATT
GAATAGAGTGATTCCAAGCAACAGCACCAAAATTTGGTAAATTTTTTAATTCATTTTTTTCTATTGAGCTGAA
ACTTTGCACAGTTTTCCAGTTCCATCTAAATCGTCATTTTCCGATATCAAATCTTCAAGTTGAGTCACGACTA
ACTTTTCAAATGGGTGTATGTGAAAATGGTTCAAAAATATTCAAAAAGCTGCACAGCAAAAACGGTTCGTTCG
ATTGTTAGACACCTAAAGAAACAAAGTTAGACAACTAAATAAAGATTCCAAAAAAAATACACACAGTAAAAAA

AAATTTTTTTTTGCATTAAAAAACATAATTTTTGACACAAAAACTCAAATATCTCAAAACCCTATCGGAATAC
CAACGTAATTTTTTGAAGGAAAACGGTCCATTATATTAGCTATCTACCATAAAAATTTGGTGATGGTAAACCA
ATAAACAAAAAAGTTATGACATTTCAAACATGTCACATTTTTCACATTTAGTAGAAAAAAAAAATTTGTTTCGG
TGTAAATTATTACGGGAACCGCAGTTTGTTGCTGATTTTATTGTTAAGGGCCTTGCGTGAATTAAACAAGTCG
TTTTCATGTATTCATTAGTATTATGTATATTATATGTATAAATATTATGTATATGTATAAATATTATATGTAT
ATGTATAAATTAAAATGAATTAACAGATTACACGAAAATAATTTTTTTTTTACCAGGATATTTTTTTTTAGAG
TATGATCGATGAGTTTCTAAATGTTATATATAAACTTTAAAAGTTTTGGATTTGGGTATGCGCTATGAGATCA
TGAAAACATTTTATTAATACTTATTTATTTATTTATTGTTATTCAATTTTTTTACAATATCGAACACTTTTGC
ATCATTATAAGTACAGTTCGAGTATAGTTTTGCTTTAATTTTATTTTCTGACAATGGAATGAAACAGTGAAAT
TTTTGGGTTCCTTGGATCGTTTTCGCGTTATTATATTGCTCGCTGAGCTCTGATGCCGTTAATTCGTACTCTT
CAGTAGAAGTAAAACAAAATGATAATTTTGTTAAATCTTCTTCTTTTCTGCGATTCGCCCAATCAAATAGTTC
TTTTGCAGTTTTAATTGGATGCTCACGTTCTTTGGCTAAACTTGCTCTTGTGGCCATGCGCTTTATGGTTCCT
CCAATAGCATCACAAGGACCTTTGCCATGTGACGTAGCAAAGAAATGCCATTCTGCATCAATTCCGTACTTTG
ATTTAAATTGACATAGGCTCGAAAAATTCTTACGGTTTTTGTACTGCGATGCTGCTCCATCAGACATGAAATA
TATCTTTCTGATTTCTTTATCCTTATCAACGCGTAAAAAGTTAATCATTTTGGCAATGAACAAATTTACAGAT
ACTGAGTCGTGTCTTAAATCTTCGGAAATTACAATAAAACTAAAATGTTCAATTTGCGTACATCAATTGAAAT
AAATAACGAATGGATGAATTGTAGCTTGTTGGAACGATGAGACTGCACTTCATCTTGCTATACAAAGCTATAG
TTTTCAGAAAAATCACAAATGACTAAAAATTCACCATCTTGTAATGTATTTTTCGTATTTTTTAAAAAGCGGG
ATTGCTCTGTTTTAATAAAGTCGTGAGGAATTGAACTTTCTAATTTCAAGCAAAAAAATGACACAAACTCATC
TACAGGTTTTACAATAGTTTCTAGGTCACACCTATCCGTGGTCACCCATTGCTCAAATGATAACTGATCAATA
TAATTTTCTTCAAACTCAGCGAATAAAGTATTTTCCAATGATGAAGAATCTGGACAATCCGAACAAGATCGTA
GATAGCAATTTGATGTTGTATTTTCACACAAAAGACTACCAGTTAACATTTTAATATCCTTTGATAAATTGAT
TCTTTTCAAACTATGTAAGATTAGGTTAATATTTTCGTGTGTTGTGCACACACAAACATTATGTGTTCCTGAA
TTGGATAGAAGCTTGCATTGCCTTGGACGAAGGCTTGCAAATGAGGAAAAACCTACCTTAATATTTTCATTAA
TTTCCTTGAAGCGTGTATACTCTTCTTTCAAAGTAGTCATCATTCATCGTTTTTGGATTGCTTGACGCTTTCC
ATCTTTTTTTACAGATACATAATCTTTTTGGCCAGGCATAGCTCTACTTACTTCATCGTCTTCAAAATATTGA
ATTATTTTTTCTTTTGTCTCATCTGTTAATGAAGTACTCGACCTAGCATTTTTGGTTGCAAGACAGTTATTTT
TGAATTGTTTTGCCTCTTTTGCTGTATTTCTATTGGTTTTGAACTCATCAATGGCGTCTTGAATAGACCACGA
GCTTGGCAGCATCGACAAAATCAATAATTTTCCTTTCCTTGTCGTGGCTAGATTCGAGAACCTTTCCTTCATA
TTCATAATTACCTCATCGTAGTCTGTATTTTCCACATCCTCAGGTCCTAATTTGAAGAGGTTTCTTCGTACAG
CTTCGTTGATTTCACGGTATTTTTTCTCGGGATAATTGACGTAACCCATCTTCGTCCATTTAATCGGAGTCAC
TTTTATTCCAGCTATCCCTTCGTTGAAGCGTTCGATGTTGACCTTCTGGATGCACTCATCTTCTGATTGATTT
GTTGAAACAGATGTCGCTGATGGTACCGTGGCAAGACTATCTGCACTTGGTACTTCTGGTAACTCCTCAGTTG
TTGTCGGTGCATCTAGTAATTCCTCAGTTGTTGTTGTTTTCGAACTTCCTGCAACCTGATCCACCGATGATGT
ACAGATTACCCGTTTGTCAACGTTTAAACGGCAGGACGTACAAATGCGTAAATTTGTATTCAATGTAGACATT
GGAGCATAACCAGCCGCTTTCAGTTTATCTATGGTGCTTTCGGTGAGATTTCGTAGCTCTTTCGAACACTTTT
TTTCTGCAAACGGCCTGCAACAGTTGAGAAAGCGACTACTCATGTTGCTCGTTAGATTTTAATAAACAAAATC
ACTTTTAAGTTTTTACTGACTAGTTTGGTGTCGTTTGCTTGACTGAAGAAAAATTTTACAATTAAATTTTTTA
TAACCATAGTGGTAGTATATTTTTAGCTTTTTCGTGAGTATGTTCATGGTATGTACCTATCATGTTTTTGATG
TTGTTGAAGTTACTCGCTTTCTCCCAAATATGATTAACAAAGTCTATTCTCTACCAAGCCGGGTCTATACCCA
GGTGTAATCAGATTTTGACTTTGTGGAGAAAACCAGCGCCGAGAAAACCGACCTGCTTTACGTATACTAGATC
ACCTGGGTATAGACCCGCCTTGTTCTCTACGAGGAAAACTTTTTGCAGGTAAACTAGTCGTATACCTGTATAG
AAAACTTTTTTTTTAGCTTTTGTTTTCAATATTAGATTTCTTATAGGTTTCTTTTATCAAAAGGTTTCAACACT
ATTGAGAGAATTTTTCTTCAGTTACGTACAATAAAAAATATGACACTATCAAGACTTTAGATCACAACACTGG
ATCGCGTCTAACTTTCTAATAGATGCTATAATAGTTATGAATAATTAAATAAAATATCATGAAAACAACTTGT
TAACCTCATGTGGTACCCTTAACAAAAAATTCAGCAACAAACTGCGGTCCTAAAAAAATTAACAATAAAAAAA
AAATCACTAAGTGTCAAATTCGTGAAATATTTGAAATGTCATAACTTTTTTGTTTATTGGCTTACCATCACCA
AATTTTTATGGTAGATAGTTAATATAATGGACCGTTTTCCCTCAAAAAATTACGTTGGTATTCCGATAGGGTT
TTGAGATATTTGAGTTTTTGTGACAAAAATTATGTTCTTTAATGCAAAAAAAAAAAAAAAATTATTGTGTGTAT
TTTTTTTTTTTGGAATCTTTATTTAGTTGTCTAACTTTGTTTCTTTAGTTGTCTAATAGTCGAACGAACCGTT
TTTGCTGTGCAGCTTTTTGAATATTTTTGAACCATTTTCACATACACCCTTTTGAAAAGTTAGTCGTGACTCA
ACTTGAAGATTTGATATCGGAAAATGACGATTTAGATGGAACTGAAAAACTGTGCAAAGTTTCAGATATTTTT
GAAATGGTCGATCAGAATCGACTTGCATGCCTCCGTGGAATCCCTCAATAGAAACAAGAATTCATTAAACATT
TTAGGACCCAATTGTACATTTCCGCAGCTGTGAGAAGTTTGTTTTCTCTTTCTCCGATCCATGGAGAACTTTT

CCTGTATCCATCGCCACGAGCAGCAGTTGCTTTTATGCGCCAAATTAACCGCTCCTTTCGTCAAGTTGGTGTG
GTAGCATGGTTAACGTGCACGCGTCGCGGTTGTTTTTAGCAATGTTCTAGGTTCGAATCCCGTCGCCGGCACT
AGTTTTTGTTTATCCACATAGTGATAATTCTCGGGAGCAGTTGGTGAGCGAAAATATGATGGAGTGAAAAATA
AATTATCCCCAGAGTGGAATGATTTTCGGTTATCCTCCATCGCCGCTCCAGGGAAAATAAGTGTGAGCGAAAA
AAGATGTTCACGTGAGGAAGCAAAAAAAACATTCTCCTTACGTACGAAAAATCTTAGATTTTGCATCATTGAT
ACGAAAATTCACCCTGATCAAAAACGATCCAAAATGGTCGAAAACTATCCAAAATAGGACCAATAACGACTCA
CCAAGAGAACTGTAAAGACCCATAAAGCGACGGTCAAAACATCTAAAAACTACCCAACATAACTGATAAATGA
TTATAAGCATCCTGCTGATTGTCAAAAAAATTGAAATCGATCGATCATAATAAAAAACAATCGAAACAATCAG
GAATGGCCACAAACTTTCAGAAACATATAAAAATGGTCGATAACGATCAAAATCGATGGAAAAGCCTTCCAAA
ACGAATAAAATTAATTAAAAATAAAAAAAAAACCGAAACAATTAAAAACGAGCAAAACCAGTAAAATTCTATG
GAAAACTGTTCAAATACGATTGTAAACATCCAAACACTATAAAATATAATCCAAATCACTAAATAACTATCGA
AAACATAAATATGGTAAAAAAACGAACAAAAGCACATCTTAAAATTATCAAATGGTGGTCAAAATCAAACAAA
AGCTGTTCTAACACGATAAGTAACAATTAAAAAACGATCAAAAACAACGAAAAACAATCGAAAATCATTGATG
CTGGCGATGCAAAACTATTGGGAACGTTCAAAAACTTTCAAGAAGCGATCGAAAGATAAACAAAACTCTTTAT
AAACGATTCCAGTCGGTCAGTACGGTCGGAAACATCGAAAAACAATCAAAAACTATTCAAAACAATCTATACA
TGATCTTAAACGGCCTGACGATAGACAAAAAGATCCAAAAACGATTACAACCGGCCAATAATAATCAAAAACA
ATCAAATCTATAAGAAACTTTCAAATTCTCAACGTGATCAGTGATAGATAGTCCGAGGTACCTGTCAAGTTTT
TACTATGATCCTTGTTGCATTCTAAAAAAAATGTGTGTCGGATTTCTTGGCATATTCTCTCTAAAACAACGGT
GGTTTTCTGTTGAGGATTTTCTTAGATTTTTTGAAATCTTTGTCCAGAATTAATAAGCAAACCTGACTGATTT
TTAGATATGACCATTTATAAGTTTCTGACTTATGTTGAAATCTCCCAATGCACACTGGGCCAGAATTTAGCCA
GACAAAACCTCAAGCGCTTTAGGAGTGCATTCTTTCAGGTTGTTGTCTTCGGAGACTTGCCCCGGATTTTTTT
TCTTTAATTTGATGATAAAAGTTAGTTGGAAATTTCACCGCATAAGTGGCGCTGCGATGTAAACCTTTTTTAA
ACCGCGTCTTCGGCAAAGTTTCAGATCGTATAAAAATACGACAAGTTGTACAAGACACCTTTATGCATGGCCG
TGTAAAAAAAATCTTTAAAAACAAAATTGGGTGTAGTACAAAAAAACCTAAAATTTTAGTAAATTTCACAATT
CTTACCATAATGTTATTTGAAGTACTAGAACATTGATGTTTTCGACAACTTGTCGTATTTTTACACCATCTAA
AACTTTACCGAAGACGCGAAAGCTTTAGGACGCGAAAAAAAAAGTTTGCATCGCACCTATGCGGCAATTTTTT
TAAATAATATATCATCAAATTAAAGAACAAATAAATCCAAGATAAGTCTCGAAAGACATCAACCTGAAAAAAG
CACTCCGAAAGCGCTAGAGGTTTTGTCTGGCTAAATTATGTCCCTGGACCAGTGTGCAATGTTTTGTGAGATT
GGCTTGAAACTTACCAAGAATCTCTTTTAAAAGATTCCTGAAGATGTAACTTGAACAAAATTACAAGGCGATT
TCCGACAGATCGCTTTGTCTATTCCAACATATTTTCAAAAATGTTTTTCCGTAATTGATCCCTGGGTATAAAC
CTATTTTATAAACAATCTGTAATCTTCCTTCAATCCTTCCTCAAAGAGTGTAGTTGAGGGTCTAGGAAGATTT
CTAATTAGAGAATATCAAGAGAATCAATCAAGAACCTTTCCAGTTATGAACTAAGGATCTATTTAATGAACTG
CCAGAGATCTTGTGAAACGCAAACATTTAACGAGAAAAGCAAATGATTTAACTTTGTTATGGTCAATGGTGCT
ATGACAAGTGAAAAGAAATTACGATTGAAAAACTCTGTCAGAGCAACGTCGAGTAACCCAAATCTGTTCCCGT
AATAGTAGTTTACGGAACAAGTTGCAGAATGATGATTTTTACAGCACGAAGACCACTTACGGAACTCAAAACA
GTTGCGTAATGAGAAAGCGTTGCGTAATGAATCATTACAGCACTGGTTTCAGTTAGGTAATGACTATTCCCGC
ACTGCATACTTCAGTGCAGGAAAGTAGGCCGTTTCATGGCAGATTGGCGTGATGAAAACCAGCCTATTACGAT
GAGAAATTGCAAAAAGAATCTTTTAACTTCACTCCGGAAAGAAAGCAAGAAGTTAACCCGGATTTGACCCGTA
TTCAGTTACTTTGATCTCGATGGCGAGCGAACGTGGGTAGTCCATCTCCAGCAACTTCCCCGGAGAGTTGGGT
GACTCAACAAAGTATCTACCGTCGAGTTTCCAAACCAGCTGAGAGAACCCAACTGGAAAAGGCAACTAACCTC
TTTGATTGGTTGATGCGCGCAGTTTTCACCACTGGCACCGCTTAGAGTACTAACCTAAGCTGACTAACAGTAG
TGGTTGTGAAGTTATCAACCTTTTTCATCGAGGGAACCATCATCATCCCTTTACGGGTGGTCAGGGCCAGGAG
GGCAGGTGAGCCGCAAGGGGAAACCATTCCGTGAAGTCGATTTCTCCTCGCATCAGGTTCGTCGCCGAGCACC
GATTCGATTGGTGGTAGGGAGGAACTTCTTTCTTCTTTTGTTGCGCAGAAGATCTAGGCTGAGTGCGCGGTTG
GGGTCGCGGTAGCAAGAGACGGGGACTCAAAGCGTTCAAAACTTGAGAGTGCTTTCCTCGCTAGATATCGCTC
GGCGATTGCTGCGATTTCTTCCATTGGCTCGTTCGCTTGCTCTGAGTACCCGATGGGGAACTCAGCAGAGAAC
GAACGATATCTCTGAGTCTCTAAAGCTACGCGGACGGATGAGCGCGAGGTGCTTAGTAAAGAAGACAGTGAGT
GACAACTGGAAAGTGCTTAGTGCTTACGCGCATGTGGAAGATGTGCGCGCTGCGAGTGTGAGTGAGTGACGTT
CGGTAGGGACATACGAGTTGAAAACATACAGCAACTGTGCAATAAAAAAAAACTATTCAAGAAAGAAAGCTGG
TGGAGAAATTTTCTGTTTTCTCCCCTGGCCGCGCGCTGCTTACTGCTTGATGGCGATGACTCGCAAGAAGTGC
TGCTTTAGCTTTCAAGTGGAAAGAATTTGTAGAAATTCCACCCATCGTGGTGGATTTGTGAAAGATACCCTTC
TTTGGTTACACTTACAGGTTTTGTTTTGACTTTTGAGTCACAATTGCGGTACATTTTGAGATATTATTTAGTT
TCGTTTGAAGTTAGAATCGAAATTCAAATATTTATTACAACGTCCCAAGCAACCAGAACATCGGCTTTTACTT
AAGCGATGAACTCAAAAGTTCATTTCTAGTAGGTTTTGAGTTGCAATGATACTACATCACTGAATAAGAAATC

AACGAATACTAACAGAACTTGATTCTCAAAAAAGGAACTCATGCATTTATCAACAACTAGAAAGTTCAGAAAA
AAGGTCGAGCTGATTTTTTTCCCTATTTTACGGTAAACATCATTCTATTTGAGCAACTTGAATGAACTTTATG
TGAACTGAACTGCCATGAAACGCAAGTCAGTCCCATCTGCATTTTCGTCAAAATTGAGTTGAGTTCAGTTTTC
AACATATTTTCCAATGCTCTTCCCAACAAAAAAAGCAATAATTTATCGAGAGTTGATCCAATTCCGTCAGGTT
GCCAAACTGCATAGAAGTTCCTCCGTCGCCCGTCAGTTCTACCAACCCGTCTCCCCTCAACTGATAAGCCAGA
CGAAGTGTATACGACCTCCCGAAAGAACACGGTTGTTTACAAGAGTGTAAATATGTCCACGTAGAAATACGTG
GTGTCCGCGCCGCGTTGTTGTTCACAGTTGCTGACAGGGAACAAGTGTTCTGCTGCTGCTTCTGCTACTGCCG
ACTATACTCCGAGACTCTGTCTTGGAAATCTAATCTCCAGGCCCAGCGACGATGACTGTGTTTCGCCACCCTC
TCATCCCCTGGGAAAGGAGTAAGGAGGGGGGGGGGGGGCGGAGAATGGCATCGTGAGCTATTTCACTTATCAA
GCCATGGGCATCGACTGCGATTCCCTGGGCCGATGGTGATGTGTGGAGGCACAGACAAACGGACGGAATACTT
GAAATTCTCATCGAGAACGCTTTAAACGATCATTTCAAATTTCTTAGGTTCCATATCTCACAACCAGAGGGCG
TGCATCGCTTTCTTTTGTGTTTGACGTTTAACACTAGTACCTCCTACACGGTGAAAACGGTAAACCCACATTT
GAGTATTTTTAAACTTATTTTGAATTATTTTTCTCTCCCTATTTCATTTGCTCCTTCTGTTGTTGTCAGAGAG
CGAAGAGCAAAACAACTCAAAAACCTAACCTTAGTGCGTTACCTCAAATTTGAGTATGTGGCACAGTACTGGA
AGTTGAGTTATTTGATCTGCCGATTGAGCGAAAAGAACTTAGCCAGTAGGTATTTTTTCACTTGGCTGCAAAT
GAAAACAACTCCTACCCTATCAACTCAAAATGAGGTTAACGCACGAACCCCCGGAATTCAGTGGGATGAACTC
ACTTTTGAGTACTTCATTTTCTCCGTGTACCTAATCGTATTTCGTGCAATATTCATGAGGATTGTGTGAAGTC
GGCGATGGATTTTTAAGGAAATCGTTACGGCCGTTTGTCTGTGGTACAAGCTTGACGCGACTCGGACGGGACC
ACATGACTACAAGTTTATTCTAGCCAAATATGAAATCAAAAACACCTTGCCTCGCATTGATTGAGTAGGAGAA
GCCACGAGGGGGATGAAGCAATATGGGGCATCCGGTGGATGTTGAGAGCGGAGAATGCGGCGGAGGACGACAG
GGTGAGGGAACAATATCAACGGCCGCTCAACTGATAAGGAACACAGATAAGTCCCCCCACATCGGGAGAGTGA
ATGAACAGGGCGCAGAACACACGGCTCCCTGAGTTAGCCGTAGCCTACTTTGTTACGGTGTGGGTTCTGGGAG
TTCTGTCGAATCGCAGTTTTCTCAGCTGGCTGCGAAGGCGATGGTGATTTTTAGTGGAATGATACTTACGTTG
CATCACGGCGGCGAGCGAAGGAACGCCCTACGTTGGGATGGATGGGTGAACGATCCTCCGACTATCAAAAGGG
GGATCTTGTTGTTATCAAACGAATTGAGAGTGGGATTATAGAATGAGTCTTTGTGATGGCCTTATGTCGATTC
ACATATGGTATAACTCAGTCTAAAAATTTAGGGATGTTTATAGCAACGAACGACCGAAGAGATGAGTCAAAAC
GAATCATTGAATAGAACTCATTTGACAATCTTCTTCTTCTTCTTCTTTCTGGCGTTTCGTCCTAATCGGGACA
GAGCCTGCTGCTCAGCTTAGTGTTCTTATGAGCATTTCCATAGTTATTAATTGAGAGCTTTCTTTGCCAATTG
ACTGTTTTGCATGTGTATATCGTATGGCAGGTACGAATATACTCTGGGCCCTGGGAGTCGAGAAAATTTCCAA
CCCGAAGAGATCCTCGACCAGTGGGATTCGAACCCACGACCCTCAGCTGCTAAATAGCTGCGCGTTTACTGCT
ACGGCTACAAGTCAACACCCCGAATCATTTGACACTCACCGTATCCAAATCTTTATTGCATATAAAATATAAC
TTAACTCAGTTTGACAATACTCAAGCCATTTTGACTGTTTGCAAACTGACAAAAGAGAGGAAGATGACATTAT
TTGGTATCTTCGTCCTGCCTGTGGATGATCTGTCAAAATGACCTTAGTTTTGCCAAACTCGATTGATTGTGCA
ACAAAGAATTACAGTTCTATCAAACCCAAAACACTACGGTGAAGCTATCTATGCTCTTCCTGAAAAAATATAA
ACTAGTAGGAATTATTGTGAGATCCTTTTGAAATTGTTTGGAAATTTTTTGATGGATTCCAAATGAGATTCTT
AAAATAGTGCCAACTGGATTTTTTGAATAAGTAAAGGCTCAAACAGGATTCTGAGGATATTTTCGATGGATTA
TCAAAACTTATATCATAACTGTCAAGAATTCAGTGACTGTTCATAAGAAATCACGTCAAGACTGAGGTCAGAC
GCTTGAGATCTTTGAATGATTTATTTATATGTTCTTAATCTTCGACCTTCCAGGCCGCATAGACCGTTTTATA
ACGTATGCATCACACAACAATACTGCTGGGGGACGGACCTGGTGTAGTGGTTTGAACACACGCCTCTCACGCC
AAAGAACTGGGATCGAATTGGGATAGGTCCTCGAGATGGTCACTTATGACGTAAATTGTTATTGTGACGACTT
CTTTCGGAAGGGAAGTAAAGCCGTTGGTCCCGAGATGAACTAGCCTAGGGCTGAAAATCTCGTTAGTAAAGAT
AGAAAAAAAAAACAAAACAATACAGAAATCATGAATAAATGGCTATTAACTTAGAACATATGAACCAATGTTG
GAATTGGATAGAACGGTAATATAGTGAAAGATTATAAAAAAATATACCATGTATATTTTACTCAATCTTATAG
ATTCTGAACTTTTGATTTGTTCATTTTTCAATATATATTTTTTAATTTTAATCCTTGAATTTGTAAAAGGTAA
GCAGTAAGCGGCAATCCATCAAAAACATTGTTAGAAATAGACAAAAAATCTTACTACATAGAAGTCTTTAAAA
GGTTTTGCAAACCGTAAAGTTTGTGGTTGCTTAATTTTGGCAATTATTATTTGTATCAATGCTTTTATACAGA
TTACAATTTTACAAGGAACCAGTTACTTCTAGATTCTGATCAAAGTTACATTTCAGGATCTGGGAAGCAATTC
ATCAAGATCACCCAATTATCACTAGCAGTAGTGGCAATGGCGATGCATAACGGCGAATATCTCTAATTTAGTA
AAAGGTTTCCAACAATTCTGCTTGAAGTCGAAATATGACAATTTAATTCACTTTAAATTGCTAATAGCCTTTC
GAAAGTTGAGAGCATCAGTTAGTTTAATATTGTACACTTATTTTTTTTAAGTTACATATAAGTGCTTCGTTAA
AAAGATTTCTGACTTTGTAACTGCCAAGCTCTAAAATGAATCATGTAGAAAGTGACGATTCCCAATGGAACTT
CAGTTTATTTTTTTCAAAACATGAGATTGTACATACTAGATGTTTACAGGCCCTATGCCTTGTGGAGCACATT
TGGAATTGAATCCATAATTGGATGTAATACGAGAATCAACTCAAATTAACTTGTGTGTCGATGGCCCCGCTG
ACATCGCCGATTCATTTAACGTACATTTGATCTTTTTTTAAACATGCTTAAGGAAATATTTAGCTTCTGCTTT

ATGAATTCTTACAGATATTCTTCAAATGATTTCTCCAAAAAATGATGTTGTTCCTGGAATTTCTCCACGGATT
TCAACTACAATCATTCAGAGGATTCCTCCAGAGATTCCTCCAATAATTTCTCTAAGGACTCTTCTCAAACTCT
GCTGATATTCATGTATAAAACTTGAAACATATTCTTAAAATAATTCCGACAAACATCTCCCAGAAATTCCACT
ACTTAACCCACCTGATATTCATGTTCAAAAAAATCTGAGACTTTTTTCAGAAATGATGCCAGATGTTTTAAGC
GATTTCAGAGATTCCTTTCAAGGGTTTCCAGGATCATTTGATCAGTATTGACTTAACGGATTTCCCTTCTAAG
AATTCCTTGAGGAATTTCATCCGAAAGTTCTCATTAAGTTCCACCAAGATGTCCAAGAGCTGTTTACGAGAAA
TCCCGGATTCCAGGAATTATAAATTCAACGATTTTGAATTGCTATGATAGTTTTTTTTAAATATTTATGAATT
ATACTGTGCCTTTGATCAAAAATTTCACCAAGTAATTTCTACAGGATATAAAGTGGATTGAACTATGAATTCT
TCAAACCATTCCGCCTACGATATCTTAAAATCTGCAGTCTAAAGATTCATAAATTATTCAAGCAATTTTTCAT
AAATTCTTTAAGTGGCTCTTTGAAAGAACAATCCAGTTATAAATCTTGGAATGCTCTACAAATAATTCAAAAA
AATCGTACAGGTATTTGTCCAAAAACCTGAAAAACAATATTCTCAGAATTTTTCTACAGCAATTTTATTATGA
ATTTCCGCAGAACTTTCTCCAAATATTTATGCAAGAAGCAACTACTTTTTTTTCTTCAAGAATCCTTGTGGAA
CGATATTATTCATTGATTTTATCAGGAATTTGGAAAGAAAGTCCTATATGGTTCTTTTTCTATTGGATTTTTC
AAGTAATTCTTAAAGTTATCGTCTATAGATTTTTCTTTGGATTATTTTCATTATAAAAATAAATTCAGGGACT
CAACCAAAAATGAGCAATCGCACCACGTATCCGCTCAGAAAATTTTCCAAAGAAAATCTCTCAGGGATAGTTT
TTGTTTTAAAAAGACAATTTACACCGTATTCAGCCAAAGGCTGCACAGACTGAACGATCACTAACATTAGGCA
ACGGACAACACGAAACACACAGTAGCCCAGTGATGAGTTTTTCGTTTGACGAAAAGTTTCCATCGGTTGGAGC
GGGAATCCCCCACCTCGTGATACAATACGCCTAAGCGACTGACGCCGCTAACCGCACGGCCACGAAGCCCACA
ATTGGATTGTATTTAATGGAGTTTTCTAGTGATTTCTTCGTGAATTCAGCAAAAAAAAACATGTTTATCAAGA
ATTCTTGCGACATCCATTCTAAAATTCTGTCAAGAAATTACAAAAGGAATTGTTTTAAAAATGTCGAAAAAAA
GAATATGTAAGAAAGATTGAAAATGCCTGTAAGGTCAATAATAAATATTTAAGACCCAATTTTCCATCAAACA
ATATATGTTGAACGAAATTTAGATAAAAAAGTTAACCTCGATATAACGTAACGAAAATAGAAATCGAGTTACG
TTATATCGAGGTATTACTGTGTAACAAAATGTGTTATAAGCAATTGCTCTCGAATATTTTATAACAAATCGTG
TTATAATTATGTTTTGAATTGCAACAAATTTGAAGCTGCTATAGTTATTATAATTGATTTTGTTCCAATTATG
TCAAAGTGCTTCTCTCAAAAATGAAACAAAATTTGATATTTTTAACAAACCTTGATATAATTGTGCTACAATT
TTGCTGTAATCCACTGGTCGGGGTCAATAATATTGTTTTGTATCTTTTTTTTTTTATTTTCATCACATATCGAC
TTGATAATTTAGTAGCTTCCGTCAATGCATTGCAATTATTTGACCTCATATCAATTTAACACAACATTTCTGA
GAAATTTTATGTGTAATCGCTTCAGTACTTATCAGCGATGCATTATCAATCGCACTGCTTTGCATTCGAGTTC
ATTTCGTGCTAATAATCGATAGTCCATAATTGGTACACCTTCATGGCGAATACCAGGAACACTATTGCTATAT
TTCATACAAAAACAACGATTTTGAAAACCAATTTTAGATGCTTTAAGGCTCAAGAATCCATCATTGATGATAA
AATCATCAAAAACGAAAAACCAGTTTTTTTTGTTCAAATTTGAATAAATTCTCATAAAAATCTCTCATCGTAA
TCCATGTAGCAAGTGTAATGCAATGATGGATTTTCTTGAACATTGATTCAAATTTTGAAAGAAAAAACTGGTT
CGAAAAATTCGAACTGTTTATTTCATCTGTTTCGCAAGGCTACATACAAGTAACTAATAAATCAGTCTCACGT
TTTGAAAGCGCGGCCAAAATCTGATTAAAAAAAAGTTTATCTCTCGAAATAAATATCTATGAAAAGTTCTACA
TCTTCTACAAATATTATTGATTTTTGAAAATTTGAATACTTAAATTGTACATTGCACCATACTGGTTGAGCTG
AACGTGAAAACTTCACTTTACTTCACAATATTAGACGTATCGACCATTGGTTTGGTCCTTCTTCAGGGTGATT
ATAAGGAGTACAACTTTTATGTTGTCGCCTATTAAATTTTAACAGATCGAAAACCCATTCAACTAGAGATCGC
AGCTTAAATTTTACAACTAATAAAATGGGAATTGATTATTGGTTAATGTTATTGCATTCAAATATTTCGAGAA
ATTTTGAAAGTTTATTATCCCCAAGGAAAAATTTCAAAAAAATTGAAATTTATGTTCTGCATTTGGATTTTTT
TTTTAATTATTGAATTCCAATTCAATTTCAAAGCAGGGAAAGTCAATTTTGTTCAATAATCAAAATGTATATA
GATTTATTTATAAATTTGTTATAGGATTACGTTTCTCCAATGGATCCAAATAATGTTTTAAATATTTTGAATT
GGAATGCTCGTTCTTTGAATGGAAAAGTTTAATTTAACAAACATATAGCAGTTATAGCTGAAACTTATTTGAA
ACCTGGATTCTTCAGTCTTGTGCACATGAAAATTGAAAGTTTGGCTTCGTTTTATAATCACCTTAATCGACTC
TAGTCATCTTTGTAGCCAATTAGTTACTCATGCTGATTTTGATTCTGATCATGTCCCTGTTACATTTCAAATA
CCCCATGAAGTGAATCTCAATCCTATCAGCTCCACTTTCAATTATTTTTGAACCGACTGGAATATATAAGAAA
CATATATTGACTCTAATCTTGATGTTAACATTTCTTTACAAACAAAACTTGATATTGACAATGCTCTTGAAAT
TTGAATCCGTGATCATAGGCGATGATCTTAAACTCTTGATCCGCCTTAAAAACGTGAGGAGAAGTCAATTTCA
ACGCACTTGCGATCCTGCTATGAAAATTATATGGCAGGATTTGCAGAAAGAAATCAAGAAACGATCTGCACAA
TTAAGAAACAAAAATTTTGAAAATAAGATTTCTCAATTGGACCCTGGCTCTAAGCCCTTTTGGAAATTATCTA
AAATTTCGAAAAAGAAAACCTCAGAAGCCAATACCGGCATTGAAAGAGGAAAACATATTGTTACTAACTATTT
TCTAAACAGTTCAAAAATTTGCTATGCAGTTTGAAAGCGCGCATAATTTTCATTTGGGACTCAACTAAAAATC
AAATTACTCAGGATATCGAAAACATTCTCAATCAAGGTAATGTTTAAGAAAATTCCTGGATTTATTAGGAAAA
AGTGAGAACAAGCATAAAAATTCAAAAATATGAAAGCCCCTGGCGATGATAGAATTTACTACACCCTCATAAA
AAAAACTTCCAGGGAGCAGCTTATCATTCTTGGTTGATATACTCAACAAATGTTTTCAGATGGCATATTTTCC

TGACAAATGGAAAAAATGCCAAGGTTGTACCAAATTTTAAACCGGACAAAAATCCTGCTGAAGCTTCCAGCTAT
CGCCCAATCAGTTTGCTTTCCGTAAACTTTTTGAAAAGGTCATTTTGAACAGAATGATGTCCACATTTGAGAA
AATTCATTTTTTTCCAATGAACAGTTCGAATTTTGCCATGGACATTCATCAAATTTTACGTGTAACAAATTTG
ATTCGTTCCAACAAATCTTAAAGCTATTCTACTGGTCTTGCTCTTCTAGAAAAAGCATTCGACAGTGTTTGGC
ATGAAAAAAAAATCCAACACACATTGCTAGAATAAGTCAAAGTTAGCTATCAAATGGTACACTTTAGATTAGTT
ATCAGAACTCCAGGTCTGAAAAACTTCCTGTAAGAGCTGGTGTTCCTCAAGGCAGCATTTTGGAACCAATATT
ATACAATATTTTTACATCTGACTAACCTGAGTTATCTCATGGATGTCAAAAATCCTTGTTTGCGGATGACACA
GGCTCTCCGCCAAAGGACGAAGTCTGCGTGTCATCTGTAGTCGATTGCAAAATAGATTGGATATTTTTTCTTC
ATACTTGCAAAAAATGGGAGATTTCTCCTAATGCTTCCAAAACTCAACTAATAATATTCCCACATAAACCAAA
TGCTCTTTATTTGAAACCTTCAAGTAGACATGTTGTCGTTATGAGAGGGGTTCCAATAAATTGGTCAGATGAA
GTTAAATATCTACAGCTCATGCTAGACAATTTAACAAATTTAACTTTCAAAAATCACATTGAAGTCATTGAAG
CCAAATGTAACAAATATGTTAAACGTCTTTATCACTTTATTAATAGAAAATCGAAACTTTGTCTTAAGAACAA
GCTTTTGATATTCAAACAAATTTTCAGGCCAGCCATGTTGAATGCTGTACCAATATGGACTAGCTGTTGTAAT
ACCAGGAAGAAAGCTCTGCAGAGAATTCAAAATAAAATTTTGAAACTTATTTTAAAGCTTCCTCCCTGGTATA
GTACCAATGAGTTACATAGAATATTGTTGAAACATTGGAACAAATGTCAAATAAAATAATAATAAATTTAGGC
TAGAATCGTTACAATCTTCTATTGATACTATAAATGTTATATATTGAAGTTCAGTTAGGTTAAGTTAATTGAA
AGCGTATTCTTTCTCTTATAAGCAGGTGAAATCAACCTACCTTGTATATGGGAATTCAAAGCTTCCAAAACAA
TTGCTTGTTATAAAAATAACGGTATCAATCTTTTGTTTTGTAAGGTGCTGTTGGAGTTTTTTTTTTCAAATTT
TCTCACTTAAATTACATTTTTTTGGAATTTTATTTGTTCATTAGCATAAAGATTAGTCAGTTGCAATTTGAAA
AAAGTAGAAACAAAAAAATTGAGTGTTAGATATGCTCGCTGAAATTCTTAGTTTGGATGGCCAAAATCAACTG
TGAGAATGATATTTATCAATTCGAAGGGTATGATCAACGAGTTAGAAGTTTTTATCTGAAAAATATATCAAAT
GCGAGTGGTTTTGTGATTTTACAACTACAGTCAAACCTCCATGAGTCGATATTCCTTGACTCAAAATCGACTC
ATAGAGCCATACTGAAAACAAAATTCCATGGTTACTATGATGGTCCCTTCAAACAACTTTCCTAAGCATTTCT
GTTCCAGTACTCGATTTTTCCATGAGTCGATGGTCCTTTGAGATATCGACTTATGGATGTTTGACTGTATGAT
ATAGTGTATAAGTCCAATCATCACAAATTTTAAAGATTCTACAGTGAAAAAATCATGCCCAGATAGCTTTTTC
GGTGACCTTGAGGTATTCCATTAGGGGAAAAGTACCAATTTTTGACCCATCCATACGATTTTGGCCTACTTTG
TATGAAAATTCGAAAATTGGCACAGAATTCTTGTGGGTTGTTGGTACTTTTCCCCTACTTGTGAATAAAGTTA
CTTGTTATTGAAATTTTTACAGCTTAAAGGGACAGAATCCATCATTATTTAAGAATCTTTGAATGCAATTTTT
CTGTTCATAATTAAGAAAATTAAGAAGTGTTTAACATACCATCGAATGAATTTAAGTTTGGACAGAAAAACTT
ATTCTGAAGTTTCGATGATAACAATGATTACAATGATGGATTCTTGATCGTTAGGAAATGAGAAATAATAGAT
TACTTAAAAGAGTACTTACAGCAGTATCTGATCGACTGGCAGTTGTTGCTGGTTGGGGTACCGGGCGGTTGCT
GGTTCGGTGTGTTGCTTATGTTGTTGCCGCTGGGGCCGTTGCTGGTGACCAACATTCCGTTGCCGTTGCTGTT
GATGTTGCTGCCGTTGGTCAAATTGGTTGCCACCTGCACTAGACTGTTTCCGATGTGTCCACCACTGACGATA
CTGTTTCCGATACTGTTCCCGATGCTATTCCCAATACTATTTACCATAGTATTGCCACTCGTTTTCATCGGTT
GGGTCATTATTGTGCGCACAATACACTTGCGCTCCAAATCGGAAAAAACTTCCCCCGGGATAGGGTTTTCCCT
TCGAAAAGTTTAGTTTCTAGCTCTTCCGCTACGCGCGTCACTCGGAAAACCTTCGCACAATTCGTTGCTCAAC
ACTCTCTACACGTACAATCCCGTAGCCCAGAAGGGGGTAAAACAACACACGACAGTGTCCTAATTTTTCTCGA
TTTTATTATATCTCTTCCACTGGTTGGCTGCGTTTCGCTCTTGAGCTTACGGTTTTTTTTTTTCTTTCTCCAC
CGTCTCTTTTCGCTTCGCTTCACTCTTCTAGGTTCACTTCACTATGGAGACCAACAATCTTGGCTTACTTTGC
CGTTTCTGCTTCTTCTGCGGATGAAGACGATGATTGGTAAAGGAAGATCGCGCGCTCTGTCCGCGAAGAACAC
ACGAACGCACAGATGAAGAAGTGACACAGAAAACAAGAATTCGTTTATAACTGTTTTAGATCTGTTAGGCCTC
CACGCGAGACTGGTCTCTTTTCCTGCTTGCGCTTATCTTGTGCGGGCTTTATAGTTGGAGATTCTAACTGGGA
TGCTATATCTTGAACTGGATGAGCACTGTTTGTTGCGTTTGGATGCTTCGCACCTTAAAGACCCACTAAGCCA
ACTGTTGGCAACACTAGATTTATAAAGCCCATTTTCGTTGCAAAATACATTGTACTACGATCACACTTTATTC
TTCTCTCAAAATCCACCGTTTTTCAAACTTAAATCCAAATCTCCAACACACTCTGTTCTGCTATCTTAAAATT
TCAGCAAACTTCTGTTCTTCACATTTGTACTTTTCATTTGAGTAGGCGTTGAACCAATCCGTTGACCTGCTGA
GACAAAGAACAAGTTGCAATGTCAAGAGTTCACCTAGAATTTTTGGTTATCCGTTTGCACACTGGGAGGGTTA
AGCCCGAGAATCTACCGTACACACACGCGAGTCTTGGTTTCCGAATCCACACCGGAGCACGCGTTACATGCGC
GTGAACTCACACGGGTCTGGCGAGATAGTGACGAGTTGTGAGATGGTTACTGGGACGCCAAGAGGTCCACCAA
AGAAAGAAGGCCCGTCGAAGTCGTACTCGTGCTCGGGACTTCTATGGGAGGAGGAGAACGGTGAAAAGACTTT
CTTTCCTCGACGCGGGTACGATCTAATCTGATTGGTGATTCTGCTTTCTGGCTGCCAACAGGTCCCTCAAGAA
ACCGCGTGCCGTCGTCGTCAATCGAATCGGCCACTCACCGACCGAAAGAGTGTACCAAAACACAACAGATGAA
AATCCCACCGCGATCCAAGTTGGCGCGGGCAGTGCTCTTTTCTCCTCGCGTGCATAACTCACTCACTCGCAGT
GGAATAAGCTAGCGCGCGCATACACACACTCACTCGTTCGAACCGACCACGCTTAAGCTAGAAAGCTTAAATT

CCTCCGAGGAAAAGCTGCTACCCGAGTTGGCAATCGTGTTTTGGGTGTTGTTGTTGTTTACGCTCTTCGTGGA
GAAGTTTTTCTTTCGGCCTCCAATGCTTCGGGGTGCTGGGAACGAGCAAGCGGAACAAGAAGGAGTTCGACTT
CTACACTTTTCAGGTTAAGCCTTCGGTTCGAGTGCGGCTGAGTGCAAAGCGACTCGACTATGTCGAACGGTAG
GGCAATGGATATGTATGCTATGCATGGTGCATGGTGAGAAACTTGGGTGGCGATGACGGGTCTCGATGTACGA
TCATGGGAACAGAAGAAAGCTGCAAAGAAGGATTGCTGAAGAAAGTTTCTAGCGAATGCATTGTGGCGATGGG
TGGAATTATGTGTGGCCATAATGACCAATTCTCAATACAGCTATCATCAAACATATTTTAAATGGGAAAGTTT
GTTCTTTTCATGGTAAAGAAGGTGAGTAGTTAAATTTATGCTTCAACAGCTAATGACGTACATATTACAGTTA
TTACTGAAACCTATTTGAACCTGGATCTCAACTCATCAAAATCTCCAAACTTTGTCGTTTATTCAAATGGAGC
ATGTGGTAGAGTTGCTATCATCATGCATAGGCGTATAAATCATCAACTTTTTCGTCATCTGAAACCATTTTTT
TTTATAAACTTCGATGGGTTTCTGTTTACATGCAGCATGTTCAATGAAAACTTTAATAGCTGCCTATTTGCCT
TTTCTATGCAATGGACTGTAAGCTCATCTGCTCCAAACTGTCTTGTGGAAATTATGGTAAATTGAACACCGTG
GCTTTTACCTAGAAAAAACAAATTCTGATCAATTTTTATTACGCACGGACGATTTAAGGCATAAATCAGAGTG
TTCGGGTTGATACGGAAATCAATTGAAGTGAAAATATCAACAAAAACAAAGATTTTAGAAAACCACGTTTGAA
AATCAGAACTGACGCCACTTTTAGCTCGGGGACTTGAGGTTTTTTAGTAAGGTGATTATCGTTATGATATGAT
GTTGAATTCTTTTTGAATGGATTACAATTATTAACAAATATGTTTTCGGTTATGCAATGAAAATTTTCAGAAA
TATTTGTTTTGCACACATTTTTGCATGATGTTTATTTTACCACCAACAGCTGTCCATTTTACCCACATAGTGC
AGGTAAAATGAACATTTGTATCGTTTTTTGCTAGCGATATAAATATGTGAAAATTTAGCTATTGTAGTCTGAA
CTCGTGTCGCTGCTATAGATAACATTAGAGTGATGCAAATTTTGAAATTTTGGCTCCCCTATGCTTAAACTAC
TTATATTATGGTAAATAGCATCCTCCCAAAGTTTAAAATAAATCGAAAGAAATTTGACTGTGCACAGGTCATT
TCAAGTTTATATGGAGATTACTATGGAAAACGCCATCCTTTTGTGTTCAGTCCTCTATCTCTTCGTCATAATG
TTGTTGGAAAAGTGAACACAATCTTCTCATGTCAAATCCTTTCAGCTACAACTTTGCTGAAGACCACATTTTG
ATTGGACGTCAGGATAAATTGCTACCTAAAAATACCCCACTTAAAAATGGAGACAGAAGCATACCCATTGGGG
CTTCATTTAACTGTCTGAAAGATTCACCTTTAAGGGAAAATAGTCCTTTTCCATACAAAGTTTAGTGCATTTA
ATATATATACTTGTTTTATCCAATCTGTGCCTTAATTTTGACCAAGTAACCATTTTTCTAATAGGCTTATACT
AAAATAAAACTTCATCATCGGCTATTTTAAGATTTTCAATTACTTCTGGGAAATGTTTAAAATTTTTACTGAT
TTACTATGAAACTTTTTCGAAAAAAATTGGAATTTCTGGAGTTTATACTTTGCAATATTGTGAGTAGGTGATC
CAATAGCTCGATTAATCTCACGCATTTCATTACCAGGCCTATGAAATTTAGGAAGTCCTTTAATTTTTGGTAA
TAAAGGGTAAGATTACTTTAGTGAGTTTACATCATCACTCAAAACCGGTTTTGATTAATTGTGAACTCTATCA
ACACACCTTGCTTACTTACTTATTTGGCTTTACATCAATTATCTTGATAAAGCCTCGCCAACAATATTTCGCC
AATTCCCTCGGTTCATGGCCGCTTCTCTCCATCCTTGACTGTGACCCATGTTCTCCAGGTCCTGGTGTACCTG
GTCAATCCACCCAGCTCGCTGCGCCCCACGCCTTCTTGTTCCGGCCGGATTCGTGGCGAACACCATCTTTACA
GGGTTGTTGTCCGGCATTCTTGCAACATGCCCTGTCCAGCGTATCCTTCCAGCTTTAGCTACCTTCACGATAC
TGGGTTCGCCGTAGAGTTGAGCGAGCTCGTGGTTCATCCTTCGCCGCCACACGCCGTTCTCCTGCAAGCCGCC
GAAGATCGTCCTTAGCACTCGGCGTTCGAAAACCCCAAGAGCTTGCAAGTCCTCCTCGAGCATAGTCCATGCC
TCATGTCCATAGAGGACTACCGGTCTTATGAGCGTTTTGTACATCGTGCATTTGGTGCGGGGGTGAATCTTTC
TTGACCGCAGTTTCTTCTGGAGCCCATAGTAGGCACGACTTCCGCTGATGATGCGCCTTCGTATTTCCCGACT
AACATTGTTGTCAGCCGTCAACAAGGATCCGAGGTAGACGAACTCGTCCACCACCTCGAAGGTATCCCCGTCT
ATCGTAACACTGCTTCCTAGGCGGGCCCTGTCGCGCTCAGTTCCGCCAACCAGCATGTACTTTGTTTTCGACG
CATTCACCATTAGCCTGACTCTTGTTGCTTCGCGTTTCAGGCGGGTGAACAAATCTGCCACCGTTTCAAATTT
TCTCCCAATAATATCCATGTCGTCCGCGAAGCAAACAAATTGTCCGGATCTCGTGAAAATCGTGTTTCGACTG
TTAAGTCCGGCTCTCCGCATGACACCTTCAAGCGCAATATTGAACAACAGGCACGAAAGTCCGTCGCCCTGTC
GTAGTCCCCGCCGAGACTCGAACGAACTGGAGTGTTCGCCCGAGATCTTCACGCAGTTTTGCACACCGTCCAT
CGTTGCTCTGATCAATCTTGTGAGCTTCCCGGGAAAGCTGTTCTCGTCCATGATTTTCCATAGCTCTATGCGG
TCGATACTATCGTATGCCGCCTTGAAATCGATGAACAAATGGTGCGTAGGGACCTGGTACTCACGGCATTTCT
GGAGGATTTGCCGCACGGAAAAGATCTGGTCCGTTGTCGAGCGGCCGTCGATGAAACCTGCTTGATAACTTCC
CACGAACTCGTTTGCTATAGGTGATAGACGACGGAAGAGAATCTGGGATAGCACTTTATAGGCGGCGTTTAGG
ATGGTGATTGCACAATAATTTTCACACTCCAGTTTGTCGCCCTTTTTGTAGATAGGGCATATAACGCCTTGCT
TCCACTCCTCCGGTAGCTGTTCCGTTTCCCAGATTCTGACTATCAGCCGATGCAGACAAGCGGCCAACCTGTC
CGGGCCCATCTTGATGAGTTCGGCTCCGATACCATCCTTGCCAGCGGCTTTGTTGTTCTTGAGCTGTTGAATG
GCATCCTTAACTTCTCCCATCGTGGGAGCTGGTTGATTTCCGCTGTCCGCTGTGCTGACGTAGCCATCGCCTT
CGCTGTCCTGACCTTCTGTGCCTGTGTTCTCTGCACCATTCAGGTGTTCATCGTAGTGCTGCTTTCACCTTTC
GATCACCTCGCGTCCGTCCGTCAAGATGCTCCCATCCTTATCCCGGCACATCTCAGCTCGCGGCACGAAGCCT
TTGCGGGATGTGTTGAGCTTCTGATAGAACTTCCGCGTTTCTTGAGAACGGTACAGCAACTCCATCTCTTGGC
ATTCCACCTCTTCCAGGCGGCGCTTTTTGTCCCGGAATAGGCGGGTTTGCTGTTTCCGCTTCAGTCTGTATCG

TTCCACGTTTTGCCGCGTCCCATGCTGCAGCATTGCAGCCCGCGCTGCATTCTTCTCCTCTAAAACCTCCTGG
CACTCCTCGTCGAACCAATCGTTTCTTGAGCTCCGCTCCACATATCCGACAACGCTTTCGGCAGCGTCGTTAA
TGGCTGCTTTGACTGTCCTCCAGCAGTCCTCAAGAGGGGCCCTATCGAGCTCGCCCACATCCGGCAACGCTGC
CTCAAGATGCTGCGCGTACGCATTGGCGACATCCAGTTGTTTCAGCCGCTCGAGATTGTACCGGGGCGGGCGT
CGGTACCGTACATTGTTGATGAAGGATAGTTTTGGGCGCAGTTTCACCATCACAAGGTAGTGGTCGGAGTCAA
TGTTGGCGCCACGATAGGTTCTGACGTCGGTTATGTCGGAGAAGTGCCGTCCATCGATCAGAACGTGGTCGAT
TTGCGATTCTGTCTGCTGAGGTGATCTCCAGGTGTACCGATACGGGAGGCTGTGCTGGAAATAGGTGCTACGA
ATGGCCATGTTCTTGGAGGCGGCAAAATCTATCAGTCGTAGGCCGTTCTCGTTCGTCAGCCGCTGGGCTGGGC
GCTGAACTTTCCAATCGTCGGTCTGAACTCCTCCTCCTGGCCAACCTGAGCGTTCAAATCTCCTATGATGATC
TTGACGTCGTGGCTTGGGCAGCGGTCGTACTCGCGTTCGAGCTGCGCGTAAAATGCGTCCTTATCATCATCAG
TGCTTCCGGAGTGTGGGCTATGCACGTTGATTATGCTGAAGTTAAAGAATCGGCCTTTGATTCTTAACTTGCA
CATTCGTTCATTGATCGACCACCACCCGATCACGCGCCTTTGCATATCACCCATCACTATGAAAGCTGTTCCC
AGCTCGCGTGTGTTGCCGCAGCTCTGGTAGATGGTATGATTACCTCTAAACGTTCGCACCAATGCTCCTGTCC
AGCACACCTCCTGCAGCGCTACGATGTCGAAACCGCGGGTCTTCAGTACATCGGAGAGTATGCGAGTACTTCC
AATGAAGTTGAGAGATTTGCAGTTCCACGTACCGAGTTTCCAATCGCTAGTCCATTTTCGTCGCTGTGGTCTT
TGCCGATTGTTCCGTTCCGTATTCTCTCGTTGACGCTCCTGTGCTGATGTGTTTTTACGGTTGGCTTGCAAGG
CCTGACACCAACCCCCTAGATTTCCGGAGGACCAACACATGAGGATCTTCTCGGGTTGGAAATTTGCTCGACT
TCCCAGGGCATAGAGTATCTTCGTACCTGCCACACGATATACACATGCAAAAATGGTCATTGGCATAGTAAGC
TCTCAGTTAAAAACTGTGGAAGTGCTCATAAGAACACTAAGCTGAGAAGCAGGCTCTGTCTCAGTGGGGACGT
AACGCCAGAAAGAAGGAAGAATGTTCCGTTTTTATCAACACGATCGGCAATTTTCAGTTGACAAAAACGGAAT
AATTTTCTTAGATAAAATAGTTTACAAATTTTAAAACAAAATTGCAGTTTTGCCAGGATGATACACAATTTCA
TTATTAAAATGCACTGTATTGATATTTAGGAACTGTGATTAGCATTAAGATTTTTCGTATAATTTCATAAATA
AAGTTGATTGCAGACTCCAATCAATCAATATGATTATTTAATGCGATAAATCATCAATACAGTCGACTCTCCA
TATCTCGATGTTCTATATCTCGATATTTCTCTCTATGTCTATGATTTCTTAGGTCCCTTCATTCTGCATACAA
TTTCTCTCTCCACATCTCAATATACTCTTTATCTCGATATCCCCGTATCTCGATGTTTTCCTGTTAATTTTTC
GTTCCAATTTCCTTTCCCAGTGTTTTCTTGTGGAAGTGCAGAGGACTCCTCGGCTTCTGTAAAGCAAGTAACA
CGTAAACATTTCCCTCCCATTCCCAAATTGACCTGCATTCGGATGCAGCCGGCGCCGGTATTGTTTATTATAA
TAATGAGAGCACCAGTACTTACACATTGAGGATGCTACTGATCCCGAGTAGTGTCTGTTGGTTCCCTGTGTAA
GTACAGCTGTTCTTGCAATAACGGAGTAGCAACTGCGGGCGGTCCATCATGCTCATGCTCATGCTCATCCATG
CTCATCCTTCCGCTTCTTTTTGTCTATGGCATTGCATCACAACTGTGGAAAAGCTCTCAGCTTACTGTCCTTA
TAAACACTTTCGCAATTATCAACTGAGGGTTTTTTTATTTTGCTAAGATACATATTTTGCATTTTTGGCACCC
GATTGGGGGTTAAGGTGAAACAGTTTGGAATCATTTTCTAAGATTGCACTAAAACTTCAAAGGCACAAATCTC
GCGAAGAAAGTATCCCACGACATTGCACTTTTTATTTTGGCTTTGTGCACTAGTAGAAAGCTTAAAATAAGAA
AATCAGGAACGTTTGCCAACTATTTCTCCAGTTTTGTGCTTTTAAAAACGTGAGTAGGGGGAGAAGTCGGCCA
TTGTGCCGGCCATCTTTGGATTCCGAGATGTTTCACCTTAAATGTGAGGTTATGTTGCGAAAGTTTTGCAAGC
TTAAATTAATATTTACGGTTTGCTGTGCTTTTAATTGTCAAAATCTATTGTGTAGAATGATTGTGGTGATTTT
TCTTCACTACCACTATCATCTTTGACTGGGATGCAATAATCGCCATTCTAGAGCAGCGCCACCTTCACCGTTT
AGCTCTACGTTGGATCACGAATCGACTAGCACTAAACTAAACTAAAATTTTACTATTTTGTTTGGTAAAACAA
TGTTAAATAGGTTTATCAAAAACACAAAAGTGCACAAACTCCAGAACAAGCACAACCAAATAAGATTTGTTTT
GTTATAATCTCTCTTGTTAAGTTTTAGGAAAAAGCGTAAGGCAGTTCATATAAGCTCTTTATCGAACTTTTGG
TATCCACGTGAAGAAGAGGTGCGTGATTGCTCAAAAATCAATATCCCAAAAAATTGGGATGAGAACATGCGTG
ACGTGAGAGCATCAAGTATCAACAGTTTCTCAAATTATCAGCCATTTCAGTCCTTTTCCATTTTCTTTGATGC
AAACTTATTGTATGAAGATTCCAAATATTTTGAAAGATTGTTAAGTTTTGCTGGACGTCTTTCCCTACCCTTG
CAACAGGACCTAATCAATGGCTGTTGCTATAATCTGTGCAAGCTTAAGAGATGAAGGATAATGTACAAAATAC
TACGGGATCGACAGTCTTATCAAAAATAAAATAAACTCGATCATGTCTCTTGCCATTAGGACCCATTAACAAA
TGTCATAACGCTAGAGCAGCGTGCTACCTTTGCCTCTTTGCGTGAGACTGAAAAAAAAATACTAATCAGAGAG
GCATCAAAAAATCCGCGAGGAAGATGCAGAACAGAGCATAATTCAATAACAAAAGTTCCATAACAACTCCGCG
AGGGCTGTTTTGTTCGGGCGGGACACTCAAGAGTTTTTTTTAAAGTGTGAGTAAATGTGAGGATAGCAACAAG
AGAGAAGAGTACCAGAAAAAAAATCTTCGTATTTTTTTGCACTCTCACTCGAATTGTTTGAAGATCTGTGTTGA
AAATGTTGAGTGCAATTTTTTACTCCTCAATAAAACATCAAAATCGCCTCAATTTTGCGTTGCATAGGAGTTTT
GTCAAGCCTGGTTGCTAGGACGTGGTCAGCGCAATACTCGACTGTTGAAAGATGCGTCAATCTTGACCTTCAG
CCCATGCTATTTTTGAAAACTCTAATTGATTGGCGGATAGGAAGAAATCAAACCTTATTGAATCGTATATGAC
TTGACACCTACCATGTATCGTAGATGATCAACTCCAACATAATGTCGAAAATTCAACCGTGAAGTTATTGAAA
AACATTTAAATAGCTTCAAATAAGTTTTTGGTTTATATTTCAAGCATTTCTAAAAATAAAAATAAATACTTTT

TTTGTGTTGATAAAAACGGAATAATTTGTTGACGAAATCGGAACGTGATAAAAACGGAACATACCTGTATTCC
ATCGGTGAAATTTTGATTTTTTTTTCACTTTTTAGCTATTTTTCAGACTGACCTCCATGAAAATCCCGTTCAT
TGATGCATTTCAGCCCATACAAAATGTATGGAAAAAAATCACCAATAGAATATTTTAAAACCTTCCGATTATG
AAAATATTGATGAAATATTGATCTCTAGCGAGAAAGAAACAGATGTACATTCGATGTGTTCGTCTGATTCAGA
CATAGCAAGCGTCGCGACGCGTCGTCTCCGGATTCCGGAGCAGACCGCTCACCAGACTTTTTTTTGCAGTTGA
GGAGATTTTAACGTCAGAGGAAAATAACCATCATCGTTTTACAAATTTTCTAAGCAAGTTTTTTTGCTCAAAA
TTCATGCAATGTTCAAGAAAATCTATCATTGCACTACACTTGCTATCTGGATTTTTATGAGGGTTTCTTTAAA
TTTGATAAAAAAAAATGTTTTTTTCTCTTTCGATGATTGCTGATGATTGAATGATGGATTCTTGATCCTTAAT
GTACAGTACGACAAGAAGCGATTGAACCAGGGCCAAGTCCAATAGAGAAGGCTGCTTCATTCGGCGTAGATTC
AAAAAAAATGTTGGACAGTCAAGTATCAATTATTGTAATTGAAATATGGCATAGTCTAAGTTAATCAAAGAAA
ATCCAAGAAATTTAAGAACATCACGTTTTGGTCTCCAATATGCCCACTGTGCAATGTGTGAAGCATAGAATCC
GCCTTTCTCTCTTAGCCATCATCAACGTGAACATCCAAGATCTATCTTGCGTGTACAAGCCACTCAGTTTTAC
CAAAACGAAGCACTCTCTTCTCACTCAGTGCCGATGCAGCCGTAACCCACCCAAAGACTAGACCCGCGAGAAG
AAACCTGACCACAAAGAACTCACACACGTGGGTTCGTTGTTCCAAAGACGTCACAGACCTCAGCTGCTGGTAG
AGCTTTCCATCTCCCCTTTCACGCGCGAACGTTCTCTCGCTTGATCTAGCAAGCAACAAGCAACCACGCGCGC
ACACTGACGATCGTACGAAGAAAGCAGGTCAGCCGAAGATGCGCGAAGATCACGTCCATGGTTTGGAACTTGG
AAAGGTGGTGGTGGCTTTGATTCGTTTTAGCTCGATCAAGCGATCAGCAGCAGCAGCACGCTCGGCAGAGAAC
GAAGATTCTTCGGCGGACGGTTCGGCGAAGAAAGTTCCAAAAAGAAAGATCGAAGAACGATCGCAGCAGCGAT
CATCGGCGCGGAAATACTAACAGCTTCGGGCTTGCTGACGATTGTGCGGAAGCGGGGTGAGGACTCACAGCAC
CGCGCGGCAGCTTTTGTAGATCTGGCCTTACCTTGCGTACCGTTGGTTGGTTGGTTGCCGAGGATTGCTGCTG
TAGTGGTGGAGAGAACAAGCGGGGTCCATGATGGATGGGGACGGAGGAATGAAAAAGAAATTTCTTGGCTTGT
TCGCCCGAGGAGTACGAAGAAAGTGCACAAAGCAGACTCGAAGAAAAGAAAAGAAATGATTTAAATATTTATT
CCGTTGGTTGGTTAAGACCTTTTTTCTTTCCTCTTGTATGTTATATTTTCTTTTTGCACTTCAGCGGGCTGCG
GTGGGAAGGAGGATACCGCGCTTGACTACATTTATGCGAGAGTTAACGAAGGTAAGCTGAGAAGTAGCAGAAC
ATAGTCTCGGCGAAGTGGGACAGGGGAGGGGAGGGGAGTAAGAGAAAAGGTGGACGGAGGGCACAAGGGAAGT
AAATCAGAAAAGACCTAACTGTTCGTGTACACGGCTGGACGATCGGATGGATCTTGTACTGGTCAGAGAACCA
AGCCACGGAACGGGTTACGGGAAATGGACTTGTTTTAATACAACAAGCACCAACTCCGAAGAACTTTACCAAA
GCGGGTATAAACTTCACCAGGCGCGAGACCGGAATGAGGACACCGCTTGTTTATTCAGATCTCACTTAACGAA
AAATTATGAAAGTTGTTTTCGGACGTGGGGAACATTTTTGAATACTTTTCCAGCTCTACGGGTTGAGTTTCTG
GGAAGAAAGCTGACTATTTTCGAATTGTGGACAATTGCTGCTGCTATCGAGAACATGTGTTTTTGTGTTGAAT
TAGGGTAAGTTGACAATTATGGTACAGGCTAAAGTCTGTCCCAATGATAAATTTAATTGTACATTCATTATGG
GTTTCGCAAAAAATTGGGTTGATTTGTAGCCTGTGCAATAATCGGCAATTTACCCTACATAGTAAATAAAATTC
TTATTTCATATGTAAAGTAAGATGAGAGCAACAATTTGAAGTTAGTTAAACAATGTATACTCCTAAAAACTAT
TACAACAATTTAACAAGTTTTCGTAGACCAACTATTAGAAAATTTAATAAAACTAGCAAAAAAATCTATTTCC
TTTGTTATTGATGCAAGCGGACAACGATTCAGTAGATTTCCTAAAGTAAATATCTCAAGAGCATACATGACAG
CAAGTTGGCCTACAAGGTTCAATCAGCATTAAATATCTTTTTTTCAAATATTATTTTTTCTCATAATCAATCT
AGCTTATAGGTATATAGCCTTATTTTTTAGTTTTCATTATTTAAAAAAGTTTCTTAAATATGCTGGCATATAT
TTGAATCCTTTAAAACAATGGCGCACGTTGTGCTGAAACACACATGCTATCGAACTTGTATGAATCTCTATTT
TTTTTTATAATTAAAAGTTAGCCTTGGTAGTCACAGTTTGCGGTACATTATAATAACCTTACATCGACGAACA
TTTGAATAATGTTGATTTTTGATTTTTTTTTTTACCTATGCAAATCTATGGGTTCATTAGTAAAGTGTCCATT
CCGACAAAAAATCCCGGAATTTGAGAAATTTCTGGATTTCCCGTTTCCCTTGATACAAGATCTAACGTCCCGG
GTTACCGGGATTCCCAAAATGAACGTAAATTTGGATAAAAATCACTAAATTCTTGTAAAACAATGATATTTAT
CCTCAATCTCAAGCTCTAGGAAAAGCATCATACAAGTAAGTAAATAAATTGAATATTAATATCACAAAGAGAC
TAAATTACCAAATAACGTTTGCTTCATTTAGAATTGGAAAAAAACTTTTGCTCAGTAGGACGCCAGTTTTCAA
AAATCGAACTTTATCAAAGCTTTTGGCCGACTTTAAGTACAAGGACAAAATTTAAATAAAAACTAAGCGATGC
TACGTTTAGACTGCAGCTGATATTTAGCTGTTACTTCGATTCTCATTGGTTTAAAACAGTGTAAGAACATTAC
ATAACGTTTTCTTATCTATTATTCTTCTGGTAAAGTAATGGTATTCAATCTACACGCTCAAAAAAGAATTCTG
GAGAACGTGACACATACACATGATCTAGTTCACGGTGTATATTTTTGCTTGTTGAAGAGTCCACGACTGCTGT
TCACGGATACGAGAACGGATTTTTTTTCTGTGTAGTATTTACTGTACTATGGTGTCCAATTTTTATATAAAAC
AAACATAAAACAAATGGTAAAAGTTGTATGTAACACATGAGCTTAGCAATTGTTGGCCACGTCGTGTGCTCAG
TAATATTTAAAACCGAATCCCAACAACAAATATTTTGTTGATTTTCGCCAAAATATTTCCAAGAATCGTAATC
TCACTCAAAGCAAAAATGCAAAAGGAGCGTTGTTCTAGAATGGTGATTAGTTCATCCTAGTCACAATTACAAG
TTATTAATATATAATTCAAAGAACAGGGAATCGTACGTAAATATCCATTTGATTTCGAAAACTTTCGGTTCAT
AACCTCACATATAATCGCTAATACTTGCTGAGATCTTACCGAACGCGAGTACGACAGCTGCCCAAGCCACGAC

GTCAAAACCATCATAGGAGATCTAAACGCTCAGGTTGACCAGGAGGAGGAATTCAGCCCGACTATTGGAAAGT
TCAGCACCCACCGGCTGACGAACGAAAACGGCCTACGACAAATTGATTTCGCCGTCTCCAAGAATATGGCCAT
TCGTAGCACCTACTCCCAGCATAGCCTTCCATACCGATACACCTGGATATCACCACAGCAGACAGAATCACAA
GTCGACCACGTTCTGATTGATGGACTGCACTTCTCCGACATTATCGTCGTCGGGACCTATCGTGGCGCTAACA
TCGACTCTGGCCACTATCTGGTGATGGTCAAACTGCGCTCAAAACTCTCCGTCGTCAACAACGTACGGTACCG
ACGGCCGCACCTGTATGACCTAGAGCGGCTTAAGCAACCGGATGTCGCAGCTGTATACGCGCAACACCTCGAG
GCTGCATTACCGGAAGAGGGTGAGCTGGATGAAGCCCCTCTTGAGGACTGCTCCAGAGCAGTAAAAGCAGCCA
TCAACGAAGCAGCTGAGAGCAACGTTGGGTACGTGGGACGGAGTCGACTGAACGATTGGTTTGACGAGGAGTG
CCAGGAGGTTTTGGAGGAGAAGAATGCAGCGCGGGCGGTCATGCTGCAGCAAGGGACCAAGCAGAACGCAGAA
CGTTGTAGACGGAAACGGCAACAGCAGACCCGCCTCTTTCGGGAGAAAAAACGCCGCCTGGAAAAGACGGAGT
GCGAGGAGATGGAACAGCTGTGCCGGTCTCAAGAAACGCGTAAGTTCTATCAGAAGCTTCATGCCGAGGTTTG
CAGGGATAAGGATGGGAGCATTTTGACGAACGAGCGTGAGGTGATCGAAAGGTGGAAGCAGCACTTCGACGAA
CACCTGAATGGCGTTGAGAGCACAGGCAATGAAGGTCAGGACAATGGAGGAAATGCCTTCGTCGGTACTGCGG
AGGATGGAAACCAGCCAGCCCCCACATTGAGGGAGGTTAAGGATGCCATTCACCAGCTCAAGGACAATAAAGC
TGCTGGTAAGGATGGTATCGGAGCAGAACTCATAACTCAAAGGCTGGCCATTTGTTTGCACCGGCTGATAGGC
ACAATCTGAAACATAAAACAGCTACCGCAGGAGTGGAAGGATGGGGTGATATGCCCCATCTACAAGAAAGGTG
ACAAGTTAGATTTGAGAACTTTTGTGCGATCACCATTCTAAATGCGGCCTACAAAGTATTATCCCAGATCATC
TTCCATCGTCTGTAACCCATAGTAAGCGAGTTCGTGGGAAGTTATCAAGCCGGCTTCGTTGACGGCCGATCGA
CAACGGACCAGATCTTTACTGTACGGCAAATCCTCCAGAAATACCAGGTCCCAACGCATCACCTTTTCATCGA
TTTCAAGGCGGCATACGATAGTATCAACCGCGTAGAGCTATGGAAAATCATGGGCGAGAACAGCTTTCCCGGG
AAGCTCACGAGACTGATAAGAGCGACGATGGAAGGTGTGCAAAATTGTGTGAAGGTTTCAGGCGAACATTCCA
GTCCGTTTGGATCCCGCCGGAGACTACGACAAGGTGATGGACTTTCGTGCCTGTTGTTCAATATTGCGCTAGA
AGGTGTTATGCGGAGAGCCGGGCTCAACAGCCAGGGTACGATTTTAACGAGATCCAGTCAATTGGTTTGCTTT
GCGGATGATATGGACATTGTCGGCCGAACATTTGAAAAGGTGGCAGACCTGTACACCCGCCTGAAACGCGAGA
CAGCCAAGGTTGGACTGGTGGTGAATGCGGCCAAGCCAAAGTACATGTTAGCTGGTGGGGCCGAGCGCGGCAG
GGCTCGCCTAGGTAGCAGTGTTACGATAGACGGGGATACGGTCGAGATGGTCGACGAGTTTGTTTACCTTCGA
TCCTTGCTGACGGCTGAAAATAACGTTAGTCGTGAAATACGGAGGCGCATCATCAGTGGAAGTCGGGCCTACT
ATGGCCTCCAGAAGAAGCTGCGGTCAAAAAAGATTCACACCCACACCAAATGTACCAAGGACAAAACGCTCAT
TGGGCCGGTAGTCCTCTACGGGCATGAAACGTGGACGACGCTCTAAGAGGACTTGCAAGCACTTGGAGTCTTC
GAACGTCGGGTGCTTAGGACGATCTTCGGCGGTGTGCAGGAAAACGGTGTGTGGCGGCGAAGGATGAACCACG
AGCTCGCCCAACTCTACAGCGAACCCAATATCCAGAAGGTGACCAAAGCTGGAAGGATACGATGGGCAGGGCA
TGCTGCAAGAATGTCGGACAGCAACCCTGCAAAGATGGTGTTCGCGTCGGATCCGGATGGTACAAGAAAGCGT
GGAGCGCAGCGAGCTAGATGGGCGGATCAAGTGCGTATCGATTTGGCGAGCGTGGGCAGAACCGAGGATGAAG
AGATGCGGCCACGAACCGAGTATTGTGCCGGGAAATTGTTGATTCATTGTTATCTGTTTAGATGTTAACTAAA
TAAATGAAATGAATCTTACCGGAAAAGAAGTTTGTTGTTCGCTCGGCGTAGTCAATCTCAGTAAAAAGTATTG
AGGAATACTGAAGTCCGACGAAAAAAATAATTTTGTGGTGTAGTGGTTGGCACACACGCCTTTCACGTCAATG
ACTTCGGATTGATTCCCATCCCCAAGATAATGATGATGGTTTACACTTAAAAAAATAGGATTACATTGAAATT
TCACTATATTCTCAACGGTGAAAAATCATAAATAAAAACTCGAAAATGGCCAACCAGGAATTGAACTCCCTAC
TTATCGGAAAGAAAAGCTTGCTGCCAATAAGCCAGCTTTCACTCTTTGTTAGTGGTGTTCAAAAACTGAAACA
AAAGTAGCTCATGGCTGCCAGTGCCGTTGTCAAATGATTTCACAGAAGTTTGGCGAAAATAATGATGTTACCG
AACTGTGAGTATTTCACATGGTCACAGTAAATTTGTTTGTTTTACGGAAACATTCCCGTTAAAAAAAAGTAGA
AATCACGGATTTTGTCGGAAAACTTTACAGGAAAATCTGTATTTTTGTAGTTTACAGTTAAAATTCGGTGATT
CATTTCACAGGCTACTGCGATTTGTTCTAAGTGTGTTCTATAGTATATGAAATCTGTGATGAAATCCTTGGAG
TAATTTGGTGGAAAATCATTTCGCTGAAGGGGGTGGGTTGGTGTCCTCGTAATGTTACGGATCATACAAAATT
AGTAAAATATTCATACAAAAATCGTTACGAGGGGGTGGGTAGGTGTCAAATATTGCCGTTTTGGGCGTTATGA
AATTTGCGAATAAACCCTGAAAGGCCTTGACATATTCCTGTGTTTCGAAGGAAACACTAGAAGTAAGGAAATT
TTTGTAAAGTTTCTTAACGATCGCCTCTAGAGTTGCTTAATTTTAATCATATCAGCTGATGGCTGATGCTCTA
AAACTATCAATATCACTTGCGAGCTATCAATATCACTTTGATTTGACAATCAACTGCAGTGATGCGACATCGC
ACTCTAGATTATAATCACTGCAGAATGAGTGATCACGTGGTAATTATCCAAGCTATTAATGTTTTTCAGTAAC
CAACATTTATTTGCTTAATTAAAGGCTAAACTTAACCCATCAGTGATATCCATAGTCTATCGCCATCAATGCA
CTGGCGATGGAGTAGGCAGCATGATGTTGATGTGATATAGTTGAATCCGAGTCGTATCGCAGTCGACCTGTAC
AAATCAGCAAATTTTAAGCGTTGATAGTGACTCTGTTTTAAATTCACTGAAGAAAATCGTCTAAGAATGCTTT
AGGATTTTCTTGAGAAATGTTTGTGTAAACTCTCGGTAAATTTCGAGAAGAAAATATTGTTTCAGGAATCGAC
ACAATACCCTTCTTCTTGTACTCCTTATTCTAAGCATGACATCCTCACCACGGGAGACAGTGCTTCCTCACTC

CACAAATGGTGATCAGGGAGGTCAGAACAATGCCTAGCCCGGAGAGATCTAGAATTGAGCCTTAACACCTTAA
AAAAGGGTGTACGAATATGTTTTCAAACTGCTGCTCGTTGTAGTCATTTGCACTGGATTACCATCACCGTTTT
TTTTTAATTTTTTAATAAAAAAACGAACAGGATAAGTAGAAACATTTATGTTGCAATAATCCCAGCTGAACAA
CTGACGAAGTTTTAACTTTTGGTGTTGTTTTTGTGATGTTTGAGGAAAGATTTTTCTATGTTTAAAGCTTCGG
CGAAAAAGAATCAAGTTTTTTTTTCAAAAAATGTTTTCTTAAAATACATATGGTGAAGTGCTTAATAAATGGT
TACCAATACAGTACACCTACCAATCATATTTTTGGTATCAAATACTTCGTTTAACTTACTTTCCAACATCAGA
ATCAATTGATCGTTACTTGTTGGATTCCATATGATGTTCATATAATCTAGAAGCTGATAAAACTTTATTTTCA
TAAATTAGCTAAATAGTATAAGCTAGATGGGCATAAAGAGTAGAATGCTACATTATTTCCCTTCGATTCCGAA
GCACAAAAATCCAACGATCATAGCGGAAATCAGCCAAGAACCGGAGGAATAACTTTCAGTCACCAGCATCAGC
TTAGTGCTGGGCGCGATCCAAGTAGGCAAGCATTTTTTTCCACCAGAAATAAAAGCCACATAAAGAAGTAGGC
CACGGCGCGGGAGCCTCCGCACATTTCGCCGACCTAGAAGAAGAATACCCATCCATTCCATTCATTCCCGTTC
CTGTTGGCCCGTGCAGTCAGCGCGAGATGATCACATCGGGGAAACCTAAAAGGTGTTTGTCTGCGGTAGATTC
CACCGGTGCACAGTGGTTCTATTTGTTCTGCATAGCGGTCATAAAACAGTTTGTACAATATCTGATTGAAAAT
ACTACCAGAAATTGCCAAAATCAAATTAATAGATAGTACAGTCGACTCTCCACATCTCGATGTTCTACATCTC
GATATCTCTCCTTATGACGATGATTGCTTCGGTCCCTTCATTCTGCATACGATTTCTCTTTCCATATCTCGAC
ATCCTCCGTATCTCGATATCTCCATATCTCGATGTGTTCCTGTTGAATTTTTGTTCCCAATTTACTCTCTATA
TGTCGATATGAACATTATCAAAGGAGGGAAGGGGGAGGAACTGATGATGTATTTAAAAATGGTTCACGGTAGA
CCGGATATACCCCTGCATCTACGCCAGTTTATTTGGGGAGGTGTATTCTTCTATTTATGACCTATGGGTGGTA
CCACTGTGCGGTGTGCTGCTGGCGTTGTTGGGTTGTGTGTTCTCATGCAGGTCGGTCGGGCCTCGGCGCAAGC
ACCAGCCAGCAGACACATCATAGTGGTACTTACTTACTTGCAACTCTTTTAGCTTTTGTTGCTTTTGGCTTAA
AAGAGAGATGGTCTCTGAAGAAGTAAATGCCTCCTAGTAGCAATTGAAGCCGAAACCAGGTGAAATGGGTTGA
TTCGTTGATTGTTTTAAAAGCGGTTTGGAAGAGTGCCGTTCGGTGGATGCGCTCGGTTTGGGAAGGTTATTCG
CTAAATATCTACGTGCCTAGTGAGATTTCATTCGTTCATGACTTTTTTCGCGTTAGAATTAACGCTGATGAAC
ATCAATAGGTGGTCTGATGAAATTTATAGACACATGTGGTGCTGATGCCCCAATTGATGGATCAAATGATCGA
AAAGAAACTATGATGATGATTTCCGAAACGGAAACTTCGGAACACGACGTACATTTTATTACTTTTGGGAATC
ATTTGAAGAGGATTTCAGAATGTTAAATATGTGCTTTTCTAATTTTGTCCAAAAGCTTGCTACAAGATCTGCA
AAAGCAGGCTCTAATAGGATAAGGATTTACGTCAAGGTATTAGCTAGATTACAGCTCTGCTTTGTTTTTGAAG
ATTATTGTAATGAAGTACACATAGATGACTGGGTTAATGTACTACCCATTCATACCAAATAATTTGATGTTAC
CAATCTAATGATTGATGCAATGTTCAAAACCACCACTATGACTGAAGCAGTCCAAGTTGGTGTAAAATTTATC
AATAAAATTGTTATTGAATTACGTTTCTCTAATGGATCCAAGAATTTGTTTTCATATTTGAATTGAAATATTC
GTTCTTTTAATGTTAAAGAGCACGGCGTAAACAAGACAAGGCAGTCACTTTACTTGTGTAAACATCAAATGGG
GCTCGGCACTGTTTTGATTTGTATGACTTTTTGACGTTTGCTGGCCTTGTTGTTTACTAATTTTATGAAGGAG
TGAAAGAGTGAGACTGATTTCTGTGCAGAGCTCCATATGGTTGTAAACGTGTCACGTCATTTCCAGGGTTGGA
AAAAATCTTACAGCCAACTGTACTCACTAAGCCTTAACGGACATGCCCACAAATTTTGACAAGAAATTGGATG
TTGGAAGTCATCCAAATCATTCTGAAATTCAGGTCCCTATAATCTACCAAATCTACTACAGTCTACACTCACC
CAATTCTCTTGAAACCTTATCGTTCCTATCCATAAAGCTTCATATGGTTCGAAGAGTGTACTTACAGCTATGA
CTATTGTTAAGCTTAAGGTCTTCATCACATACTGAAGCTATGGGCATTAATTTCTATATGCAGCTTGACTTAT
CATTCAAACAAAATGTTTTCAATACATCCAAACGTGATCACAAATCTCCTTTATAGTTATAAAAAATGTTCCA
AATGCTTCCAAGCTTATTGATTGCAATATGAATTTTAAATTACATTGATGGTTTACAATATCTACTGTTACTG
TTTCTCACTTATTTCCAACGTTTAATATCACAGAATCTCTGCAAAGCTTTGGAAATGTGATTCGAATGTTTCT
TCTTCTTTCTGCACTTACGTCCCAATTGAGACAGAAGCTGCTTCTCAGCTTAGACAGAACTTTCACAGTTATT
TTATGGGATCTTTTCTTTCACAGATATTTACTGGGAACCTTCGATTTATCTATGATTTGGAAATCAAGTAAAT
TTTCATAATAAAGAGATCCTCAACCGGTGGGAATCGATCTTACAAGCTTCAGTTTAGTATTGCTTAATGGATG
CGCGTTAGGCACTATTTGGGCCTTTATTCGGATGTTCAATCTTATAACACAGACAAACAGATACAACTTTATT
GTGCTTTAACAGCCTCTCATAATAAACGTTGCCTCCACAGAATTAGCTTTTGTTTATAATATAGTTCATAGTT
TCCCAAACTGTGAGTCGTGACCCACAGGGGGTTCACTTGAAATGACACTTGATGTGACATTTGAAGCTTTCTT
AGAAAGATGTTAGAAAAATGAAACTGTCAACGACAGCTGACGATTGCTTTTTTTTTAACAATGATCAATTTTG
CGCAAATATGTACAAGGAATTTTGAAAGATGTCGAGACTTGCATACTAAATTTGTATGTCAAACTTCTGAAAC
GTAATTTAATTTAGCCATGGAAGAAATTTGAAATATTTCCAAGAATGTGATTACAAATACAGTAATTTTGATG
ATTTGTCTTTTGAATGCCGTCAAAAGATATTTTTTATGTTTGCCCCAATCGGAGATATTCACAATCGAAGTAG
GCATATTTTTGTGAGAAAAACGACAATGGATCAATGCATGAGTTCACTCTTTGACGTTTGAGCGGTGCCGTGT
TATGTATGTGACCATGGCAACGAGTGAATTCGGCACCGCTCAAAAGTCAAATTAGTGAACTCGTGCATTGGTC
CATAACTCCCAAACGGAAGGAGATAGCAAGTTTTTTTAGTCATCAAATTACGCATATTTCAAAGCTCTAAAAG
TGTTTCATAGACAACTTTGATGAGATATTTGAATTGAAAACGCTACAGCCAGAAAACCAGTTTTGTTCGGACC

ACCCTATGTCACCGTAGGAAAAAAAGCTCTAAATCGGTCAATTTAAGAGATACAAAAAAAAAGTTTTTTTGG
CAAAGTAGCTTGAAATTGAATGGTCTACAACTGTGCTCAAGCATGTACCATTTCTATAAAAACGTGTTCCACC
CTAATTTTCAATAACACCAAACAAAGGGCCTTACTAATACAAACAACTTTGTAGAAGACCGTTTTCGTCTAAT
GTTTCATTCTAAAGCTCAAAATGCATCTCTCTGATTCCAGGACCACTGTGCACTGGCCCAGAATCAGAATTTA
GCCAGACAAAACCTATAGCGCTTTAGAAGTGCATTTCTCCGAGTTGGTGTCAAAAGAGACTTATCTTGAATTT
AGTTGATCTTTAATTTGATGATAAAAGTTAGATGGAAATCTCGCCGCGTCTTCGGCAAAGTTTTATAAGTTTT
TAATTTATTTGTCAAAATACGACAAGGGTCTTGGACAAACACCAAAGTTCTAGGATTTCAAACATAAAGTTAT
GTTAAAAAATGTGAAAATCACTTGAATTTTACGTTTTTAACACTTTTTACGTCAAATTTTTCATTTTTAACAC
CATGCATCACGGAAATTCAAAAACATCCATGTAATGAATAAAAAAAGTCTTGTTTTTGTTAAAATACTTTGAT
TTTTGAATAAATAATTGTTAGATTATCATTATTTTTAACATAACTTTACTCAAAAAGTTGCAAGTTTTTGCAA
AAACTTAACAATGTTTCAAAATACGCTATATTTCATCTACTAAATGTCAAAGTTTGCCGGATGTGTATTTTGA
TATAGTTGAGATATCTGAATTCAAAACAACTAACTTTGTCATATAAATAGAATAGGTTTATTATTAATTTGAT
CATTATATTACAATATTTTCGAGATTTGTTAATTAAATACTTTTTGTTAGTCATGTGTTAAAATATATAGAAA
TTTGCATTTTGTCTTCTTCAGCCGTGTCAAATTCAAACGGTTGTTTTTATAAATGGTTTAATCACCGCTATAA
TTAACATGAACTTAATTAGCCATCTTAAAAAGTCCCACAGAAACATTAAGATTGAACAGATTGGTGACTGACA
TGGATCCATTTTATTGTATATGGATTTCCGTGTCGCCAAAATCGAACAGTTTTGTTGCCAAAACCGATCCGTG
CCAAAATCGAACGGGCACAATTCATCGTGTCCGGTTTGTAGTGATAATCACACTATTGGTGTAGCTCATCTGT
GTATATGTCATGAATATATATGTATATATTCGTTTCAAATTATATCGTTATACAAAATGAATCCTTTCCCATA
TCAAAACTCCCAGTGTGTTATTGTGGAAGTGCAGAGGACTCCTTGGCTTACATTAAGCAAGTAATATGTCAAC
ATTTCCCATCCCCAAATTGACCTACATTCTGGAGCAACTGGCGCTGTTATTGTTCGTTCTAATAATGAGAGCA
TCAGTTTGTTGGTTCCTTATGTAAATAGAATTGTTCTTGCAATAACCAAATAGCAACTATGGGCGGCCAATCA
TGCTCATAATGAATTTTGTAAGAAAACAACAATAAATATATTCTATTTATGCATGTAAAAAATGGTTAAATTG
AATTCAGATATCTCAAATATATCAAAATATCCGGCAAACTTTGACATTTAGTAGATGAAATATAGCGTATTTC
AGATCAGAACATTTTAAAATTTTTGCAAAAACTTGCAACTTTTTGAGTAAAGTTATGTTAAAAATTATCGTAA
TTTAACAATAATTTGTTTAAAAATCTAAATATTTAAACAAAAACAAGATTATTGTTTCATTCATTACATGGAT
GTTATCGAATTTCCGTTATGGATGGTGTTAATAATTAAAAAAAACGTAAAAAGTGTCAAAAACGTTAAATTTT
TCACATGATTTTACATAGGGGAAGTGGTGGTAAAATGAACAGGGGTGGTAAAATGAACACCGTGCCTATAACC
GAGAAAAATCAAATTTCAATGAATTTCTATCATGCACGTAAGATTAAGAGCATAAATCGGTGTGTTACGGAGG
TCAATTGCAGTGAAAATATCAACGAAAAACAAGAATTTAGAAAATCACGTTTGAAAATTAGAACTGACGTAAC
TTTTCGCTCGAAGATCTTGAGGTTTTTCATTGAGGTGAGTATCATTATGATATGATGTCAATTCTAATATTTT
TTTATTTCTGTTTGAATGGGTTACAATCTTTAATTGATATATTTTCGGACATGCAATTCAAATATTCAAAAAT
ATTTGTTTTGCTCACGTTTTTTGCAGGATTGTAACTTAACACTAGACTAAGGACAAGCATGCTCCAATAGCAC
AGCCGAGAAACATTCATGACGCAAAGTTTCAATGGCTGAAGCGGGAATCGAACCAAAAACCCACGCCATGATG
CGCTTTTCTGCCTAACGACGCTAATCGTACAGTCATGAAGCCCACATCATTCCTGGAATGACCCTTGAACGAA
TATCTGGTTTAATTCCAAAAAGTGACATAGACAGGATTTGAACCCGTATAGGCCTGAGTGAAAGCGAAAATTC
TAAAACCTTCACCGCTCAACGAATTCTTAATGGATTCAAATGATGTTTTGTCTGTATACTGGCCAGTGCTGTA
AACATTCGACACTTTGCGCGACGTTCGTTTCGTTGCCATGGTCTGCAGTCACAGCACGAGCATTAGAATGAAC
GTCGACAAACACAAAAAAGTGTCAATTGAATGTCGTCTGACACGATGGCATTTGCATAAATCATAAGTTTTGC
ATATAATTCAGACATCGGATCATAAACAACATGAATAGTTTGATCTTGACTGCTATGTCGAATGTGACACACA
TACAGTGAAAGCAGAACAGAAATCAACAAAACCGCAAAGTTTCCTAGCGAAGCTATCGTGGTCAGTGGCATTC
AATTGACATTTTTCTTTTCGACATTCGTTTGATCGCACGTGTTGTGCATGTCTGTGGTAAGCGCTGCAGAATA
TCAGCGAAAATGTGTCGACTACCGTGATTGCTTACAACACTGATACGACTTGACCCAGTGACCCATCGGAATA
ACTCCGGCCACAGGAATATTTCCGAGTTCTTTTGGCCACTTCTAGAAACTAGATATGGGAGCCAGTAAACTGA
CAAAAAATCATTTAAATGCGGTAAGAATTCTGTGAGTGGTGAGGGTTTCAGTATTTTTGCTTTCACTCAGGCC
TATACGAGATAAAAACAATTCGTGTTGAAAATGTTTTGAAGTATATTCACTTAGTGAGGGTTTCAGTATTTTT
GCTTTCACTCAGGCCTATACGAGATAAAAACAATTCGTGTTGAAAATGTTTTGAAGTATATTCACTTGGAGAA
ACTATTGGAAAATAATAACGCACCATAAAAATGATTCTGATTTATTAAACATTTAACTATAAATCAATAAAGT
TTTCAATTTACTCTACAATTACCGTAAAATCGGGTGTAATTGATCAGAAGGGTGAAGAAATTGATCATCGTTT
CACACGATTTTATTTATTCGTAATGGAGCACAAATATCAATGTAAGCTGCAGTAAATGAACGTTGCTTGTCGT
AACTATTGTCGAATTGCGTGTTGTGAAGTTTTTTGCGTCAAAGAATGTTTATTACTATGAAAATAACGTAAAA
TTTCAAAATCATGCACGGTGCAGTATTCACAAACACATATGAACTTCTATTTATAAGCAAGGATTTGAACATG
GTATAAACCTGAAAGTTTGTGAGGATGCTTGAGATATATCTCCAAACCAGATTTCATCACAAAAACTCGTACC
AATTAGGTAATATGGTTGAAATAATTAAATTTCTGTTAGATATCATTGAATTCCTTAAAAAATTGCATACATT
TAGGCGTTTCCCGCGTAATTCTTGAAATTTAACTATTTGTTATTTATAGAAGTATTGCATTGTTAAGAATTTT

ACAAGCATATTCGGATTCAGGGGCTCAAATTTATCATATAGAGTTGTTTTGAAAACTAACAATAATGGCATTG
ACAAGTGATCAATTTCACCCCGAAATGAGATCCCCTGATTTTTTATTTTAGAGATATTTGTTAACACTAAAAT
GACATTTGTTAGAAAATTTCGGTACATGAGTCGATGAGGCTCACCTTCGTACTTGTTTTCCTGCATTAAGTTG
TTTGGCATTGTTAACATTATAGAAAACAGACTAGAAAAACCGTGAAAAATGATCAATTCCACCCGAAATTACG
GTATTTTTTTTTTTTTTTTGAGAAATCTCGCAACGAATCCGTAGACGAATTGCTGGGGGAATCTCTGTAAGCG
TTCTTGGAAGAATTTCTGGCCTTACATGTGATCTAATGGTTAAGATTAGGTAATTTATGCAGGAAATATTAGA
TGTGTATACAAAGAAATTAATTTTATCAATCCGAGCTGAATTGTTAATCGGCGTCGTAGTGCTGACCGGTGTA
TCGCGCGCCACCGATACCTCACGCAGCGTTGGCGTGACGTAAAATTGATCGGCGGTGGCGCGGCGGCGCACAG
GTCTGGACAGTAGATATGAGAAATTAATGAAATATTTGAATTGATAATTATCCAAAAATTAAAAATATTTGGG
GGAATACTGTAAAAACTGAAACTCTATATGCAACATGTTACGGAATGAAGCTTCTGTGTTGAGTTAGCAGCAA
GTGAATGTAATTTTCTGCAAACAATCGTTCCAGTTTTCAGGATAGACTACGAAGTTTCTCCTCACAATTTCGA
CATTCGTACAGAAATGGCTATATCAACAGCATAATTAGTGTTCAATTTGCTTCAGTTGTTTTGTAAACCATCT
ATTTTCGATACCTGCATCTGTATAGATTGCAAATCGCAGTTCCAGCTGCTTAAATAATCAGTTTTCACTCAAT
CAACAGGTTCACTGGAATGAGCAACACCCACTCGAAACACCCTTTCTAGGTTTCGATACAATTTGATCCATAT
GGTACATCCATTTTTAGTCGTCCATGGGAGCCAATGCGTTTGAGACACTAGAAGTCCCAGAACTGAGCTCGTA
CTGTCGTTGCTATAAAATGATGACAAAGGTTTCTCGCTAACGCCTAGAGCAGCAGAGATACCGAAAAACAACC
GGCTTTTGTATAGGTAGGGTTTTTGTGTCAGGCCCATCACGCAATATCAAACATATGTTTACATGTGTGATCG
AAAACATAATTCTTTATCGTCTAACACTAGTTAGTTGTTATCAAAACTAAGGAATAAGTGTCATTTTGAAATG
ATTCCGTTTCGACAATGGCCGACAATAGCGCCCATTAACTCTGCCATACGTTAACAACTGTTCTGGATTTTAA
ATGGCAATCAGGTGGCGTAAAGGCACTTAATCATCCAAATTACCTCATCCGATCGATTTTATTCGATATTCCC
TTCCAGTAGAGTTCCCGAATCCAATCAAAACATCCCAATCAATCATCGCACCTTCTGCCCAGAACGAACCATA
TGTTCTCAATTACCGTGCGTCCATCACCATATGGAAGCCAACACGAGCTCTAGGTGCTGTTTGTCAAATTGCG
TCAAGAACTTTGTTTGAGCATGCTCCGGGAGTGCAAACAGATAACGAGTCCAAAATTCTACCCCAGTCGACGG
CGAAACCGTAACTGCTGTTATCTCCCCCGCGAGTCAACAGAACTCCCTGCCGGCCAGGTATGATATTTTCGTC
GTCGTCGACGGTAGATCTCTTATCAGTAGCTCTAGATACCTGTGTCGTATTGTTCACCGAAAAGTGTTCTGAA
CCTGCTGCAGCACCTCTAAACGAATTGAGCGGTTGGGGGAAGCGCACCTGAAGTTATCCGCACACTACACACA
CTCTTCAGCTCGTTATTTTCTGCCGGCCAACTGTCAGTGCGATTCTATCCGCATATCTCGTCGAACACTTTCA
CTTATCACAAATTACGGCAATTTTCGCGAACCACCAGTGTAACATATGTTTCACGCGGTTAGCTTTTTCGCTG
CCTTCGCTTGCACACGCTTCTCGCGGTTGGCACCACTTGTTGGATATCGCACTTCGACGGCAACCAAAGCCGC
GAACTTCGTCAAACCGATGGATGGATAGGTGGATGAATACGGATGGGTAACTCAGTATACTTCACAGCCGGAG
CTGGAACCAGAAAGGGATTTTTGCGTCCACTAACCAACACAGCGTCGTACGTACAGAACCAACCAAAGACGGT
CGAAAGCTATTCATCGAGAGCGACGAAACCCCCGAATGTTACGCTGCCTCGGTTTCCGCTCCTGGGCCCCGAC
GGATGAGAGGAGGGCAGGAAGCTTCTAGGTGGACGACTGCGGCGGCTGCAGGCAGCCAGATCCTTATCAAATC
GTATTCATTGGACCGCATTTGCTCACCGTTCTTTTGTGGGGCTAGAGCGAACTCTCCCTCTCTGCTGCGTACT
TAGGAGGTTCCTTCATCAGAGCCATCGGTTGTCACCGTGTTTCGTTCCTACGAAGAACGGGAGAATGCGTATC
GTTATCTATCAGTGGGTGATTATGGGGTGCTGGGTTGGTATAGGGTGGTTCGAATGGATGGCTATAAAAATCT
ATTCTTTGTTGAGATCCTGATGTACCAAGCTGATGGCAGACATTTACATATTTGTTCAGAAATCTTAGAACAT
GAACATGTTCTATCGTGCTGTGCGTACACAAATTTTTCCTGCTTTTGGAAGTTTTTAAACTACCAAAGGCAAA
CACTACTTCTCAGAGCTACAAAAAAGTACCTTTCAGTACTTCTTCTTCTTTTTGGCGTTACGTCCCCACTGGG
ACAGAGCCTGCTTCTCAGCTTAGTGTTTTTATGAGCACTTCCACAGTTATTAACTGAGAGCTTTCTGTGCCAA
TTGATTATTTTTGCATGTGTATATCGTGTGGCAGGGTACGAAGATACTCTATGCCCTGGGAAGTCGAGAAATT
TTCCTTTACGAAAAGATCCTCGACCGGTGGGATTCGAACCCACGACTCTCAGCTTGGTCTTGCTGAATAGCTG
CGCGTTTACCGCTACGGCTATCTGGGCCCTTTCAGTACTATTTTTTCAACTATTGATCCTTGTTTGGACCCGT
GCCTTCGATTTTTCGTTGGACCCGTAAGCTAGCCACGGTAATCCTTTTTGGACTCCGTCTTGAAAAAAAAACAA
CCTCTTAGAAGATCACACACATCTAGTAGAGAAGAAGAACATTTTTTGCTGATGACGACAAAATTGAACGTTT
GTTCAAGGTCGTCTTGAAAGGTCTGTCAAGTGACTTTGAAGCCTTGATCACAATCCGCGCGCAATGCACGAAC
GTTCATTAAAAAAATCGTTCAATATTGGTATATAATCGTTCAAGAATCCAAGTATATTATTAACAAACGATTT
CCCTCAACTAATTCTTATTCACTTCCAGATATTATTTTAAAATTCTCCTATGAATTTCTTCAAAGACTATTCC
AAACATCAATGCAAGAATATTAAGAATTGTCTTCAGGAATGACTCAATACAAATGTCGAGGAAATCCTTTGGA
AATTTCGTCAGAAACTCAAGAACTTTATGATTTTTGGAAATTCTTTCAGGATATCGTTCATTGAATCCCTGGA
TAAGTTCCTAGAGGGATTCCTGGAGTAATCGGAGGAACGTCTTGAAAAAATCTAGAGGAATCCTCGGTGAATT
CCTGAAGAAATCCCTGGAACATAAAACATACTTTTTGGCTACGCAGTCTCTGTCAGCTCATTCCTGGAACAAT
TTCTAGAAGAAACCATTATAAATTTTCTGGAGCCTCGGAGACCTTCCCGAGAAAATGGAAGAATCTCTGCAGG
AATTCCTGTAGAGATTTTCCAACAGTAATCCCTAGAGGAGGTTCTGGAGAAACTCCTGTAGGAAGGATTATCT

AAGTTAATTATTTACTCCGGGAGCTATATTTGGAGGAATCTCTAGAGGAGCGGTTCCACAGAAAATGACGACT
TTTTTCCCAAATTTGATTTTCATATTTTTTGATTTGGATGAAATTTTGCACATGCTTTCTTTATGCCCAAAAA
TGCCTTTTTGCATCATCGGCTCGCCATTTTGACTCTAGCCTTACTTTTAAGAAGGGCCTAAGAAAAAAAAATC
CTTAATAATTTTCAAAAAAATATAACTTAGAAACGGTTTGTCCGATCAGTTTGGTGCCTTCCGCATAGTTTTA
GGTTATTGTTAGGACTATCTGGAAAAAAATATACACTGTAAAAAAAAATGTTGTAAATTTTTATATATCGAAA
ATAAAGCTTAAAAATCAATTTTCTCAAAAATCGTATTTTTGTTTTTATTTTTTTACTAGTGGTCCCGGCAAAC
TTCGTTTTGCCATCAAGTAGGCTGTTGGAAAACGTCGAGAAATTCCCCATACAAATTACACCTTAGTCTTCTC
CCGTTTTCCCGATAAACCCGGCGACTTTCCCAAATTTTTTCTTCGCACGAACACGTCGCATCCCTTGAGGGGA
GCAACAGTGAAAATCTTGCGGTAATCCGTTGGACCGTTCTCAATCCATTTCGTGACATACAAACACCACTCCA
TTTTTATTTATATAGATATGTTAAAGTAGACAAAAAATGAAGTCTTTTGCACAGTGGGTCAATATGGAGAAAT
CATGGACAAAAAAGTTATGATTTTTTGAAAATGGTCATAATAAACTTTTTAAATATTTTTAAACTCGATTTTA
TGAAAGTACGCAAAATTTACAACGAAAAAGGTATATGAAAAAATTTGCTAATTGCTACCGAAACATTTGAAAA
GTTTATTATGACCATTTTCAACAAATTCACCTTTTTTTTAAAATCATAACTTTTTGTCCATGATTTCTCCATC
CTGACCCACTGTGCTAAAGACTTCATTTTTTGTCTACTTAAATATATAAAAAAATTAATAAAAAAATCAAAAAT
ACGATTTTTGAGAAAATTGATTTTTAAGCTTCATTTTCGATATATAAAAAAATACAACATTTTTTTTTACAGT
GTATATTTTTTTCCAGATAGTCCCAACAATAACCTAAAACTTTGCGGAAGACTCCAAACCGATCGGACAAACC
GTTTCTAAGTTATATTATACAGTGATACCTCCATGAGTCGATGTTCCATGACTCGATATCGACTCATGGAACC
ATACTAAAAACAAAATATCATGGTTACTATGATGGACCCTAGAAGCAGCTTTCCAAAAGATTGCTGTTCCATG
ACTCGATATTTCCATGAGTCGATGGTCCCTTCAATATCGACTCATGGAGGTTTCACTGTAATGACCGAAACTT
GAAGATTATATCATAATTTTGTATTAAAATCGCTGTATCTTTAAAACGTTAAAAGTTAGCCTGATTTTTCCGT
ATACCTTTTTTGTTGTAAATTTTGCGTACTTTCATAAAATCGAGTTGAAAAATATTTAAAAAGTTTATTATGA
CCATTTTCAAAAATTCCCCTATTTTCAAAAAATCATAACTTTTTTGTCCATGATTTCTCCATCTTGACCCACT
GTACAAAAGACTTCATTTTTTGTCTACTTTAACATATAAAAAAATAAAAAAAAATAAAAAATACGATTTTTGA
GGTATTGATTTTTAAGCTTTATTTTCGATATATAAAAAATTACAACATTTTTTTTACAGTGTATATTTTTTTC
CAGATAGTCCCAACAATAACCTAAAACTTTGCGGAAGGCACCAAACTGATCGGACAAACCGTTTCTAAGTTAT
AATTTTTTTGAAAATTATTAAGGATTTTTTTCTTAGGCCCTTCTCAAAAGTGAGGCTAGAGTCAAAATGGCGA
GCCGATGATGCAAAAAGGCATTTTTGGGCATAAAGAAAGCATGTGCAAAATTTCATCCAAATCAAAAAATACA
AAAGTAAACCGACATTCGAATTCAAATGGAACCGCTCAGAGAATCCTTGGAGGTAGCTCAGGAAGCATCACAG
TAGAGATTTTGCTGGTGGAGTCCCTAAAGAAATCCCGGGAAAACCTATAAGCTGTAACCTGTAGAGTATTTCT
GAAGAAAGCCCTGGAATTATTCTTAGAGGAATCCATTGAGAAATGTTTGTAGAATCCCTAGTTAAATCTTCGG
AGAGATCTATGAAGAGATTTTCCAAGAGGAATGGGGGAAGAGCACCAATTTTCGACCCATTCATACGATTTTG
GCATACTTTGTATGGAAATCCGAAAATTGGCGTATATTTCTTGTAGGTCAAAAATTAGTGCTTTTCCCCTAGA
TGTGCAGATATGTTTGGAGGAATTTTTGTTTATACACTGATAGGCAAAATAAAGTGCCCACCTTACCAGTTTT
CGAATTTCTTTCATTGATTTGGTTCAAATTAAAGTTAACACACCCCAGACAACCAGAAATCGCATGAAAGTTC
ACGTTATAACTCGTTTATTCATACTAGTTAAATCAAACTCGCAAAACACCGTGGATAAACTCGTCAAATTGAT
GAGTTTCCTCGCATAAAACACTTTTTTCTGAGTTTATTTGTACATTTTGTTTCGTCCCGTACACTCGTACAAA
GTAAGTCGAATCAGTCCGTAGCAGCTGTCAAATAAACATTTGTTATCATAAGTTAAGTCGCATAAGATAACAG
GTTAGTTCGGTAGAATATTTATACACTCGCATTGTATGTTATTATTCATCACATAATATGTGCACGCATATCG
CCTCCACTTTTGTACGTAGAAGGCCTTTTCGCGACATCTTATAAGTGAAATTTTGTATGTACAAAGCCTCCAG
GTGATTTAGTTAGGCGTACATTTTGGTTGTCTGGGACTTAAACCTTTTTTGATATTTTATTTTTAATATTCTT
TTAAAGTTGCACTTATATTGCCCACTTCCTTCTTGGCCCCAGAAAAATGATTTTAGAAAAATAATAAGCAATT
TATTAGTTAATGTGTCCTCCTTTGGCCTTAAGGACTTGCTGGAGGCGCTTCGGCATGCTTTTCACCAGGTTTT
GTAGGTGTTGTGGAACTAGTTCTTTCCAGGCGCGCTCCAACCCAGTAAACACACGATCGTATATAATATGATA
TAAGAGTACAAATGTGGAGGCGATATACGTACATTTTGCAGGCAGCCTAATGGCACATGTACGTATATCGCCT
CCACTTTTGTACTCATATACGCTATCGTATACGAGTTTGTGTTTACTGGGAAGGCTTCAAAATAATTATTTTT
GTTGGTAACACCAGTTTTGTCAACCCTGGCATCGAGAATCGCCCACAAATTCTCGATGGGGTTGAGGTCTGGG
CTTTGTGGAGGCCATTCCAGTGGTCTAATCCGACAACACCGGAAGAAAGACTTGGTCTTCTTGGCAGTATGTT
TCGGGTCGTTGTTCTAGAGAAATATGTATTTCTCTTCAAGGCCCGTTTGGATCAGCGAAACCTCCAGATTTTT
CCGCAAGATGTTAATATAGGAATCTGCCATCATTATTCCGTCGATTTTCAAGAGGCTTCCTACTCTACTCCAT
GAAAAACACCCCCAGACCATCACATTTCCTCCTCCATGCTTCACCGTTCCTTGCCCACACAACCAAAATGTAC
ATATAACAAAATCACCTTTTGCGTTATGCGATGCTTATATGCGCAATGTTTCACGTATAAGATGTCGCGAAAA
GGCCTTATACGTACAAAAGTGGAGGCGATATACGTGCATATTTTATATGGTGAAAAGTAGTATGCAATGCGAG
TGTGTATATTTTATTGCGAACTAATCTGTACTCTTATACGACTTAATTTGTAACAACAAAGTTGATTTGACAG
CTGCTACGGATTGATCCGACTTACTATGTATGTGTATGCGGGACGAAACGAAATGTACATATGAACCCATAAA

AAAGCATTTTATGCGAGAAAACTCGTCGGTCAAATGATTTCGCACACCATTTAGTGCGGGTGTGATGCAATTT
GTACTAATAAACGACTTTGTCCGTAAACTGTTATGCGACTTCTGGTTGTCTGGGTGGACGTGGCGCTCTGCAC
CACACACGAGCCCGCCGCTTTCGGTTAAACAGCTCGAGCTTCAGGAGCGCCACATCCAAGGAACGGTGACGCA
TGGAGGTGTAATGTGATGAAATGTGATGGTCTGGGGGTGTTTTTCATGGAGTGGAGTAGGAAGCCTCGTGAAA
CTCGACGGAATAATGACCGCAGATTCCTACATCAACATCTTGCGAGAAAATCTGGAGGTTTCGCTGATCCAGA
CGGGCCTTGAAGAAAAAATCATATTTCTCTAGAACAACGTAAAACTCTTTTTTTTACTAAATTTTTGTAAGTG
CAACTTTAAAAAAATATCAAAAATAAAATATCAAACAAAGTTTAAGTGTGTTAACTTTAATTTGAACCAAATC
AATAAGAGAAATTCGAAAACTGGTAAGGTGGGCACTTTATTTTGCCTATCAGTGTATTCGCCTAATGTAATTT
CCGGAAAAAAATGTTGGGATAATTTCTAATGGAATCTTGGGAAAAATTCCAAGAGGAACTTTTAGAGGAATCTC
TGAATACATTTTTGGAAGCCCAGAACTAATCTCTCGAGAAATCCCAGGGGAATCCCTATAGGATTATCTGGAG
AGATCCTTGGAGTAAGCCATACAGCGATTGTCCTAGAAGTAACTATGGTAGAATTCATGAATGAAATTCTTGA
GGAATTCGTAAGTTGCAGAACACCTGTAGGAATTCCTGAAAAAAATATCTGGAGCAAGTCGTGGAAGAATCCT
GGCAAAAATCTTCGGAATAATTCCTGGATGAATCTCGAGATGACTACTTGGGGATATCTTTGTATGTATCCGT
GGAGGAATCCTCGGAGGAAACTCTGGAGAAATCACTGTAGAAATAACTTGAGTAATTGTTGAATAGATTATCC
TAGTGTACCCCTGGAACAATACCTGGAGAATGCGTTGAAGTTACCTTTGCAAGAATCTTTGTAGAGATTTCCC
TAGAAGAACCCTTGGAGAAGTCTTACAAAGAAAAACCACTTGAAAATCCCTGGATAAGTCTCCATAAAAATAA
TCCGGAGTATTAGAGGAATCCTTGGAGAAATTCCTGAACGAATCCCAGCAAAGATGTTACTGGAGGAACCTAT
AAAGGAATTTCTGAAACATTCCTGCAAGAACTTCTTGCGAAATCTCTTAATAAAGGAATCCCTGTAGAAATGT
AAAATATCTAATGGCAAAATCTCTGGCAAAAGCCCTGATGGAATCCCTGGATAAATCGCTATTGATATACGGA
AGAGATATCTTGAAAAGATATCGTTTTGCGGAGCAACATAAACTGCACCCTGCTGCCGAGCTTCCAGCTGCAA
GTCATTGAGGCCAATCTGGTGGGAACCTCCATCGGCCCAATCAAAATCATTGCGGCGTACTGCTCGAAGCAAG
CTTCCATCAACGATGGAACATCGGTGGCCCTAAAACGGCGGCATGAGCAGTTCATCCTGTCTGGCGCAAGGCA
CGAGACATGGGGAAACACCCGGCGAAACAGAAATGGCCTCATGCTACAACAGGACCTGGAGGAAGGCCACTGC
ACCATCCTGAGCTCAGATTCGCCCACTCACCTGAGCCGGTCCGGGGCCCACTCAGCCTTCGATGTCTGCCTAC
GTTAAAAAGCGAAGTTAATCGCATATCACAAATCGTCATGGAAAGAATAGAGGACCTTAAAAATGAAGATTTT
TATAACAAGATCCGTTCTCTCCCAGATTGTTCTACACTGAAAAAAATATTTAATTTTCAATGTGATGTGAACT
GAAGACTACTGTAAAAATAAATCAAATTCGTGTGTTGTTACAGCAAATTTAAATGAATATACATTTAATTTTC
AACTAAACATGATAGAATATTACATGATCGTGTAAATTTAAAGTGAATTCGATTGAAAAATAACGGATTGGTC
GTTGAAATTTAAGTTTATTTTGATGCTCCAAATATGTGCATGAAAATAAACTTAATTTTACAACATATTTTTA
GCTGCGTAGACCATTCTGGAAGATGACCAAAATTTTACAAACCAAACCCAGGCCAATTCCACCGTTGATCCCA
CTAGACAATGCTGACTCTTAAGGATCACCTGATAACCCCTGTGGAGAAGACTGCTGAGATAGGTCGGCATTTC
ATCAGCTCACACAATCAGTGTTGGGAAAAAATCTGAAATTCACTCTACAGTAGTCAAACAAAGGAAATCGCAG
TCAGCGAAGCCAGTGAAACTCACGCCTACCGCTGCTGTAGGCAAAGAGCCTTGAAAATCGCAAAACACCCGTT
GCTAAGGGCAACCCAAAATTACTGAAGAAAAATGTTCTATTTCCCGCTGCATTTCCCGCATTTAGAACCTTCA
ATGACACTACTGATTTAGAGAAAATCATGTACCCAATTTAGACTACTTGATGAGGTTCACGATTTTAAACTAC
TGTAGTGAGAGAGCCACGTGATTTTCGCGAAATTCAAGCCTACTACTATTACCATTACCAGCACTGCACACAA
TCTATGGCTAGACATTCCTAGCCCACACGAAGCTGCTGTCTCTGAACACGCAGCTCACCTACACCGATCTCCC
AACGACTTCACGGAGGAGTTGGAGATCACAGCTGACGAGTTGCTGGCCTATGTCAAAACATCTAAAAACATGA
CGGCCCCAGAATTCGACAATATCCTGAACTTGGAGCTCAAGCAATTGAGTCTCCAGTTCTATCTACACTTTCA
CTGATTTTCAATCAGTGTCTCCGACTTAGCTACTTCCCCTCGTCGTGGAAGTCAGTGAGTCATCCCCATCAGG
AAACCTGGGAAAGATCTTTGTCCCTCCAAAAGCTATCGCCCCATCAGCCTTCTTTCAGGGTTATCAGAGTTTT
TCAAAAAATGCGATCAACAGACGGCTGCTTTCGGCAGCCGTTCAAAATAACATCCTACTCGAGGAAAGGTTCG
GCTTTCGACGCGGACGATCAACCGCGCACCAACTGACTCGAGTAACCAACATCCTCAGGCGGAACAAGCCCCT
CACCAAATCCTCTGCCATGGCGTTACTCGATGTTGAAAAAGCATTCGAAAACGTCTGGCATGACGGCCTGGTG
TACAAGCTGCACCGATACAATCTTCCCACCTATTTGGTGAAAATCATCAAAAACTATCTATTTCATAGGGCGT
TCAGAGTTTCCCTCAACTCAACTCAGATCCACTCAAAGTCCACAGGAAAGTGCTTTAGGTCCCCTACTATTTA
TCCTGTTCACCTCGGACATGCCTCAGCTCCCTGAAGGCGGCTCTCTATCGCTGTTTGCTGACGACACCTTCGG
TATCAGAAGGCCGGCTCCAAAGGCACGTTCCCCACCAGTTGGGAGATTTGTGCCATCGCCATATTTGAGCCTA
TTTCATCATCTGCCCGATGGGAGAGGAAAGGGAAGGGAAAGATGGGAGGAAATAAGAGTGGGGTCCCTTGAAG
AGGGAAGATCGCATAAGCGAAATGAGAGCATGTAGCTCCATCACGGTGCTCCCCTGACCGTTATTATCAGAAA
AAAATCTCCATCAAATCTGTACTCACTATTCGATTTCTATAGCTAAGCTGCATGTCTGGGCAAAATTTTAAAA
AAATCGTAGGGCCCGTTTTGTAGTTACGCCCTTTTGAATGTATAAGCCCACTAATTCAAAGGAAATCTGAGAT
ATTTAAACGAGTTTTCATTGGCCGTGATTATCAGAATAAATATATTATCAAAATTTGAATCAAAGTTGGTTTA
TATAGTTATTTGTAACTTCTAGGAGGAGTTTTGAATGAGAAGATAAAAAAAAATTAGGGTTGCGCCCTTTTGG

GAGCGTAACCATTGAAAACACTAATTTCCAATAGATTAGTTAAGCATTCTTATACCTTTGAGCATATTCAAAT
GTTTTTAATGGCACATTGCACTTATATGCCGCCAAAGTATTTCACAATCAACCAATAGGCTCAAGTGTTTTAA
AGCATTACGGAGCCAATTTAATCATGAAACCGAATTAGTTCTTGTAGCACAATATTTTGGCTAGTTTGGTGAA
CCCATGTACTCACGTTTCAGATTATTTTACACGCAATTGATTGCTACCTGTTGTATATCGCAGCGAAGCATAC
GTCAAAAATATTTTACGAGTTATGTTTTCGTTTATGGGTAGCAGTCTATGGAATTAAATAAAATTAACAACGC
AGAAAGCAGCGATATCAAATGGAAGAAAACAATGTACATCCGTTATTATTTTGTTTAATGGAGTTCAAAATCG
TAGCATTAACAGAGCCTAATTTTAAATGTTTGTCTCTGTCAGTTTGAAAAGCTTTGTGAAGCTCCCTAAGATT
TTTTTCGTTTGTGATGAGTCATAGCAGTTTGTGAGTCTTTACTTCACAAGCATAGTCTGCGATCGAAAAACAA
ATACAAGTTTTCATGGAAATCTTCAGTTTGTGTAGTAATAAAACCATATTCTGGTAGGTAGACCAGCAGGTTA
TTAACAGGGCCACAGAACAATTTACGATTTTCTGGTAACCAAAACTGGAATTCACGTAAAATCCATGAACAAC
TCTGTTTTTACCATATTTTATATGTCTTCTTAAAATTATATCCCTTAACTTTCAATTCCATTACCCCGAATAC
CTGAAGACCATCTCCGCATGTTCCAGTTTCTATAATGGCATTTGTTCGAATTCCATTACTACCCGAGCAGGAA
CAAATAACTCCCCAATAACTTATGCATACCATATTTTGGTATCATACCAAAATTAGATATTGTTTAGTTGTCA
ATACCTCAATTTGGTATTATAATGGTATTAAAAAAAATCTTTAAAACGATTAAAAATACTTCATTGAGGTATT
ACACAGCTATTGAGGTCTGCTGGAGGTATTGAACTACTATTGAAAAATTTCACTTTTATATGAAAATCCATCA
AATATTGTTTAGGTATTGCAATACTTGATCTAATTATCAGCTAGGTATTTGTAGAACATGTAGAAGGTATTAT
TTAAGGTATTTTTCTTAAGCACATTTCAAGTACTGAGGCTTTTTCTTCACGAGTTAGCATGAGAATGGATTGA
TTGCTTTATACAAATCTTGCACTATTGTATTTTTCCAGAGTTCGTACGAGTTTGTGTAATCGAAAAGACCGAG
AAACCCAAAGGTAAAATTGTAGCTTTGTGAAAAATCTAAATGTTGTAATGTATGCTACAAATGATTACAAAAG
TATTGACAGTGGTCTAGAGTGCTATAGTTTGGCCAATAGTGCATTACTCAGCAATTGAAGTTTATGTTCTGGG
GCAGTACAAAATTAGCCGGAATAGCCGGCACTACGTCCCCACTAAGACAGAGCCTGCTTCTCAGCACAGTGTC
CAATGAGCACTTCCACAGTTATTAACTGAGAGATTCCTTTGACAAACGTGCCTATTCTGCGCGGCGTGTGAGG
TGACACGAGTCGAATGAGGTGACAATTGTCGACTCGCTCTCATTTGATTAACATTAGTCGTCTCACGTCACTC
GCGGTGAGAGCGAGTCGTCTCGACTCACAAGCCGCGCAGAATAAGCACGAAAGTTGCCATTTTCGCATTCGTG
TATCGTGTGGCAGGAACGAACATACTCTATGCCTCGGGAAGGTTAGGAGGTGTGTAAGACTTTGGCGACATGT
AAGTGCAATGTGTCATTGAAAATATTTGAACATGCTTAACGGAATGCCTGCTTAAAAGAATGCCTTATTAATC
TACTGGAAATCAGTGTTTTCAATGGTTACGCTTCCAAGAGGACGTAACTCCAAATTTCGTCTATCTTTTCATT
CAAAACTCCTCCAATAGGTTACAAATAACTAAATAAACCAACTTTGATTCAAATTTCGGTGGTATATTTCATC
TGATAATCACTGCCAATCAAAACCCGTTTAAATATCTCAGATTTCCTTTGAATTAGTGGTCTTAGACAATAAA
AAGGGCGTAACTTCAAAACGGGCCCTACGATTTTTTTAAAATTTTGCCCAGACATGCAGCGTAGCTATAGAAA
TCGACTGGTGAGCACAGATTTGATGGAGATTTTTTTCTGATAATAACGGTCAGGGGAGCACCGTGTCCATACC
ACAATGGGTACGAACAGCGCCCTAAAAAGGGCACTGCAAAACGCATGAGCGCAAAGAGAGCCTATAGCTCATT
ACCACAGCGGGTTAAGAATAACAGAATGTCCTGATGATTCAGGCTTCTGAATTCAGTTTCACTTAATAAGTGT
TCACCAAGTAATTGGAATCGCATTTGCACAAAAACTGGACAGTTACATATCAGGTGATACGAAGTTCCATAAT
CGGAGTCACAACTATCAAACAAAAGAGTCAGCACGCTGAATGTTTGCTATTTGATAGTTGAGTCGGCATTGGC
CTGTCAACGCTCTGACCAAGAGACTGCAATTCTGCTTTGACAGATTTGTTAAATATTCCGCCATCTCCAAGCT
CAGCAATGTACAATTTTGTTTGACATGACTCCAAACTATTCCAATATTGCTTGTGCTGAGTTGCCGCCCAAGA
GTTTATCTGAAGCTTCACCCAGCACTTCGAAATTGGAATAGTCGGCTCAGGGCCAATGAAGTCATGCGATGCT
CCATCGCGAGCTAGCTCATCGGCCAATTCATTTCCAGCGATGGAAGAATGGCCAGGTACCCATACAAGGTTTA
CAGAGTTGACTGAATTCAGTTCCTCAATTTGAGTTCGACATGCGATAACAAGCTTCGACCTTGAGTTGTCCGA
AGCAGGGGCTTTTATAGCACTCTTGTAGTCTTTACAGACCGCAGAGGACATGCTTCTTCAAAAACTTCCATAA
TGTAGGATGTTGTAGTATCAACGGCATCATCCAGATCACTTGGATTTTCAATGGACGGAGAATATCCATGAAA
TTAGGTCGCAACCAATTCAATATAGAGTTCCCAGTTTGTTGACTGGGGATTCCTAAAACGCAAAGTCTGCGCA
GTTACATTTGAATGATCAAAGAAGATGTAGTGATGATCAGATAATGATTCCTCATCGGATACATGCCAATTCG
TCAACTCGTGACTGATTCTATTCGAGCAGAGCGTTATGTCTAATACTTCTTCTCTATTAGAAACCATGAAGGT
TGGGCGATTGCTAAGTAATCCAAGGTCTGTACTACGATTGTTAAGTAATCCAAGGTCTGTACTACGTAAGTAT
TCCATCAGACTGGAGCCTCTCAAATTGATATCTGAGCTGCCTCAGATGATGTGATCAGCGCGAGTTTGCCATT
TCAAGTTTGCTGAAAGTAGCAAAAACTGGGTCCACAAGGTTACCTAGATAGAAGTTTCCTCTACGAAAGTAGG
GTTCTTGTACTAGCGCCATTTGGGCTGCACCATTTTGCATGAGTCTGCAAAGATTGATCGTTGCTGTTCTTTT
ATGCTGAAGATTGATCTGAGCTAACCTAACCGTAGCCACTACCCAAACTAGGCAGGATTAAGCTTTTTGCAAC
CTCAGCACGAAAAAGACCAGCAACGAAAAAACCATACCGCCAAAGGCGAAAGAGCACAGAGTACACTGTGTAA
AACGCATAATGCGAATCCATATAGGTGATAATTTAAATTTAATGTCAACATATTCATAATCCCACCCTTATTA
AGCCTCAGGATAGAGACTGAAGAAGGGCAGCCGATTATCTCGGAGAAACACAAGGTCACCTGCACCATTGCTC
CGGGTAGCACAGGAAGGACTCAATACTGCGGAGGGCGCCCTGGTACCCCACAGGCTCCGTTTGCGGTTAGGTT

TTATTTAGACCCCCCTAACCATTCATTCCTAGGCACGGTACGCATCAAACCATAAATTAGGGGTCACCTGTGA
GGTGGACTTTTAGCACCGGAACAGGCAGTCCGTAGTGTTAATTCTTAGCCAGTTGAAACAACCGCTACCGACA
CTACGCGGCTATCTAGGCTGCTCGGGAAAAGGAGGTTAATATTGATGATTAACTCCTGACGTGCCCAAGCAGC
CGTGTTTTAGGTCCTCTACTATTTATCCTGTTCACCTCGGACATGCCTCAGCTCCCTGAAGGCGGCTCTCTAT
CGCTGTTTGTTGACGACACCTTAGTCGTCTACTCACTCATTGAGCGCTTTGGTGACTCGATCTATAGCGATCT
ATGGCTCGCTGACAACAATCAGGCCAGCAGGTCATCCAGGACCTCGTTTTCCTCTAGGTTATCAAAATTTGTA
GGCTTGCAAAAAGTAGTTAGAATAACAAATTGTACTTTTTAAAAACTACCAGAGCCCATTAGGCTAAACAAAT
TACTAGGGTAAAACCAATCCAGAAATTCAACCATAATAAAAACAACCAACAAAGCTGAAGGACCTCTCGGTCA
ATCTCTTAAATGCTAAAAATAGTGACCATCTAAAATTAAATGTAAATAAAAATGAATTTAATAAAATGAATGA
TGCGCAACGGAGTCAGTTTTTGTTCACCCGTCGGCATGAAGTCAAGCGTCATCCAGCCGATGCCGATTTGGCG
AACTGTGATCGTCGCGAGGCCTTTTCGCAACATCTTCTTATTGTTTTTGGCATTACGTCCTCACTGGGACAG
AGCCTGCTTCTCAGCTTAGTGTTCAATGAGCACTTCCACAGTTATTAAATGAGAGCTTTTCTTAGCCAAATTC
GCCATTTTCGCATTCGTATATCGTGTGGCAGGTACGATAATACTTTATGCCCAGGGAAGTCAAGGATATTTCC
ATTACGAAAAGATCCTGGACCGATCGAGAATCGAACCCAGACACCTTCAGCATGGCTGTGTTTGGTAGCCGCG
AACTCTATCCATTCAGCTAAGGAAGGCCCCAATGCATCCTGATACGATTTCTTATATTTGACAAAAATATTAT
TATGCAACTTGTTGCATAAACTACCATTTCCGTACCAAAAGAAACAGTGCTGAAAAGCAACTCTGTGAGCCTC
GTTGAGTCAATGTACGAAACCGGATGAGAAAATCATTATTTTGCAACTCGTTGCAATCCCAATCCAAATGATG
GGACCACCCTCAGATAGCAAAACGACACGCATGTGCCTTTCTGGGCGTCCTTCCAAGGATCAAATCTGGAAAA
CAGATTCGAATGCAGAGGCATACGCACACACACATATATATTACCCTCCGTAAGGAAGCGTCAAGCACATTTC
ACGACATCAGAATTTGCCTCGACAGTAAGACTGGCCAGCTACCAGACGACGACGGGTGGTCGGAAAACGCGCA
TTTCCAAAATACTTGTTGCTACTTCTCACGAAAAGAAATTTCCTCTCTCCATTGTCGCTCTCGCTGGAGTGTA
TATTTATATCTACCGTTGTGGTTTGGTTTTTGGGCAAAATATGATGGAATGCTTTGGAAATTATAAAGTTATT
ACGATCATATTGGTATATTTTGTACTTAGAACTTATCATTTCCAGATGGTAAAACGGCAGAATTATATCAGCC
TACTTCTCGTTACCTTAGATTAAAATATCCTGTTTTATAGTAGTGCATTAGCAATCTTAACCGAATATGCGAA
AAAATTTCAAAGAACCGAATTAATCATATAGCATTTAAATTCGTCCTGCGCTAATATAGTACTATTATTCAGC
TGACAAGCCCTATATTAGCAGTATAATTGCCGTATAAGCCCAAAAAGGCAAGAATTAAAGGACAAGTGGTTAC
TCGAGTGGTTCTATTTTTTGTGCCAATTCTGTTTGGAAAGCACATGCTGACAAGTATTCGGAGAGAGATTCAA
ATGAACAGGAGAATACCTTCGATGTGCGAGAATAGTACTCTCGCAAGCCAATCGCTGCGAGAGATGATAAGCG
TGAAAATACTTTCAAGGGGAATCCGGTCGTTGAGCGGCGGAACGAGGAAAAGTGCGGAACGTGCCGGGAAAAT
CTCGACAAAACAAAATTCTAACATATGTTTTCGCTAGAATTTGACTTCCAAGCCGGTGGCAGTTTCTAGCGCG
GGCCTCGTTTGTAACTCTGTGCTCCTGTGGATGAGAAATTCTGGTCCGAAAATAGAACGCACCCACTGACCGT
CACCGTCGTTGTCGAACGGAATAAACCAGACCAGTATGTGATGACAGGGACTGCAGCACAACTTCAAACGAAC
ATATGGAATAAAATGTGCACCACCCGCAACTGGCGGACTGTTGACACTGGGCATGACGAGAAGAAAATAGAAC
GACTAGAATATAGAAATCTAATCACCAAGTTTGTTCCGTGGTTCAAAATCATTTGAGTGAGAGAATTTACATT
AACACTAACTCAGGATATCTCGAAAGATTCAACTGATTAATTATACTGTAAATACATATCGGCCACAACATTT
CGAAAGGATGTAATTGCTTTTATAAAAAAAACCTTTTCACCTTGAAGCGTATTGAAGCTCATCCCTAGTGCTG
CTGCTATTAGAAACAGAAAATTATCATCTCTCTGGTATTAGGGTTGGATAACGTAGGAGATCTTCAGTTTCGA
TTAAAAGTCTTGTATTTGTGTAGCAAGGATGTCCTTTTGAAATATTGTAGCAAATATGTTGCAATTGATTGTA
GCACAAATATAATTTAACCTACTCATGAAGACTGAAAGTGATGTGATTTGAATGTATGCTTTCAGGGCCGATA
TTTTTTGAAAAATATTTCAAACAGCCGTAAGCCAGAAGAGTAAACAAACCTAGTTTATCGATCCTACGTGTTT
CGCAGACCAAATTCTACACTCTTCACCTAGAACCAACTCTATATTCACTTTTAGAACGTTTAACATTCTAAAT
TACAAAACGACTTAAGTGAGTTTCAACTTTTGTAACCTACATACATACATACACTTTCCAACTCGATTTTCAC
AATTACAGTCGCCTCTCCACATCTCGATATCGAAGGGACCATCGAGATAGGGAGAGATCGAGACATAGAACAA
TTTTTTGATAAGTACTAGATTGAAAAACACTTCGTTGCCAAAAAAGTAATACACAAACAGACGTCATTTTGCG
CTCCTAATTTGTTTTGAATCCCAAAATTTTGATCTAGTAACCTTTGATGATGGGCATATCGACATACGGAGAG
AAAATTGAGAACGAAAAATCAACAGAAACAAATCGAGATATGGAGATATCGAGATCAGGAGAAGGGACTTATG
AAATCATCGACATAGGGAGAGATATCTAGATGTAGAAAATCGAGATGTGGAGAGTCGACTGTATAATGTTTAA
CTTCTTGAATTACAAAAGGATTTAAGTAAGATTTACAACTTTCGCAACCTAACCACCACCTTCACGCCCTGAT
ATTTTCAACGGCTGCTTGGGCACGCCAGGAGTTAATCATCAATATTAGCTTCCTTTGCACAAGCAGCCTATAT
AGTCGCGTAGTGTCGGTAGCGATTGCTTCAATTGGCTAAGAATTAACAATACGGACTGTCTGTTCCGGTGGTA
AAAGTCCACCTCACAGGTGACCCCTAATTGCCCCCATGCGGCTTCAAGCTTACCGTGCCTGAGAATGAATAGT
TAGGGGGGTCTAAATGAAACCTAACCGAATACGGAGCCTGTGGAGTAGACCTATTCATATTTTAGAAAATGTC
TGGAAATTTCCCAGTCAACCAATATTTCGATTTTTCATGTCAAAATGAACTTCTGGTCAAAATTTCAGTCAAT
TTGGAGAAAATTTAGGTGTGCTTCAAATCAAATGTTTTTGGGCTAATTTCAAGCTTTAAAAATTCATAACTAC

CAAACGGAACAACGAAAATAATCGAAAATACCTCTACATAGTAGTTGATTCAATTCTACGAACTTTTGCCGAA
CACAATTTTATGATTGGAGCAAGTTTTAACATAGTTTGGTTGAGGTTTGCGCTTCGAGGCTCTTGAAAATCAC
CAATTTTGGGGAGTGTTCTCTCAGAAAAACATAACTGCATGAAAATTTTTGATTTTTAATTTTCAGAACATGA
TAACTTTACACATCTAAAACTCAATCACGTTTAAAGTTACAGTTTATCTAACGAAAATAAATTGCAAAAAAGA
GAAATTAGGAAGCACATTTGTAAATTCGCTGTGGGTGAGCCAAGAGAACCACCGTGAGCTTTGAAAACATTAA
AAAATGAACACGTTAGCACTGCTTTTTCTCAGTCGATGGTGATGATTGTTTTCAAATCGTCCTGAATCGTCAA
TGAAATGCGATCAAAACAATCATCACTCGGAAAGAAAAAGCAATGCTAACTTGTTCAATTCATTCATTGGTTG
GTATATGTGTCATGCGTGTATTAACTATCATCAGGTTGCGATGCGGTGTTCGATCTTTGGGCATTTTCTGTAA
TTTATTGCCTCAAGCAACATGTAAGCTGTTGATGGTTAATGAATTCATAGATTTATTACTTCCGATTCCCGTT
TACTAGGGCCTGAAAAACTAATGTCAATGTGGGTTTATTAAACCATATATATTCAGAAAACTGTGATTTCTGG
GTACAATGGAGAACAAATCTCGCTCAAACAATGTTAAAACTCAATTTGATCTCAATAGCGTGTTCGACAAAAT
TCAACAGCATAGATTCCCGACCAGAACCACATAACAAAATTATAACACATTCCTATCAGCAGCAGTTATAAAT
AACAGAAGTTGATTAAATGTTGTTGTCAAGATAGCTTTGTTCTCAACTTGTTATATTTTTAGTAACACATTTA
TAACAACATTTGTTATATTTTCGACATCGCATTTATAAGTTTGTTGCAAATAACATCAGATCCCGACCAGAAC
CACGTAACAAGATTATAACACATTCCTATCAGCAGCAGTTAAAAATAACAGAAAATGATATAATTTTGTTATC
AAGATGGCTCATCTTGTTACATTTTTTGTAACACATTTATAACAACATTTGTTATATTTTTGACATTGCATTT
ATAAGTTTGATGCAAATAATATCCGATGTCATAAACAGTAACATAGTGTGCAATAGTTTTATTATTTTGTAAC
ATCCTTGCAACACATTCTGTAATAATTTTGTTATTATTATAACAGGGTTTGTTATATTTTAGTTTAGTATGGT
GCAAATTTTGTTAGAATTTTTGTTATTTTAACTACTAACGGTACATGTTTTATAACAACTGGTGATATAAAAA
AATCATATCACGGGAACACATTCTGTTATAATCTTGTTTCTTCCTTCTGGTCGGGATGTCATAAACAGCAACA
TAGTTTGCAATAATTTTGTTATTTTGTAACATCCTTGCAACACATTCTGTAATAATTTTGATATAATGGTAAC
AGGGTTTGTTATATTTTAGTTTAATTTGGTATAAATTTTGTTAGAATTTTTGTTATTTTAACTACTAACGGTA
CATGTTCTATAACAACTGGTGATATAAAAAAATCATATCAGGGGAACACATTATGTTATAATCTTGTTTCTTC
CGTCTGGTCGGGTTGAGCTTTCAAATAGTGGTAATAACAAGTGATTGTGATGTTCCACATGCTAGTTATGATT
TTTCAATCCTTAACGTGAAGATGCATCGAAGCCACACCTCGAATTTTCAAGAGCACAAATCTAGAGAACCAGA
AAACCGTTTGTGCTGAAAATGCTACTCACTGGTCCCTCGCTGGTGGTGACCAATCGATTAGATTTTCAGCACG
AACGGTTGTTAGGTTCTCTAAATTTGTGCTCCTGAAAATTCGAGGTTTGGCTTCGATGCATTTTCACCTTAAA
ATTAGCCCTAAAACACATTTTTAACTTGAAGCATATTTAAATCTTTATCGAATGAGCTGAAAATTTGACCAGC
CATTGTTCTTTGCATGAGAATTCCGAATACTGCTTGACCGGTAGATTTCCAGAAATTTTTTCTAATATGAACA
GGTCTACTGTGGAGTACCAGAGCTCCCTCCCCAGTATTGTGCCCTTCCTGTGCTATCCGGAGCAATGGTGCAG
GTGACCTTGTGTTTCTCCGAGATAATCGGCAGCCCTTCTTCAGTCACAATCCTGAGGCTAAATGTGTGAGGTT
ATGTTTATAAAAATGTTGTAATTTAGTTTAAATTTTCACCTTTTCACATTATGCGTTCTACACAATGTATTCT
GTGCTTTTCGCCTTTGGCGACTTTTAAGAGATAACGATCTGTTTTTTCAGCCTAACAAAACAAACAACTATGC
CGAAGGAATCATACAGCTAACTTGTCATATGACCTAAATGACTATTGAGTCATTTATGCGAGTAGCACTAAGT
AATATTACATTATTTTAATGTATTGTCCTTTTTCAAAGGACCATTTTTGCTAAAGATACCAGTCGTCTATTTC
ATCATTACAACGAGCTAGATGCCGAGCTGCTCACTTGCACTATCGACGCTTAAAGTTTGCCGATTTCTACAGA
CCGACTGTGATACAATTTGGATTATTCTGTATCACATAAATATCATATTGTCTATTCCATCATCACTGATAGC
ACAGACATACAGACGTAACACTTAGAACAATTTTCTTTAAAATCCATCTCCCAGTTTACATCATCATCATCAT
CTGGTGAGCATGATGTGAGAAACGTCAAACAGCGCGCGCATCTGATTATGAGATTTGTAACATAGCAAATTTG
AAATGATTGTCAAAAGAGTGGTCGATGGAAATATCGTCAGTGTTACGTCTGTTTGTCTTTGCTAATAGGTTAA
GCTTAACCTTGAATTATGCAAATAATATTATTTTTTAAAAGTACGATATTGTTGACACGCTGCGTATCGTTTG
AAATTATTTATTAGTCACTGAAAATAGTAGATTACACAACAAGTTGCAGAATGATTATTTTTATTGCACGAGT
CGTACATTTATCGAACGAGGCTTGCCGAGATGGATAACTACGAAGAGAAAAAAAATGCTGTAAAATCGAGTTC
TGCAGCGAGTTGCGTACAACATTTTATGCAATTTTGTAGAAGACCCATTAAGGGAATCAGGAAAACCACCGGG
ATGCAATCACTATTATTTCTCAATTTCTAATGACTGTTACCACACTGCATAATTCAGTGCAGCAGGCCGTTTC
ATGACAGATTGGCGTCAGAGTTGCTCGCTGTCACTATCAACGCCTAAAATTTGCTGATTTGTACAGGCCGACT
GCGATACAATTCGGATCAATCCACATCATATCAATATCATGCTGTCTTCTCCATCACAAGTACATTGATGGCG
ATGGACTATGTATATCACTGATGGGTTAAGTTTAGCCTTAAATTAAGCAGTTAAAATTGCAATTTATCAGTAC
GATATTTTTGACAGCGTTGCAGATACGTTGAAATTATTTGTTGGTCACTGAAAAACAGTAATAGCATGGATAA
TTACCACGTTATCACTCATTCCACAGTGATTATGTTCTAGAGTGCGATGTCGCATCACTGCTGTTGGTTGTCA
AATGAAAGTAATATTGATAGCTCGCAAGTGATATTGATAGTTTTAGACCATCAGCCATCAGCTGATATGATTG
ATATTAAGCAACTCTGATTGGCGTGATGGAAAACAGAAATTGCAAAAACATTAGTCAGCTAAAAATTATGTAC
GCAACTCATTGTAGAATTTGATTTTAATAGCATTCGTCGTAATTATCCAACTTATTTGATAAATTTACGACTC
ATGCTGTGAAAATCATTCTGCAACTTGTTGCGTAAGCTACTATTTTAGGTGCATGGTTCTTATAAGTTTAGAT

AATTACCACGTGATCACTCATGCAGTAGTGATTATGATCTAGAGATCGGTATCACTTCACTGCTGTTAGTTGT
CAAATCAAAAGGATATTAATAGCATAGGCAATTATCAAGACTTAAGACTTAGGAGCAAAGTCTGCTACTTTTG
TGACTCCAGTTTATCATAGCAAATTCCTTAGTACAGTCGACTCTCCACATCTCGATGTTCTACATCTCGATAT
CTCTCCCTATGTCGATGATTTCATAAGTCCCTTCAGTCTGCATACATTTTCACTCTCCATATCTCGATATCCT
CCTTATCTCAATATCTCCATATCTCGATGTGTATCTGTTGATTTTTCGTTCTCAATTTTCTCTCCGTATGTCG
ATGTTATCAATATCGAAGGTTACTAGACCAAAGTTTTGGGATTCAAAACAAATTAGGAGTGCAAAATGACGTT
TGTTTGTGTATTAGTTTCTTGGCAACGAAGTGTTTTTCAATCTAGTATTCATTAAAAATTGGTTCTTTGTCTC
GATCTCTCCCTATCTCGATGGTTCCTTCGATATCGAGATGTGGAGAGGCGACTGTAGGTACATTACCCAGCCT
CTCCTTCTAATTGAAGCGATGCCTTAAAATGTCAGTTTTATTAATATAAGCAGTCAGTGGGTACGAGGTGAGG
TCAGCTGCGGGGAAGGTGGAAGACACAGCTGGAAAGTATTTTTGCATGTATCGTTTCTATGGGAATACTAGGA
ATTGTTTACCACAGAAAACAAATCCAATCGGTGAAGTGAAAATTCTCTTCCGTTTTCCTGAGAGGAATTTGTG
TTTACTTTCGTGTTTCCTTCTGTGTTTGAGATGATGTAATAGGCGGTTTTAAGTTTGAAATCTTGAATACAAG
CCTGTTCAAAAAACTTAATCTAATAATAAGTGATTTAAAACAAACCAAATCTTCAGGCAAAGTGTGTGAGACA
TAGGGGTCTATTTTATAAGTCGAGTCGATCAAAATGACTCGACTGGAGTCACTGTCGACCGAAAAATTCGCTC
GACTCAGCTCTGTCACTCGAGTTTTTCGACAGTTGTCACTCTATGTGACTGGATTACTATGGGAGTCGACGGG
TGACATCTGAAATATGCATGCTTGCTTTGATCGACAATGAGTACAGATGAGTCACATGAATCTGTTCGATTTA
CAAAATAGGCCCCATAACTTATTCTAGCATCCTCCAACCTAAAACAAACTCGTCAGCCGTCATCTGGTATTTC
AACATGGCAGGTGCTGTATTTTGAAGGATTGAACTACCTCCCTTTTAAATACCTTGCAACCGACGAGAAACTT
GAATTTTTTTAACCAGAATAAGCCATTTTACGAAGCTAGTCAAACGACAGGAGACCTGGGATTAAAGTTTAC
CTTTTCGATCATCGTCGTCAGATTTCGCGATGATGATGGTTGATGACTATGCGTATTTCTAGCGTAGCTTTTC
ATACAGGTGAAAACTTAAATGGATAGTAATTATTAAAATATTTCTGATCCCAAAAGTACCTTTTCTTGTGAAA
TATAGGTAACAAAGCGGTAGCTGTCCAGTAAACACAAACTCGTATACGATAGCGCATAAGAGTACAAATGTGG
AGGCGATATACGTACATGCGCCATAAGGCTGCGTGCAAGATGTGCGTATATCGCCTCCACATTTGTACTCTTA
TATCCCATCATATACGATGGTGTGTTTACTGGGTGATCTAGGTGTTATATTGCAGTAACGAACATTTTTGAAA
CACTTATTCGTCATTTTCTCCATGTATCCGTTTAGTAAGATGAGGCGTTGTTAAAAATCACGCTGGATTTTAT
CAAAACAAAAACCCAGGTCTCCTGTCAATTGACGCCGATGCGACGATCAGCTGTTCCGAAACTTCTCGTAAAA
TGGCTTATAGGCCTTTTCATGTGACAGGTCCGTCTATGCATTTGTTTACATCGGTGGAAGACCGGTGCAAACA
CGAGGCTGTCATTTCCATAGAAAAACTGTCAAAGAGCTTCCCGATCAGCTGATTTCGAACGTCTTGTAAATAG
GCCTATACGAACAACGAATCGGAATATTGCTGTCAACATATTTATGGTCACCAACATCAACCGCCGCTTCAAT
TCAAGTGTCAGTTAGAAACGTCAAAAGAAAATGAAACAAGGGGTCTACCAATGTGAATGGGCATGAAGTGGAA
GCAGAAAAAAATGGCAGTCGCATATTGATTGATGTTAAAAGCTCGGAAAAAGTTTTGGTTCACCGAGAACCCA
ACAGTTGAGAATTAGGTTATTTTCATAACCGAATCTCGGCACTTATTTTACCGACATTTAGGCAATTCAATTG
TTTAACCGATGTCTCAGCAAACGTTACCGAGATCACCGTAAAACTTTTATCGAGACTTGGCAAATATTTACCG
AGATATCGGTTGTTGGATTCTCGGTGAATCCGTTTACCGAGGATGATTTCCGAAGTGTGGAGCTGATGTGACT
GATGTTTTGCAACTCTGAATTTAGATTTTTCCGCGTTTATTTTATTCTGCCCCAGTGGTTGGAAAGCACGTAC
ACCCAAATCCATTTACATGAAATGGTTGTTCACAATTACCCAAAAATGTCTGAATGAAGACCTTCGTGTCTAC
ACGCCTAACCAGAGATCTCTCGAAAGTTTTTCAATAAGGTTTATACCCAAGCAGATTCATGCAAGCATATTCT
AACAAATTTCGTCGAGTACATGATTTCAGACCATGAATACAACAACAACAACAAAATTAATAGTGATCTTTGT
AGATAGATCAATAAGCT
>re-annotation of CPIJ000853 using AAEL005733 as homolog FGENESH:[mRNA]
1 15 exon (s) 2001 - 19642 5886 bp, chain +
ATGCCGAAGCCAGTTGTCCAAGTCGGTGACGACCCTGACCCAAGCGAGTGGCTGTTCATT
TCGCTGGAGCAGAAGCGTATCGATCAGAGCAAGCCGTACGATGCCAAGAAGGCGTGCTGG
GTGCCCGACGAAAAGGAGGGCTTTGTCCTCGGTGAAATCAAGGCCACCAAGGGTGAGCTG
GTCACCGTTGGCATTCCCGGAGGCGAGGAGAGGACCATGAAAAAGGATCTAATCTCACAA
GCGAATCCTCCGAAATTCGAAAAAGTCGAAGATATGGCCGATCTGACCTATCTGAACGAA
GCTGCCGTATTGCACAATTTGCGCCAACGATACTACTGCAAAATGATCTACACCTACTCT
GGCTTGTTCTGCGTTGTCATCAATCCTTACAAGCGTTGGCCGCTGTACACCATGCGTGTC
GCCAAGATGTACCGTGGCAAGCGTCGTAATGAGGTCCCGCCCCATCTGTTCGCCGTTTCT
GACGGTGCCTACGTCAACATGTTGACCAACCACGAGAACCAGTCTATGTTGATTACCGGT
GAGTCTGGTGCCGGAAAGACTGAGAACACCAAGAAGGTCATTGCGTACTTCGCCACCATT
GGTGCCTCGAGCAAGAAGAGCGCTGACGAGGAGAAGAAGATCTCCCTGGAAGATCAGGTC
GTCCAGACCAATCCCGTCCTGGAAGCCTACGGTAACGCCAAGACCGTCCGTAACGATAAC
TCGTCTCGTTTCGGTAAATTCATCCGTATCCACTTTACTGGCTCTGGTAAGCTGGGTGGT

GCTGATATTGAAACTTACCTGCTGGAGAAGGCCCGTGTCATCTCTCAGCAGACTCTGGAA
CGCTCCTACCACATCTTCTACCAGATGATGTCCGGCTCGGTCAAGGGACTGAAAGAAATG
TGCTTCCTGTCCAACGATATCTACGATTACTACAACGTCTCTCAGGGTAAAGTTACTATC
CCCAATGTCGATGATGGTGAAGAGTGCCAACTCACCGATGAAGCCTTCAACATCCTGGGC
TTCACTCAGGAGGAGAAGGACAACATCTACAAGATTACCGCCGCTGTCATGCACATGGGT
GGCATGAAGTTCAAGCAAAAGGGTCGCGAAGAGCAGGCTGAAGCCGACGGCACTGAAGAG
GGTGATCGCGTCGCTAAGCTGCTGGGTTGCGTCACTGAGGATCTGTACAAGAACCTGCTG
AAGCCCCGCATCAAGGTCGGTACCGAGTTCGTCACCAAGGGTCAGAACAAGGAACAGGTC
ACCAACGCCGTCGGTGCTCTCTGCAAGGGTATCTTCGATCGTCTGTTCAAGTGGCTGGTC
AAGAAGTGTAACGAGACTCTGGACACCAAGCAGAAGCGCGCTCAGTTCATTGGTGTGCTT
GATATTGCTGGATTCGAGATCTTCGACTACAACGGTTTCGAGCAGCTGTGTATTAACTTC
ACCAACGAAAAGCTGCAGCAGTTCTTCAACCACCACATGTTCGTCCTGGAACAGGAAGAA
TACAAGAAAGAGGGTATTAACTGGGCCTTCATTGATTTCGGTATGGACTTGCTGGCCTGT
ATCGATCTGATTGAAAAGCCCATGGGTATCCTGTCCATTCTTGAGGAAGAGTCTATGTTC
CCCAAGGCTACCGATCAGACCTTTGCTGAGAAGCTGATGAACAACCACTTGGGCAAGTCT
GCTCCGTTCCAGAAGCCCAGGCCACCAAAGCCAGGTTGCCAGGCCGGCCACTTCGCCATC
GGTCACTACGCCGGTACTGTGTCGTACAACATCACCGGATGGCTTGAGAAGAACAAGGAT
CCCCTGAACGACACTGTCGTCGATCAGTTCAAGAAGGGAAAGAACGCGTTGATCGTTGAG
ATCTTCGCTGATCACCCCGGACAGTCCGGTGGTGGCGACGCTGGCGGCAAGGGTGGACGT
GGTAAGAAGGGTGCTGGTTTCGCCACTGTCTCCTCGTCCTACAAGGAGCAGCTGAACAAC
CTGATGACCACTCTGAAGTCTACTCAGCCTCACTTCGTCCGTTGTATCATTCCCAACGAG
TTGAAGCAGACCGGTCTTATCGATGCTCACTTGGTTATGCACCAGCTGACCTGTAACGGT
GTGCTTGAAGGTATCCGTATTTGCCGTAAGGGCTTCCCGAACAGGATGATGTACCCTGAC
TTCAAGCTGCGCTACAAAATCCTAAACCCCAAGGCTGCTGAAGCGGAGAAAGACCCCATG
AAGGTCGCTCAAGTCATCCTGGAAGCTAGTGGTCTTGACACAGAATCATACCGGCTAGGA
AACACCAAGGTCTTCTTCCGTGCCGGTGTCCTGGGTCAGATGGAGGAGTTCCGTGACGAT
CGCCTGTCCAAGATCATGACCTGGATGCAGTCCTGGATCCGTGGCTACCTGTCCCGCAAG
TCTTTCAAGAAGATGCAGGAGCAGCGCGTCTCCCTGGAGATTGTCCAGCGTAACCTGCGC
AAGTACATGAAGCTGCGTACCTGGGCCTGGTGGAAGCTGTGGCAGAAGGTTAAGCCTCTG
CTTAACGTTTCCCGCGTTGAGGACCAGATCGCGAAACTGGAAGAGACCGCCAAGAAGGCT
CAGGATGACTTGGAGAAGGAAACCAAGCTCCGCCAGGAACTGGAGGCTCTGAACAGCAAG
CTGCTGGCTGAGAAGACCGCTCTGTTGGATTCTCTGTCCGGTGAGAAGGGTGCTCTCCAG
GATTTCCAGGAGAAGACCGCCAAGCTCCAGGCCCAGAAGGCCGACGTTGAGAACCAGCTT
CGCGACACCCAGGAGCGCCTGACTCAGGAGGAAGATGCCCGCAACCAGCTCTTCCAGCAG
AAGAAGAAGTTGGAGCAGGAGATCTCTGGCCAGAAGAAGGATGCTGAGGATCTGGAACTG
CAGATCCAGAAGATCGAGCAGGACAAGGCCTCCAAGGATCACCAGATCCGCAACTTGAAC
GATGAGATCGCCCACCAGGACGAGCTGATCAACAAGCTGAACAAGGAGAAGAAGATGTCT
GGTGAGGTCAACCAGAAGACCGCTGAGGAGCTCCAGGCTGCCGAAGATAAGGTCAACCAC
CTGAACAAGGTTAAGGCCAAGCTGGAGCAGACTCTGGATGAGCTGGAGGACTCTCTGGAG
CGCGAGAAGAAGCTGCGCGGTGATGTTGAGAAGGCTAAGCGCAAGGTTGAGGGTGACCTG
AAGCTGACTCAGGAAGCCGTCGCTGATCTGGAGCGCAACAAGAAGGAGCTTGAGCAGACC
ATCATGCGCAAGGACAAGGAAATCTCTGCCTTGTCTGCTAAGCTGGAGGACGAACAGTCC
CTGGTTGGCAAGCTGCAGAAGCAGATCAAGGAACTGCAGGGCCGCATTGAGGAGCTCGAG
GAGGAAGTCGAGGCTGAGCGCCAGGCTCGTGCCAAGGCTGAGAAGCAGCGCGCCGATCTG
GCCCGCGAACTCGAGGAACTGGGTGAGCGTCTGGAGGAAGCCGGTGGTGCCACCTCGGCC
CAGATTGAGCTGAACAAGAAGCGTGAGGCTGAGCTCGCCAAGCTGCGTCGCGACTTGGAG
GAGTCCAACATCCAGCATGAGGGAACTCTGGCTAACCTGCGCAAGAAGCACAACGATGCC
GTCGCTGAGATGGCTGAGCAGGTCGACCAGCTGAACAAGCTGAAGACCAAAGCTGAAAAA
GAGAGAGGCCAATACTTCGCTGAACTGAACGACTCCCGTCTCAGTTTAGATCATCTGGCT
AATGAGAAGGCTTCCCAGGAGAAGATCGCCAAGCAGCTGCAGCACACTCTGAACGAAGTT
CAGGGCAAGCTGGACGAAACCAACCGCACTCTGAACGACTTCGACACGTCCAAGAAGAAG
CTGTCCATTGAGAACTCTGACCTGCTCCGCCAGTTGGAGGATGCCGAGTCTCAGGTTTCG
CAGCTGAGCAAGATCAAGATCTCGCTCACTCAGCAGCTCGAGGATACCAAGCGTCTGGCC
GATGAGGAGTCTCGCGAACGCGCTACTCTGCTCGGCAAGTTCCGCAACCTGGAGCACGAC

CTCGACAGCCTGCGTGAACAGGTTGAGGAGGAGGCTGAGGGCAAGGGAGACATCCAGCGC
CAGCTCAGCAAGGCCAACGCCGAAGCCCAGCTGTGGCGTACCAAGTACGAGTCGGAGGGT
GTTGCCCGCGCTGAGGAGCTCGAGGAAGCCAAGAGGAAGCTGCAGGCCCGCCTTGCCGAG
GCTGAGGAGACCATTGAGTCGCTCAACCAGAAGTGCATTGCTCTGGAGAAGACCAAGCAG
CGTCTGTCCACCGAAGTCGAGGATCTGCAGCTCGAGGTCGACCGTGCCACCTCGATCGCC
AACTCTGCCGAGAAGAAGCAGAAGGCCTTCGACAAGATCATCGGCGAATGGAAGCTCAAG
GTCGACGATCTGGCTGCCGAGCTGGACGCTTCCCAGAAAGAATGCCGCAACTACTCGACC
GAGCTGTTCCGTCTCAAGGGTGCCTACGAAGAGGGCCAGGAGCAGCTTGAGGCTGTCCGC
CGTGAGAACAAGAACTTGGCTGATGAGGTCAAGGATCTGCTGGACCAGATCGGTGAGGGT
GGCCGCAACATCCACGAGATTGAGAAGTCTCGCAAGCGCCTGGAGGCTGAGAAGGACGAG
CTGCAGGCCGCCCTTGAGGAAGCCGAGGCTGCTCTGGAACAGGAGGAGAACAAGGTTCTG
CGCGCTCAGCTTGAGCTGTCTCAGGTGCGCCAGGAAATTGACCGCCGCATCCAGGAGAAG
GAAGAGGAATTCGAAAACACCCGCAAGAACCACCAGCGTGCCCTGGACTCCATGCAGGCC
TCTCTTGAAGCCGAAGCCAAGGGTAAGGCTGAGGCCCTGCGCATGAAGAAGAAGCTGGAG
GCTGACATCAACGAGCTTGAGATTGCTCTGGATCATGCCAACAAGGCTAACGCTGAGGCC
CAGAAGAACATCAAGCGCTACCAGCAGCAGATGAAGGATGTCCAGAGCGCCCTGGAGGAA
GAACAGCGTGCCCGTGACGATGCCCGCGAACAGCTGGGTATCTCTGAGCGTCGCGCCAAC
GCCCTGCAGAACGAACTGGAGGAGTCGCGTACTCTGCTGGAGCAGGCCGACCGTGGCCGT
CGCCAGGCTGAACAGGAGCTGGGTGATGCCCACGAGCAGCTGAACGACGTTTCTGCCCAG
AACGCTTCGATCGCCGCCGCCAAGAGGAAGCTGGAGTCTGAGCTGCAGACCCTGCACTCC
GACCTGGATGAGCTGCTGAACGAAGCCAAGAACTCCGAGGAGAAGGCCAAGAAGGCTATG
GTTGATGCCGCCCGCCTGGCCGATGAGCTCCGCGCTGAGCAGGACCACGCCCAGTCCCAG
GAGAAGATGCGCAAGGCCCTTGAGCAGCAGATCAAGGAACTGCAGGTCCGTCTGGATGAC
GCCGAGACCAACGCTCTGAAGGGAGGCAAGAAGGCCATTCAGAAGCTGGAGCAGCGCGTC
CGCGAGCTGGAAGCCGAGCTGGACAGCGAGCAGAGAAGACACACCGATGCCCAGAAGAAC
CTCCGCAAGTCCGAGCGTCGCATCAAGGAGTTGACCTTCCAGTCTGAGGAAGACCGCAAG
AACCACGAACGCATGCAGGACCTCGTCGACAAGCTGCAGCAGAAGATCAAGACTTACAAG
AGGCAGATTGAGGAAGCCGAGGAGATCGCCGCTCTGAATCTGGCCAAGTTCCGCAAGGCC
CAGCAGGAGCTGGAGGAGGCTGAGGAGCGTGCCGACATTGCCGAGCAAACTGCCACCAAA
TTCCGCACCAAGGGAGGACGTGCCGGTTCCGTGCAGCGCGGTGCCAGCCCAGCACCCCAG
AGACAGTCGGCCATGCCATCTCTCGCCGCTCTTGGACTTCCCACATTCGACGACCATGCT
TTCTAA
