# Supplementary material for: Insights into the Preservation of the Homomorphic Sex-Determining Chromosome of Aedes aegypti from the Discovery of a Male-Biased Gene Tightly Linked to the M-Locus
Source: Genome Biol Evol. 2014 Jan 6;6(1):179–91. doi: 10.1093/gbe/evu002 (PMC3914700; doi:10.1093/gbe/evu002)
Supplement: Supplementary Data [file supp_evu002_supplemental-file-S2-phylogeny-parameters_2.pdf]

```
#NEXUS
[TITLE: myo-sex]

begin data;
dimensions ntax=13 nchar=2698;
format interleave datatype=protein   gap=- ;

matrix
RPRC012274-PA              -PAGMQSGGD DPDPTPYLFV SLEQKRLDQT KPYDAKKACW VPDEAEGFLQ
CPIJ000848-RA              MPKPVVQVGD DPDPGPWLFI SEEMKKEAMA KPYDGKKACW VPDEKEGFLQ
AGAP010147-PA              MPKPPVQVGE DPDPTEFLFV SLEQKRIDQS KPYDSKKACW VPEEKEGYVL
CPIJ000853-RA              ---------- ---------- ---------- ---------- ----------
AAEL005733-RB              MPKPVVQVGD DPDPSEWLFV SLEQKRIDQS KPYDAKKACW VPDEKEGYVL
CPIJ000849-RA              MPKPVVQVGD DPDPGPWLFI SEEMKKEAMS RPYDGKKACW VPDEKEGFLQ
AAEL005656-RA              MPKPVVQVGG DPDPSEWLFI SEEMKRIDQS KPYDAKKACW VPDEAEGYLL
MyoSex                     MPKPVVQVGD DPDPSEWLYI SEEMRRIDQS KPYDAKKACW VPDEAEGYVQ
XP_001814139.1_Tc          MPKPEKTE-E DPDPTPYLFV SLEQKRIDQT KPYDAKKSCW VPDEKEGFVL
GMOY005703-PA              MPRPIASQ-E DEDPTPYLFV SLEQRRIDQS KPYDAKKNCW VPDEKEGFLL
FBpp0080463                MPKPVANQ-E DEDPTPYLFV SLEQRRIDQS KPYDSKKSCW IPDEKEGYLL
BAG30740.1                 MPKPQVQEGE DPDPTPYLFV SLEQKRIDQS KPYDGKKACW VPDEKEGFVQ
LLOTMP009501-PA            MPKVVKQEGE DPDPTPYLFV SLEQKRIDQT KPYDSKKNCW VPDEKDGYLL


RPRC012274-PA              GEIKGTKGDI VTVSLPNGE- ---------- ---------- ----------
CPIJ000848-RA              GEIKATKGDL VTVALPGGE- ---------- ---------- ----------
AGAP010147-PA              GEIKATKGEL VTVALPGGE- ---------- ---------- ----------
CPIJ000853-RA              ---------- ---------- ---------- ---------- ----------
AAEL005733-RB              GEIKATKGEL VTVGLPGGE- ---------- ---------- ----------
CPIJ000849-RA              GEIKATKGDL VTVALPGGE- ---------- ---------- ----------
AAEL005656-RA              GEIKATKGEL VTVALPGGE- ---------- ---------- ----------
MyoSex                     GEIKATKGDL VTVALPGGE- ---------- ---------- ----------
XP_001814139.1_Tc          GEIKGTKGDL VTVGLPGGE- ---------- ---------- ----------
GMOY005703-PA              GEIKATKGDL VTVNLPGGEL KDFKADKVEK TNPPKFEKIE DMADMTVLNT
FBpp0080463                GEIKATKGDI VSVGLQGGE- ---------- ---------- ----------
BAG30740.1                 GEIKATKGDL VTVNLPGGE- ---------- ---------- ----------
LLOTMP009501-PA            GEIKATKGDL VTVSLPGGE- ----KEQLSQ VNPPKFEKVE DMADLTYLNE


RPRC012274-PA              ---------- -------TKD FKKDLVGQVN PPKFEKCEDM SNLTYLNDAS
CPIJ000848-RA              ---------- -------CKD FKKDLVGQVN PPKYEKCEDL SNLTYLNDAS
AGAP010147-PA              ---------- -------EKN FKKEQLSQVN PPKFEKVEDM ADLTYLNEAA
CPIJ000853-RA              ---------- ---------- ---------- ---------- ----------
AAEL005733-RB              ---------- -------EKN FKKELISQVN PPKFEKVEDM ADLTYLNEAA
CPIJ000849-RA              ---------- -------CKD FKKDLVGQVN PPKYEKCEDL SNLTYLNDAS
AAEL005656-RA              ---------- -------TKD FKKDLVGQVN PPKYEKCEDM SNLTYLNDAS
MyoSex                     ---------- -------TKD FKKDLVGQVN PPKYEKCEDM SNLTYLNDAS
XP_001814139.1_Tc          ---------- -------EKN FKKEQVGQVN PPKYEKCEDM SNLTYLNDAS
GMOY005703-PA              PCVLHNLRQR YYSKIIYSKD FKKDLLTQVN PPKYEKAEDM SNLTYLNDAS
FBpp0080463                ---------- -------TRD LKKDLLQQVN PPKYEKAEDM SNLTYLNDAS
BAG30740.1                 ---------- -------TKD FKKDLVAQVN PPKYEKCEDM SNLTYLNDAS
LLOTMP009501-PA            ASVLHNLKQR YYYKLIYTRD FKKDQLQQVN PPKYEKAEDM SNLTYLNDAS


RPRC012274-PA              VLYNLKQRYY FKLIYTYSGL FCVAINPYKR FPVYTMRCAK LYRGKRRNEV
CPIJ000848-RA              VLHNLRERYR AQLIYTYSGL FCIVINPYKR WPLYTMRAAK MYRGKRRNEV
AGAP010147-PA              VLHNLRQRYY SKLIYTYSGL FCVVINPYKR YPLYTNRCAK MYRGKRRNEV
CPIJ000853-RA              ---------- ---------- ---------- ---------- ----------
AAEL005733-RB              VLHNLRQRYY SKLIYTYSGL FCVVINPYKR WPLYTLRVAK MYRGKRRNEV
CPIJ000849-RA              VLHNLRERYR AQLIYTYSGL FCIVINPYKR WPLYTMRAAK MYRGKRRNEV
AAEL005656-RA              VLHNLRERYR ARLIYTYSGL FCIVINPYKR WPLYTLRVAK MYRGKRRNEV
MyoSex                     VLHNLRERYR ARLIYTYSGL FCIVINPYKR WPLYTLRVAK MYRGKRRNEI
XP_001814139.1_Tc          VLHNLKQRYY AKLIYTYSGL FCVAINPYKR FPVYTNRCAK LYRGKRRNEV
GMOY005703-PA              VLHNLRQRYY NKLIYTYSGL FCIAINPYKR YPVYTNRCAK MYRGKRRNEV
FBpp0080463                VLHNLRQRYY NKLIYTYSGL FCVAINPYKR YPVYTNRCAK MYRGKRRNEV
BAG30740.1                 VLYNLKQRYY HKLIYTYSGL FCVAINPYKR FPVYTTRCAR LYRGKRRSEV
LLOTMP009501-PA            VLHNLKQRYY NQLIYTYSGL FCVAINPYKR FPVYTNRCAK MYRGKRRNEV
```

```
RPRC012274-PA           PPHIFAISDG AYVNMLTNKE NQSMLITGES GAGKTENTKK VIAYFATVGA
CPIJ000848-RA           PPHLFAVSDG AYVNMLSNKE NQSMLITGES GAGKTENTKK VIAYFATIGA
AGAP010147-PA           PPHLFAVSDG AYVNMLTNHE NQSMLITGES GAGKTENTKK VIAYFATIGA
CPIJ000853-RA           ---------- ---------- ---------- ---------- ----------
AAEL005733-RB           PPHLFAVSDG AYVNMLTNHE NQSMLITGES GAGKTENTKK VIAYFATIGA
CPIJ000849-RA           PPHLFAVSDG AYVNMLSNKE NQSMLITGES GAGKTENTKK VIAYFATIGA
AAEL005656-RA           PPHLFAISDG AYVNMLTNKE NQSMLITGES GAGKTENTKK VIAYFATIGA
MyoSex                  PPHLFAVSDG AYVNMLTNKE NQSMLITGES GAGKTENTKK VIAYFATIGA
XP_001814139.1_Tc       PPHIFAISDG AYVNMLTNHE NQSMLITGES GAGKTENTKK VIAYFATVGA
GMOY005703-PA           PPHIFAISDG AYVDMLTNHV NQSMLITGES GAGKTENTKK VIAYFATVGA
FBpp0080463             PPHIFAISDG AYVDMLTNHV NQSMLITGES GAGKTENTKK VIAYFATVGA
BAG30740.1              PPHIFAISDG AYVNMLTNHE NQSMLITGES GAGKTENTKK VIAYFATVGA
LLOTMP009501-PA         PPHIFAISDG AYVDMLTNHQ NQSMLITGES GAGKTENTKK VIAYFATVGA


RPRC012274-PA           STKKEDAGVE KKGTLEDQVV QTNPVLEAFG NAKTVRNDNS SRFGKFIRIH
CPIJ000848-RA           SSKKSAEE-E KKISLEDQVV QTNPVLEAYG NAKTVRNDNS SRFGKFIRIH
AGAP010147-PA           SGKKDENA-E KKGSLEDQVV QTNPVLEAFG NAKTVRNDNS SRFGKFIRIH
CPIJ000853-RA           ---------- ---------- ---------- ---------- ----------
AAEL005733-RB           STKKEESS-E KKASLEDQVV QTNPVLEAYG NAKTVRNDNS SRFGKFIRIH
CPIJ000849-RA           SSKKSAEE-E KKISLEDQVV QTNPVLEAYG NAKTVRNDNS SRFGKFIRIH
AAEL005656-RA           SKK---DT-E GKPSLEDQVV QTNPVLEAYG NAKTVRNDNS SRFGKFIRIH
MyoSex                  SKK---DT-E GKPSLEDQVV QTNPVLEAYG NAKTVRNDNS SRFGKFIRIH
XP_001814139.1_Tc       STKKSEEQ-A KKGNLEDQVV QTNPVLEAFG NAKTVRNDNS SRFGKFIRIH
GMOY005703-PA           S-KKDESQ-K NKGSLEDQVV QTNPVLEAFG NAKTVRNDNS SRFGKFIRIH
FBpp0080463             SKKTDEAA-K SKGSLEDQVV QTNPVLEAFG NAKTVRNDNS SRFGKFIRIH
BAG30740.1              SQKKDPSQ-E KKGSLEDQVV QTNPVLEAFG NAKTVRNDNS SRFGKFIRIH
LLOTMP009501-PA         SGKKDESS-S KKGTLEDQVV QTNPVLEAFG NAKTVRNDNS SRFGKFIRIH


RPRC012274-PA           FGPSGKLAGA DIETYLLEKA RVISQQTLER SYHIFYQIMS GAVKTLKPMC
CPIJ000848-RA           FTASGKLGGA DIETYLLEKA RVISQQTLER SYHIFYQMMA GSVKGLKEMC
AGAP010147-PA           FTGSGKLAGA DIETYLLEKA RVISQQTLER SYHIFYQIMS GSVKGLKEKC
CPIJ000853-RA           ---------- ---------- ---------- ---------- --------MC
AAEL005733-RB           FTGSGKLAGA DIETYLLEKA RVISQQSLER SYHIFYQMMS GSVKGLKDMC
CPIJ000849-RA           FTGSGKLGGA DIETYLLEKA RVISQQTLER SYHIFYQMMS GSVKGLKDMC
AAEL005656-RA           FTASGKLAGA DIETYLLEKA RVISQQTLER SYHIFYQMMS GSVKGLKDMC
MyoSex                  FTASGKLAGA DIETYLLEKA RVISQQTLER SYHIFYQMMS GSVKGLKEMC
XP_001814139.1_Tc       FGPTGKLAGA DIETYLLEKA RVISQQSLER SYHIFYQMMS GAVKGLKENC
GMOY005703-PA           FGPSGKLAGA DIETYLLEKA RVISQQSLER SYHIFYQIMS NSVAGVKEMC
FBpp0080463             FGPTGKLAGA DIETYLLEKA RVISQQSLER SYHIFYQIMS GSVPGVKEMC
BAG30740.1              FGPSGKLAGA DIETYLLEKA RVISQQALER SYHIFYQMMS GSVAGLKDMC
LLOTMP009501-PA         FGPSGKLAGA DIETYLLEKA RVISQQSLER SYHIFYQMMS GSVDGLKAKC


RPRC012274-PA           CLSENIHDYV FVSQGKTTIN GVDDGEEMML TDVSDDIYEY NFVSQGKISI
CPIJ000848-RA           YLSNDIYDYY NVSQGKVTIP NVDDGEECA- ---------- ----------
AGAP010147-PA           LLSNNIHDYH IVAQGKTTIP SVDDGEEMQ- ---------- ----------
CPIJ000853-RA           FLSNDIYDYY NVSQGKVTIP NVDDGEECQ- ---------- ----------
AAEL005733-RB           FLSNDIYDYY NVAQGKITIP NVDDGEECL- ---------- ----------
CPIJ000849-RA           YLSNDIYDYY NVSQGKVTIP NVDDGEECL- ---------- ----------
AAEL005656-RA           LLSNDIYDYH NVAQGKVTIP NVDDGEECR- ---------- ----------
MyoSex                  FLSNDIYDYV NVSQGKITIP NVDDGEECM- ---------- ----------
XP_001814139.1_Tc       LLSDNVYDYN FVSQGKVTIP GVDDAEELE- ---------- ----------
GMOY005703-PA           LLTDNIYDYH NVSQGKVTVA SIDDSEEFQ- ---------- ----------
FBpp0080463             FLSDNIYDYY NVSQGKVTVP NMDDGEEFQ- ---------- ----------
BAG30740.1              LLSNDVYDYH IVSQGKTTIP NVDDGEECL- ---------- ----------
LLOTMP009501-PA         LLSNDIYDYH NVAQGKITIP NVDDGEECVA TD-------- -----GKTTI


RPRC012274-PA           AGVDDAEEMS LTDQAFDVLG FTQEEKDNIY KITAAVMHMG TMKFKQRGRE
CPIJ000848-RA           ---------- LTDEAFNILG FTQEEKDNIY KITAAVMHMG GMKFKQKGRE
AGAP010147-PA           ---------- ITDEAFNVLG FTQEEKDNIY RITSAVMHMG RMQFKQKGRE
CPIJ000853-RA           ---------- LTDEAFNILG FTQEEKDNIY KITAAVMHMG GMKFKQKGRE
```

```
AAEL005733-RB         ---------- LTDEAFNVLG FTQEEKDNIY KITAAVMHMG GMKFKQKGRE
CPIJ000849-RA         ---------- LTDEAFNILG FTQEEKDNIY KITAAVMHMG GMKFKQKGRE
AAEL005656-RA         ---------- LTDEAFDILG FTQEEKDNIY KITAAVMHMG GMKFKQKGRE
MyoSex                ---------- LTDVAFDVLG FTQDEKDNIY KITAAVMHMG GMKFKQKGRE
XP_001814139.1_Tc     ---------- LTDQAFDVLG FTQEEKDNIY KITAAVMHMG CMKFKQRGRE
GMOY005703-PA         ---------- LTDQAFDILG FTRDEKENVY RITAAVMHMG GMKFKQRGRE
FBpp0080463           ---------- LADQAFDILG FTKQEKEDVY RITAAVMHMG GMKFKQRGRE
BAG30740.1            ---------- LTDQAFDILG FTQEEKDNVY KITAAVMHMG CMKFKQRGRE
LLOTMP009501-PA       PSVDDAEEMQ ITDQAFDVLG FTQEEKDNVY KITAAVMHMG GMKFKQRGRE

RPRC012274-PA         EQAEADGTEE GDKVGKLLGV EGQDLYKNLV KPRIKVGNEF VTQGRNVSQV
CPIJ000848-RA         EQAEADGTDE GDRVAKLLGC VTEDLYKNLL KPRIKVGAEF VVKGQNKDQV
AGAP010147-PA         EQAEADGTED GDRVAKLLGV GTDDLYKNLL KPRIKVGNEF VTKGQNKDQV
CPIJ000853-RA         EQAEADGTEE GDRVAKLLGC VTEDLYKNLL KPRIKVGTEF VTKGQNKEQV
AAEL005733-RB         EQAEADGMEE GDRVAKLLGC VTEDLYKNLL KPRIKVGAEF VTKGQNKDQV
CPIJ000849-RA         EQAEADGTDE GDRVAKLLGC VTEDLYKNLL KPRIKVGAEF VIKGQNKEQV
AAEL005656-RA         EQAEADGIEE GDRVAKLLGC VTDDLYKNLL KPRIKVGTEY VTKGQNKDQV
MyoSex                EQAEADGTDE GDRVAKLLGC VTDDLYKNLL KPRIKVGTEY VTKGQNKDQV
XP_001814139.1_Tc     EQAEPDGTEE GERVAKLLGI EAPGLYNALC KPRIKVGAEF VTQGRNVNQV
GMOY005703-PA         EQAEQDGEEE GGRVAKLFGC DTAELYKNLL KPRIKVGNEF VTQGRNVQQV
FBpp0080463           EQAEQDGEEE GGRVSKLFGC DTAELYKNLL KPRIKVGNEF VTQGRNVQQV
BAG30740.1            EQAEADGTED GDKVAKLLGV DCQDLYKNLL KPRIKVGNEF VTQGRNKDQV
LLOTMP009501-PA       EQAEADGTEE GERVATLLGT DTQELYKNLL KPRIKVGNEF VTQGRNKDQV

RPRC012274-PA         SYSVGAMSKG MFDRLFKFLV KKCNETLDTK QKRQHFIGVL DIAGFEIFDY
CPIJ000848-RA         TNSVGALCKG IFDRLFKWLV KKCNETLDTK QKRAQFIGVL DIAGFEIFDY
AGAP010147-PA         TNSVGALCKG IFDRLFKWLV KKCNETLDTK QKRAQFIGVL DIAGFEIFDN
CPIJ000853-RA         TNAVGALCKG IFDRLFKWLV KKCNETLDTK QKRAQFIGVL DIAGFEIFDY
AAEL005733-RB         TNAVGALCKG IFDRLFKWLV KKCNETLDTQ MKRVQFIGVL DIAGFEIFDY
CPIJ000849-RA         SNAVGALCKG IFDRLFKWLV KKCNETLDTK QKRAQFIGVL DIAGFEIFDY
AAEL005656-RA         YNAVGALCKG IFDRLFKWLV KKCNETLDTK QKRAQFIGVL DIAGFEIFDF
MyoSex                SNAVGALCKG IFDRLFKWLV KKCNETLDTK QKRAQFIGVL DIAGFEIFDF
XP_001814139.1_Tc     NYSVGAMSKA MFDRLFKFLV KKCNETLDTK QKRQHFIGVL DIAGFEIFDY
GMOY005703-PA         TNSIGALCKG VFDRLFKWLV KKCNETLDTK QKRQHFIGVL DIAGFEIFDY
FBpp0080463           TNSIGALCKG VFDRLFKWLV KKCNETLDTQ QKRQHFIGVL DIAGFEIFEY
BAG30740.1            TNSVGALCKG MFDRLFKWLV KKCNETLDTK QKRQHFIGVL DIAGFEIFDF
LLOTMP009501-PA       SNSVGALCKG IFDRLFKWLV KKCNETLDTQ QKRQHFIGVL DIAGFEIFDY

RPRC012274-PA         NGFEQLCINF TNEKLQQFFN HHMFVLEQEE YKREGINWAF IDFGMDLLAC
CPIJ000848-RA         NGFEQLCINF TNEKLQQFFN HHMFVLEQEE YKKEGIVWAF IDFGMDLLAC
AGAP010147-PA         NGFNQLCINF TNERLQQFFN HYMFILEQEE YEREGIQWTF IDFGLDLQPT
CPIJ000853-RA         NGFEQLCINF TNEKLQQFFN HHMFVLEQEE YKKEGINWAF IDFGMDLLAC
AAEL005733-RB         NGFEQLCINF TNEKLQQFFN HHMFVLEQEE YKKEGINWAF IDFGMDLLAC
CPIJ000849-RA         NGFEQLCINF TNEKLQQFFN HHMFVLEQEE YKKEGIVWAF IDFGMDLLAC
AAEL005656-RA         NGFEQLCINF TNEKLQQFFN HHMFVLEQEE YKKEGINWAF IDFGMDLLAC
MyoSex                NGFEQLCINF TNEKLQQFFN HHMFVLEQEE YKKEGINWAF IDFGMDLLAC
XP_001814139.1_Tc     NGFEQLCINF TNEKLQQFFN HHMFVLEQEE YKAEGIQWTF IDFGMDLLAC
GMOY005703-PA         NGFEQLCINF TNEKLQQFFN HHMFVLEQEE YKKEGINWDF IDFGMDLLAC
FBpp0080463           NGFEQLCINF TNEKLQQFFN HIMFVMEQEE YKKEGINWDF IDFGMDLLAC
BAG30740.1            NGFEQLCINF TNEKLQQFFN HHMFVLEQEE YTREGIHWAF IDFGMDLLAC
LLOTMP009501-PA       NGFEQLCINF TNEKLQQFFN HHMFVLEQET YKQEGIHWEF IDFGMDLLAC

RPRC012274-PA         IELIEKYNGF EQLCINFTNE KLQQFFNHHM FVLEQEEYKK EGIDWAFIDF
CPIJ000848-RA         ---------- ---------- ---------- ---------- ----------
AGAP010147-PA         ---------- ---------- ---------- ---------- ----------
CPIJ000853-RA         IELIEKYNGF EQLCINFTNE KLQQFFNHHM FVLEQEEYKK EGINWAFIDF
AAEL005733-RB         ---------- ---------- ---------- ---------- ----------
CPIJ000849-RA         ---------- ---------- ---------- ---------- ----------
AAEL005656-RA         ---------- ---------- ---------- ---------- ----------
MyoSex                ---------- ---------- ---------- ---------- ----------
XP_001814139.1_Tc     ---------- ---------- ---------- ---------- ----------
GMOY005703-PA         IDLIEKYNGF EQLCINFTNE KLQQFFNHHM FVLEQEEYKK EGIEWDFIDF
```

```
FBpp0080463             ---------- ---------- ---------- ---------- ----------
BAG30740.1              ---------- ---------- ---------- ---------- ----------
LLOTMP009501-PA         IDLIEKYNGF EQLCINFTNE KLQQFFNHHM FVLEQEEYKI EGIQWTFIDF


RPRC012274-PA           GMDLLACIEL IEKFNGFEQL CINFTNEKLQ QFFNHHMFVL EQEEYTREGI
CPIJ000848-RA           ---------- ---------- ---------- ---------- ----------
AGAP010147-PA           ---------- ---------- ---------- ---------- ----------
CPIJ000853-RA           GMDLLACIDL IEKYNGFEQL CINFTNEKLQ QFFNHHMFVL EQEEYQREGI
AAEL005733-RB           ---------- ---------- ---------- ---------- ----------
CPIJ000849-RA           ---------- ---------- ---------- ---------- ----------
AAEL005656-RA           ---------- ---------- ---------- ---------- ----------
MyoSex                  ---------- ---------- ---------- ---------- ----------
XP_001814139.1_Tc       ---------- ---------- ---------- ---------- ----------
GMOY005703-PA           GMDLLACIDL IEKFNGFEQL CINFTNEKLQ QFFNHHMFVL EQEEYQREGI
FBpp0080463             ---------- ---------- ---------- ---------- ----------
BAG30740.1              ---------- ---------- ---------- ---------- ----------
LLOTMP009501-PA         GMDLLACIEL IEKYNGFEQL CINFTNEKLQ QFFNHHMFVL EQEEYQREGI


RPRC012274-PA           EWAFIDFGMD LAACIELIEK ---------- -----LQQFF NHHMFVLEQE
CPIJ000848-RA           ---------- ---------- ---------- ---------- ----------
AGAP010147-PA           ---------- ---------- ---------- ---------- ----------
CPIJ000853-RA           EWTFIDFGMD LQQCIELIEK YNGFNQLCIN FTNERLQQFF NHYMFILEQE
AAEL005733-RB           ---------- ---------- ---------- ---------- ----------
CPIJ000849-RA           ---------- ---------- ---------- ---------- ----------
AAEL005656-RA           ---------- ---------- ---------- ---------- ----------
MyoSex                  ---------- ---------- ---------- ---------- ----------
XP_001814139.1_Tc       ---------- ---------- ---------- ---------- ----------
GMOY005703-PA           EWTFIDFGMD LQLC------ ---------- ---------- ----------
FBpp0080463             ---------- ---------- ---------- ---------- ----------
BAG30740.1              ---------- ---------- ---------- ---------- ----------
LLOTMP009501-PA         EWTFIDFGMD LQSC------ ---------- ---------- ----------


RPRC012274-PA           EYAREGIEWA FIDFGMDLAA CIELIEKPMG ILSILEEESM FPKATDKTFE
CPIJ000848-RA           ---------- ---------- -IELIERPMG ILSILEEESM FPKATDQTFA
AGAP010147-PA           ---------- ---------- -IDLIEKPMG ILSILEEESM FPKATDQTFA
CPIJ000853-RA           EYEREGIQWT FIDFGLDLQP TIDLIEKPMG ILSILEEESM FPKATDQTFA
AAEL005733-RB           ---------- ---------- -IDLIEKPMG ILSILEEESM FPKATDQTFA
CPIJ000849-RA           ---------- ---------- -IELIERPMG ILSILEEESM FPKATDQTFA
AAEL005656-RA           ---------- ---------- -IELIEKPMG ILSILEEESM FPKATDQTFA
MyoSex                  ---------- ---------- -VELIEKPMG ILSILEEESM FPKATDQTFV
XP_001814139.1_Tc       ---------- ---------- -IELIEKPMG ILSILEEESM FPKATDKTFE
GMOY005703-PA           ---------- ---------- -IDLIEKPMG ILSILEEESM FPKATDQTFA
FBpp0080463             ---------- ---------- -IDLIEKPMG ILSILEEESM FPKATDQTFS
BAG30740.1              ---------- ---------- -IDLIEKPMG ILSILEEESM FPKATDQTFV
LLOTMP009501-PA         ---------- ---------- -IELIEKPMG ILSILEEESM FPKATDQTFA


RPRC012274-PA           DKLNTNHLGK SPNFQKPKPP KPGCQAAHFA IGHYAGVVSY NITGWLEKNK
CPIJ000848-RA           EKLMTNHLGK SPPFQKPRPS KPGIPAGHFA IGHYAGVVTY NITGWLEKNK
AGAP010147-PA           EKLMTNHLGK SAPFMKPRPP KPGIPAGHFA IGHYAGVVSY NITGWLEKNK
CPIJ000853-RA           EKLMNNHLGK SAPFQKPRPP KPGCQAGHFA IGHYAGTVSY NITGWLEKNK
AAEL005733-RB           EKLMNNHLGK SAPFQKPKPP KPGCQAAHFA IGHYAGVVSY NITGWLEKNK
CPIJ000849-RA           EKLMTNHLGK SPPFQKPRPS KPGIPAGHFA IGHYAGVVTY NITGWLEKNK
AAEL005656-RA           EKLMNNHLGK SPPFMKPRPP KPGIPAGHFA IGHYAGVVSY NITGWLEKNK
MyoSex                  EKLITNHLGK SAPFMKPRPP KPGIPAGHFA IGHYAGVVSY NITGWLEKNK
XP_001814139.1_Tc       EKLNTNHLGK SPNFLKPKPP KPGQQAAHFA IGHYAGNVPY NITGWLEKNK
GMOY005703-PA           EKLVNTHLGK SAPFQKPKPP KPGQQAAHFA IGHYAGVVAY NITGWLEKNK
FBpp0080463             EKLTNTHLGK SAPFQKPKPP KPGQQAAHFA IAHYAGCVSY NITGWLEKNK
BAG30740.1              EKLNNNHLGK SAPYLKPKPP KPGCQAAHFA IGHYAGNVGY NITGWLEKNK
LLOTMP009501-PA         EKLNTNHLGK SAPFQKPKPP KPGCQAAHFA IGHYAGVVPY NITGWLEKNK


RPRC012274-PA           DPLNDTVVDQ FKKGSNKLLI EIFADHPGQS GAP----DA- GGGKGGRGKK
```

```
CPIJ000848-RA          DPLNDTVIDQ FKKGTNALIV EIFADHPGQS GPPPGSDDGK GGKGGGRGKK
AGAP010147-PA          DPLNDTVVDQ FKKGSNALMV EIFADHPGQS ADP------- AAAKGGRGKK
CPIJ000853-RA          DPLNDTVVDQ FKKGKNALIV EIFADHPGQS GGG----DA- -GGKGGRGKK
AAEL005733-RB          DPLNDTVVDQ FKKGQNKLVV EIFADHPGQS GGA----DA- GGGKGGRGKK
CPIJ000849-RA          DPLNDTVVDQ FKKGTNALIV EIFADHPGQS GPPPGSDDGK GGKGGGRGKK
AAEL005656-RA          DPLNDTVVEQ FKKGENALLV EIFSEAAPVV AEG-----AP PAKGGGRGKK
MyoSex                 DPLNDTVVEQ FKKGENSLLV EIFSEAAAAP AEG-----AA PAAKGGRGKK
XP_001814139.1_Tc      DPLNDTVVDL YKKGTNKLLV EIFADHPGQS GAP----DA- -GGGKGRGKK
GMOY005703-PA          DPLNDTVVDQ FKKSKNALLV EIFADHPGQS GGG----EQ- --AKGGRGKK
FBpp0080463            DPLNDTVVDQ FKKSQNKLLI EIFADHAGQS GGG----EQ- --AKGGRGKK
BAG30740.1             DPLNDTVVDQ FKKGANKLLV EIFADHPGQS GDA----SAR GGGKGGRGEK
LLOTMP009501-PA        DPLNDTVVDQ LKKGSNKLIV EIFADHPGQS GGG----EQ- --AKGGRGKK

RPRC012274-PA          GGGFATVSSS YKEQLNNLMT TLKSTQPHFV RCIIPNELKQ PGVIDSHLVM
CPIJ000848-RA          GAGFATVSSA YKEQLNNLIR TLCSTSPHFV RCIIPNELKQ TGLIDAHLVM
AGAP010147-PA          GAGFATVSSS YKEQLNNLMT TLKSTQPHFV RCIIPNEMKT AGVVDAHLVM
CPIJ000853-RA          GAGFATVSSS YKEQLNNLMT TLKSTQPHFV RCIIPNELKQ TGLIDAHLVM
AAEL005733-RB          GAGFATVSSS YKEQLNNLMT TLKSTQPHFV RCIIPNELKQ TGLIDAHLVM
CPIJ000849-RA          GAGFATVSSA YKEQLNNLIR TLCSTSPHFV RCIIPNELKQ TGLIDAHLVM
AAEL005656-RA          GAAFATVSSA YKEQLNNLMN TLMSTSPHFV RCIIPNELKQ TGLIDAKLVM
MyoSex                 GAAFATVSSA YKEQLNNLMT TLQSTSPHFV RCIIPNELKQ TGLIDAKLVM
XP_001814139.1_Tc      GGGFATVSSA YKEQLNNLMT TLRSTQPHFV RCIIPNELKQ PGVIDSHLVM
GMOY005703-PA          GGGFATVSSG YKEQLNSLMT TLHSTQPHFV RCIIPNEMKQ PGVVDAHLVM
FBpp0080463            GGGFATVSSA YKEQLNSLMT TLRSTQPHFV RCIIPNEMKQ PGVVDAHLVM
BAG30740.1             GGGFATISSA YREQLNNLMT TLRSTQPHFV RCIIPNELKQ AGLIDSHLVM
LLOTMP009501-PA        GGGFATVSSS YKEQLNNLMT TLRSTQPHFV RCIIPNELKQ PGVIDSHLVM

RPRC012274-PA          HQLTCNGVLE GIRICRKGFP NRMVYPDFKL RYMILAPATM AKEADPKVAA
CPIJ000848-RA          HQLTCNGVLE GIRICRKGFP NRMNYPDFKQ RYLILAPAAM QAEPEGKVAA
AGAP010147-PA          HQLTCNGVLE GIRICRKGFP NRMMYPDFKL RYKILCPQLI KEPCSPEKVT
CPIJ000853-RA          HQLTCNGVLE GIRICRKGFP NRMMYPDFKL RYLILAPAAM MAEKEGKNAA
AAEL005733-RB          HQLTCNGVLE GIRICRKGFP NRMMYPDFKL RYKILNPKAA EEQKEPKNVA
CPIJ000849-RA          HQLTCNGVLE GIRICRKGFP NRMNYPDFKQ RYLILAPAAM SAEPEGKKAA
AAEL005656-RA          HQLTCNGVLE GIRICRKGFP NRMMYSDFKQ RYLILAPAAM QAEAEPKKAA
MyoSex                 HQLTCNGVLE GIRICRKGFP NRMMYPDFKQ RYLILAPAAM QAEQDSKKAA
XP_001814139.1_Tc      HQLTCNGVLE GIRICRKGFP NRMVYPDFKL RYMILAPATM AAEKDPKEAA
GMOY005703-PA          HQLTCNGVLE GIRICRKGFP NRMMYPDFKQ RYMILAPAVM AAEKQPKKAA
FBpp0080463            HQLTCNGVLE GIRICRKGFP NRMMYPDFKM RYMILAPAIM AAEKVAKNAA
BAG30740.1             HQLTCNGVLE GIRICRKGFP NRMVYPDFKL RYKILAPQAA DKETDPKKVA
LLOTMP009501-PA        HQLTCNGVLE GIRICRKGFP NRMVYPDFKL RYKILCLSLI KEPCKPEKAS

RPRC012274-PA          AKCLKEVELD AESYRIGNTK ATGVDKEPDP KKAAAVVLEA TTLDPDQ---
CPIJ000848-RA          EKCMEAVALD PDLF------ ---------- ---------- ----------
AGAP010147-PA          QIVLTHIQLP EEQF------ ---------- ---------- ----------
CPIJ000853-RA          QKCFDAIGLD PESY------ ---------- ---------- ----------
AAEL005733-RB          DVILTSIGLD TESY------ ---------- ---------- ----------
CPIJ000849-RA          EKCMEAVALD PDLF------ ---------- ---------- ----------
AAEL005656-RA          EKCFEAIQLD PDSY------ ---------- ---------- ----------
MyoSex                 EKCFEAIQLD PDSY------ ---------- ---------- ----------
XP_001814139.1_Tc      RKCLEEVGLD PDSY------ ---------- ---------- ----------
GMOY005703-PA          EKCLESVGLD PDLYQILSPA AIKGVADHKK ASAILLESTS LDPDMYRTGH
FBpp0080463            GKCLEAVGLD PDMY------ ---------- ---------- ----------
BAG30740.1             QVILDATGLD PESY------ ---------- ---------- ----------
LLOTMP009501-PA        EIILNHVQLA EDQF------ ---------- ---------- ----------

RPRC012274-PA          ---------- ---------- ---------- ---------- ----------
CPIJ000848-RA          ---------- ---------- ---------- ---------- ----------
AGAP010147-PA          ---------- ---------- ---------- ---------- ----------
CPIJ000853-RA          ---------- ---------- ---------- ---------- ----------
AAEL005733-RB          ---------- ---------- ---------- ---------- ----------
CPIJ000849-RA          ---------- ---------- ---------- ---------- ----------
AAEL005656-RA          ---------- ---------- ---------- ---------- ----------
```

```
MyoSex                      ---------- ---------- ---------- ---------- ----------
XP_001814139.1_Tc           ---------- ---------- ---------- ---------- ----------
GMOY005703-PA               TKARYFISVV SLRTPSSLSL ISLLSFFLLI LWNAKPKMGK SNLTAYVCLK
FBpp0080463                 ---------- ---------- ---------- ---------- ----------
BAG30740.1                  ---------- ---------- ---------- ---------- ----------
LLOTMP009501-PA             ---------- ---------- ---------- ---------- ----------


RPRC012274-PA               ---------- ---------- ---------- ---------- ---------Y
CPIJ000848-RA               ---------- ---------- ---------- ---------- ----------
AGAP010147-PA               ---------- ---------- ---------- ---------- ----------
CPIJ000853-RA               ---------- ---------- ---------- ---------- ----------
AAEL005733-RB               ---------- ---------- ---------- ---------- ----------
CPIJ000849-RA               ---------- ---------- ---------- ---------- ----------
AAEL005656-RA               ---------- ---------- ---------- ---------- ----------
MyoSex                      ---------- ---------- ---------- ---------- ----------
XP_001814139.1_Tc           ---------- ---------- ---------- ---------- ----------
GMOY005703-PA               RLIYQILNPK GILGQGDPKK CSKILLESTA LDQDQFRLGN TKACSIFLFY
FBpp0080463                 ---------- ---------- ---------- ---------- ----------
BAG30740.1                  ---------- ---------- ---------- ---------- ----------
LLOTMP009501-PA             ---------- ---------- ---------- ---------- ----------


RPRC012274-PA               KILNAKGVKD YMSAEEATKT ILEGNQVDPE QYRMGHSKRT NHFCLTQRKN
CPIJ000848-RA               ---------- ---------- ---------- --RIGHTK-- ----------
AGAP010147-PA               ---------- ---------- ---------- --RMGKTK-- ----------
CPIJ000853-RA               ---------- ---------- ---------- --RIGHTK-- ----------
AAEL005733-RB               ---------- ---------- ---------- --RLGHTK-- ----------
CPIJ000849-RA               ---------- ---------- ---------- --RIGHTK-- ----------
AAEL005656-RA               ---------- ---------- ---------- --RIGHTK-- ----------
MyoSex                      ---------- ---------- ---------- --RIGHTK-- ----------
XP_001814139.1_Tc           ---------- ---------- ---------- --RIGHTK-- ----------
GMOY005703-PA               KIMCPKKLLG VDDPKKATQV IINYIDLPDD QFRLGNTK-- ----------
FBpp0080463                 ---------- ---------- ---------- --RIGHTK-- ----------
BAG30740.1                  ---------- ---------- ---------- --RLGHTK-- ----------
LLOTMP009501-PA             ---------- ---------- ---------- --RLGKTK-- ----------


RPRC012274-PA               IISPCFNSLI PPPNQSLILR TNRTDFLPRK ERMPKKYLVF FRAGVLGQLE
CPIJ000848-RA               ---------- ---------- ---------- --------IF FRAGVLGQME
AGAP010147-PA               ---------- ---------- ---------- --------VF FRAGVLGQME
CPIJ000853-RA               --ARIYHFFY ICVVLPSFPR SCPSTSDQYL SGLTADQLVF FRAGVLGQME
AAEL005733-RB               ---------- ---------- ---------- --------VF FRAGVLGQME
CPIJ000849-RA               ---------- ---------- ---------- --------IF FRAGVVGQME
AAEL005656-RA               ---------- ---------- ---------- --------VF FRAGVLGQME
MyoSex                      ---------- ---------- ---------- --------VF FRAGVLGQME
XP_001814139.1_Tc           ---------- ---------- ---------- --------VF FRAGVLGQME
GMOY005703-PA               ---------- ---------- ---------- --------VF FRAGVLGQME
FBpp0080463                 ---------- ---------- ---------- --------VF FRAGVLGQME
BAG30740.1                  ---------- ---------- ---------- --------VF FRAGVLGQME
LLOTMP009501-PA             ---------- ---------- ---------- --------VF FRAGVLGQME


RPRC012274-PA               EMRDDRLSKI MGWLQSYIRD -PKKSSCRVS LEAELSLQVV QRNLRRYLQL
CPIJ000848-RA               EFRDERLSKI MTWVQSWCRG FLDRKEFKKA QLQRVALEVI QRNLRKYLKL
AGAP010147-PA               EFRDERLSKI MSWMQAWCRG YLSRKEFKKM QEQRVSLEIV QRNLRKYLKL
CPIJ000853-RA               EFRDDRLSKI MTWMQSWIRG YLSRKSFKKM QEQRVSLEIV QRNLRKYMKL
AAEL005733-RB               EFRDERLSKI MSWMQSWCRG YLARKEFKKM QEQRVALETV QRNLRKYMKL
CPIJ000849-RA               EFRDERLSKI MTWVQSWCRG FLDRKEFKKA QLQRVALEVI QRNLRKYLKL
AAEL005656-RA               DFRDQRLSKI MSWLQSWCRG YLTRIEFKKM QSQRTALEVV QRNLRKYLKL
MyoSex                      DFRDQRLSKI MSWMQSWCRG YLSRTEFKKM QSQRTALEVV QRNLRKYLKL
XP_001814139.1_Tc           ELRDERLGKI VTWMQSWVRG YLSRKEFKRL QEQRLALQVC QRNLRKYLKL
GMOY005703-PA               EFRDERLGKI MSWMQAWARG YLARKGFKKL QEQRVALKVV QRNLRKYLQL
FBpp0080463                 EFRDERLGKI MSWMQAWARG YLSRKGFKKL QEQRVALKVV QRNLRKYLQL
BAG30740.1                  ELRDDRLSKI VSWMQAYIRG YLSRKEYKKL QEQRLALQVV QRNLRKYLQL
LLOTMP009501-PA             ELRDERLSKI MSWLQSWIRG YLARKEYQKM QEQRLALQVV QRNLRKYLQL
```

```
RPRC012274-PA          RTWPWWKMWS KVKPLLNVAN VEEEMRVNDI DMLIKPTNRE ----------
CPIJ000848-RA          RTWAWWKLWG KVKPLLNVSR VEDQIA---- ---------- ----------
AGAP010147-PA          RTWAWWKLWQ KVKPLLNVSR VEDQIA---- ---------- ----------
CPIJ000853-RA          RTWAWWKLWQ KVKPLLNVSR VEDQIAVSIR QRRPLPATAK AAAAVKAAND
AAEL005733-RB          RTWAWWKLWQ KVKPLLNVSR VEDQIA---- ---------- ----------
CPIJ000849-RA          RTWAWWKLWG KVKPLLNVSR VEDQIA---- ---------- ----------
AAEL005656-RA          RTWAWWKLWQ KVKPLLNVSR VEDQIA---- ---------- ----------
MyoSex                 RTWAWWKLWQ KVKPLLNVSR VEDQIA---- ---------- ----------
XP_001814139.1_Tc      RTWPWYKLWQ KVKPLLNVTR IEDEIA---- ---------- ----------
GMOY005703-PA          RTWPWYKLWQ K--------- ---------- ---------- ----------
FBpp0080463            RTWPWYKLWQ KVKPLLNVSR IEDEIA---- ---------- ----------
BAG30740.1             RTWPWWKLWQ RVKPLLNVTR IEDEIA---- ---------- ----------
LLOTMP009501-PA        RTWPWYKLWT KVKPLLNVTR IEDEIA---- ---------- ----------

RPRC012274-PA          ---------- ---------- ---------- ---------- ----------
CPIJ000848-RA          ---------- ---------- ---------- ---------- ----------
AGAP010147-PA          ---------- ---------- ---------- ---------- ----------
CPIJ000853-RA          DARFRKLKRS ALTNVVTHRT KISFPSDYVG DLRPPPIFHG NFHLVLNNYS
AAEL005733-RB          ---------- ---------- ---------- ---------- ----------
CPIJ000849-RA          ---------- ---------- ---------- ---------- ----------
AAEL005656-RA          ---------- ---------- ---------- ---------- ----------
MyoSex                 ---------- ---------- ---------- ---------- ----------
XP_001814139.1_Tc      ---------- ---------- ---------- ---------- ----------
GMOY005703-PA          ---------- ---------- ---------- ---------- ----------
FBpp0080463            ---------- ---------- ---------- ---------- ----------
BAG30740.1             ---------- ---------- ---------- ---------- ----------
LLOTMP009501-PA        ---------- ---------- ---------- ---------- ----------

RPRC012274-PA          ---------- ---------- ---------- ---------- ----------
CPIJ000848-RA          ---------- ---------- ---------- ---------- ----------
AGAP010147-PA          ---------- ---------- ---------- ---------- ----------
CPIJ000853-RA          RRTTALQTLL ISRSCAKRKP LAELAIFGHF RIKISHHDNY NTCHHQRKIV
AAEL005733-RB          ---------- ---------- ---------- ---------- ----------
CPIJ000849-RA          ---------- ---------- ---------- ---------- ----------
AAEL005656-RA          ---------- ---------- ---------- ---------- ----------
MyoSex                 ---------- ---------- ---------- ---------- ----------
XP_001814139.1_Tc      ---------- ---------- ---------- ---------- ----------
GMOY005703-PA          ---------- ---------- ---------- ---------- ----------
FBpp0080463            ---------- ---------- ---------- ---------- ----------
BAG30740.1             ---------- ---------- ---------- ---------- ----------
LLOTMP009501-PA        ---------- ---------- ---------- ---------- ----------

RPRC012274-PA          ---------- ---------- ---------- ---------- ----------
CPIJ000848-RA          ---------- ---------- ---------- ---------- ----------
AGAP010147-PA          ---------- ---------- ---------- ---------- ----------
CPIJ000853-RA          STCLTSSPSV ARRSKMENKP EPDVEVTFNF NFDQSNNAEP NAPKVSATAA
AAEL005733-RB          ---------- ---------- ---------- ---------- ----------
CPIJ000849-RA          ---------- ---------- ---------- ---------- ----------
AAEL005656-RA          ---------- ---------- ---------- ---------- ----------
MyoSex                 ---------- ---------- ---------- ---------- ----------
XP_001814139.1_Tc      ---------- ---------- ---------- ---------- ----------
GMOY005703-PA          ---------- ---------- ---------- ---------- ----------
FBpp0080463            ---------- ---------- ---------- ---------- ----------
BAG30740.1             ---------- ---------- ---------- ---------- ----------
LLOTMP009501-PA        ---------- ---------- ---------- ---------- ----------

RPRC012274-PA          ---------- ---------- --KLQKLEEL VAQTQAALEK EEKARKEVEA
CPIJ000848-RA          ---------- ---------- -----KLEEK AQHATEALEK EEKLRKELEA
AGAP010147-PA          ---------- ---------- -----KLEEK ATKAQEAYEK EEKLRKELEA
CPIJ000853-RA          ASDFDEENCV VRASETESNS SSKLQKLEET AKKAQDDLEK ETKLRQELEA
```

```
AAEL005733-RB          ---------- ---------- -----ELESK AQKAQEAFEK EEKARKELEA
CPIJ000849-RA          ---------- ---------- -----KLEEK AQHATEALEK EEKLRKELEA
AAEL005656-RA          ---------- ---------- -----KLEEK AQKATEAFEK EEKLRKELEA
MyoSex                 ---------- ---------- -----KLEEK AQKATEAFEK EEKLRKELEA
XP_001814139.1_Tc      ---------- ---------- -----KLEEK AAKAQEAYER EAKAKKELEG
GMOY005703-PA          ---------- ---------- -----RLEEK AKKAEEAHAA EVKVRKELEA
FBpp0080463            ---------- ---------- -----RLEEK AKKAEELHAA EVKVRKELEA
BAG30740.1             ---------- ---------- -----KLEEK AQKAQEAFEK EEKLRKEVEA
LLOTMP009501-PA        ---------- ---------- -----KLEEK AKKAEEAFAK EEKLRKELET


RPRC012274-PA          LNAKLIQEKT DLLRSLEGEK GSLSSFQEKV AKLQAQKTDL ESQL--LVKR
CPIJ000848-RA          MNSKLLAEKT ALLDSLSGEK GALQEYQEKA AKLTAQKNDL ENQLRDTQER
AGAP010147-PA          LNSKLLAEKT ALLDSLSGEK GALQEYQEKA AKLTAQKNDL ENQLRDTQER
CPIJ000853-RA          LNSKLLAEKT ALLDSLSGEK GALQDFQEKT AKLQAQKADV ENQLRDTQER
AAEL005733-RB          LNSKLLAEKT ALLDSLSGEK GALQDFQEKT AKLTAQKNDL ENQLRDTQER
CPIJ000849-RA          MNSKLLAEKT ALLDSLSGEK GALQEYQEKA AKLTAQKNDL ENQLRDTQER
AAEL005656-RA          LNSKLLAEKT ALLDSLSGEK GALQEYQEKA AKLTAQKNDL ENQLRDTQER
MyoSex                 LNSKLLAEKT ALLDSLSGEK GALQEYQEKA AKLTAQKNDL ENQLRDTQER
XP_001814139.1_Tc      LYSKLLAEKT ELLSSLEGEK GSLSEVQERA NKLQAQKSDL ESQLSETQDR
GMOY005703-PA          LNAKLLAEKT ALLDSLSGEK GALQDYQERC AKLQAQKNDL ENQLRDIQDR
FBpp0080463            LNAKLLAEKT ALLDSLSGEK GALQDYQERN AKLTAQKNDL ENQLRDIQER
BAG30740.1             LNAKLLEEKT ALLSTLEGEK GSLSETQERA NKLQAQKNDL ENQLRDTQDR
LLOTMP009501-PA        LNSKLLAEKT ALLDSLSGEK GQLSEFQEKC AKLTAQKNDL DNQLRDTQER


RPRC012274-PA          LQTEEDARNQ LFQQKKKLEQ ESAGLKKDIE DLELSMQKTD QDKASKEHQI
CPIJ000848-RA          LAQEEDARNQ LFQTKKKLEQ EISGQKKDAE DLELQIQKIE HDKASKDHTI
AGAP010147-PA          LAQEEDARNQ LFQTKKKLEQ EIGSQKKDAE DLELQIQKIE QDKASKDHQI
CPIJ000853-RA          LTQEEDARNQ LFQQKKKLEQ EISGQKKDAE DLELQIQKIE QDKASKDHQI
AAEL005733-RB          LSQEEDARNQ LMQTKKKLEQ EIGGQKKDAE DLELQIQKIE QDKASKDHQI
CPIJ000849-RA          LAQEEDARNQ LFQTKKKLEQ EISGQKKDAE DLELQIQKTE QDKASKDHQI
AAEL005656-RA          LAQEEDARNQ LFQTKKKLEQ EISGQKKDAE DLELQIQKIE QDKASKDHQI
MyoSex                 LAQEEDARNQ LFQTKKKLEQ EISGQKKDAE DLELQIQKIE QDKASKDHQI
XP_001814139.1_Tc      LSQEEDARNQ LMQQKKKLEQ EISGYKKDIE DLELNLQKSE QDKATKDHQI
GMOY005703-PA          LTQEEDARNQ LFQQKKKADQ EISGLKKDIE DLELNVQKSE QDKATKDHQI
FBpp0080463            LTQEEDARNQ LFQQKKKADQ EISGLKKDIE DLELNVQKAE QDKATKDHQI
BAG30740.1             LTQEEDARNQ LFQAKKKLEQ EVSGLKKDIE DLELSVQKSE QDKATKDHQI
LLOTMP009501-PA        LSQEEDARNQ LFQTKKKLEQ EVSGLKKDIE DLELNTQKSE QDKATKDHQI


RPRC012274-PA          RNLNDEIAHQ DELINKLNKE KKIQSEHNQK TAEELQAAED KINHLTKVKA
CPIJ000848-RA          RNLNDEIAHQ DELINKLNKE KKMQGEVNQR NGEELQAAED KVNHLNKVKA
AGAP010147-PA          RNLNDEIAHQ DELINKLNKE KKMQGEVNQK TAEELQAAED KVNHLNKVKA
CPIJ000853-RA          RNLNDEIAHQ DELINKLNKE KKMSGEVNQK TAEELQAAED KVNHLNKVKA
AAEL005733-RB          RNLNDEIAHQ DELINKLNKE KKMQGEVNQK TAEELQAAED KVNHLNKVKA
CPIJ000849-RA          RNLNDEIAHQ DELINKLNKE KKMQGEVNQK TAEELQAAED KVNHLNKVKA
AAEL005656-RA          RNLNDEIAHQ DELINKLNKE KKMQGEVNQK TAEELQAAED KVNHLNKVKA
MyoSex                 RNLNDEIAHQ DELINKLNKE KKMQGEVNQK TAEELQAAED KVNHLNKVKA
XP_001814139.1_Tc      RNLNDEIAHQ DELINKLNKE KKLSGENSQR VSEELQAAED KVNHLNKVKA
GMOY005703-PA          RNLNDEIAHQ DELINKLNKE KKMQGEANQK TGEELQAAED KINHLNKVKA
FBpp0080463            RNLNDEIAHQ DELINKLNKE KKMQGETNQK TGEELQAAED KINHLNKVKA
BAG30740.1             RNLNDEIAHQ DELINKLNKE KKMQGESNQK TSEELQAAED KVNHLNKVKQ
LLOTMP009501-PA        RNLNDEIAHQ DELINKLNKE KKMQGETNQK TAEELQAAED KINHLNKVKA


RPRC012274-PA          KLEQTLDELE DSLEREKKLR GDVEKAKRKT EGDLKLTQEA VADLERNKKE
CPIJ000848-RA          KLEQTLDELE ESLDREKRLR GDVDKNKRKV DGELKLTREA VLDLERNRKE
AGAP010147-PA          KLEQTLDELE DSLEREKKLR GDVEKAKRKV EGDLKLTQEA VADLERNKKE
CPIJ000853-RA          KLEQTLDELE DSLEREKKLR GDVEKAKRKV EGDLKLTQEA VADLERNKKE
AAEL005733-RB          KLEQTLDELE DSLEREKKLR GDVEKAKRKV EGDLKLTQEA VADLERNKKE
CPIJ000849-RA          KLEQTLDELE DSLEREKKLR GDVDKAKRKV EGDLKLTQEA VSDLERYKKE
AAEL005656-RA          KLEQTLDELE DSLEREKKLR GDVEKAKRKV EGDLKLTQEA VSDLERNKKE
MyoSex                 KLEQTLDELE DSLEREKKLR GDVEKAKRKV EGDLKITQEA VSDLERNKKE
XP_001814139.1_Tc      KLEQTLDELE DSLEREKKLR GDVEKSKRKV EGDLKLTQEA VADLERNKKE
GMOY005703-PA          KLEQTLDELE DSLEREKKMR GDVEKGKRKV EGDLKLTQEA VADLERNKKE
```

```
FBpp0080463              KLEQTLDELE DSLEREKKVR GDVEKSKRKV EGDLKLTQEA VADLERNKKE
BAG30740.1               KLEQTLDELE DSLEREKKLR ADVEKQRRKV EGDLKLTQEA VTDLERNKKE
LLOTMP009501-PA          KLEQTLDELE DSLEREKKVR GDVEKSKRKV EGDLKLTQEA VADLERNKKE


RPRC012274-PA            LEQTIQRKDK EIASLTAKLE DEQSIVNKTG KQIKELQSRI EELEEEVEAE
CPIJ000848-RA            LEQTILRKDK EISALSANVE TEQNLVGKLQ KQIKELQGRI DELQDEVESE
AGAP010147-PA            LEQTVLRKDK EISALSAKLE DEQSLVGKLQ KQIKELQARI EELEEEVEAE
CPIJ000853-RA            LEQTIMRKDK EISALSAKLE DEQSLVGKLQ KQIKELQGRI EELEEEVEAE
AAEL005733-RB            LEQTIMRKDK EISALSAKLE DEQSLVGKTQ KQIKELQGRI EELEEEVEAE
CPIJ000849-RA            LEQTILRKDK EISALSAKLE DEQNLVGKLQ KQIKELQGRI EELEEEVEAE
AAEL005656-RA            MDQTIMRKDK EISALSAKLE DEQNLVGKLQ KQIKELQGRI EELEEEVEAE
MyoSex                   LEQTIMRKDK EISALSAKLE DEQNLVGKLQ KQIKELQGRI EELEEEVEAE
XP_001814139.1_Tc        LEQTIQRKDK EISSLTAKLE DEQSVVGKLQ KQIKELQARI EELEEEVEAE
GMOY005703-PA            LEQTIQRKDK ELSSITAKLD DEQVVVSKHQ RQIKELQARI EELEEEVEAE
FBpp0080463              LEQTIQRKDK ELSSITAKLE DEQVVVLKHQ RQIKELQARI EELEEEVEAE
BAG30740.1               LEQTIQRKDK EISSLTAKLE DEQSLVSKLQ KQIKELQARI EELEEEVESE
LLOTMP009501-PA          LEQTIQRKDK EISSLTAKLE DEQSIVNKLQ KQIKELQSRI EELEEEVEAE


RPRC012274-PA            RQARGKAEKQ RADLARELEE LGERLEEAGG ATSAQIELNK KREAEMSKLR
CPIJ000848-RA            RQARAKAEKQ RADLARELED FGERLEEAGG ATSAQIDLNR KRDVELTKLR
AGAP010147-PA            RQARAKAEKQ RADLARELEE LGERLEEAGG ATSAQIELNK KREAELAKLR
CPIJ000853-RA            RQARAKAEKQ RADLARELEE LGERLEEAGG ATSAQIELNK KREAELAKLR
AAEL005733-RB            RQARAKAEKQ RADLARELEE LGERLEEAGG ATSAQIELNK KREAELAKLR
CPIJ000849-RA            RQARAKAEKQ RADLARELEE LGERLEEAGG ATSAQIELNK KREAELAKLR
AAEL005656-RA            RQARAKAEKQ RADLARELEE LGERLEEAGG ATSAQIELNK KREAELAKLR
MyoSex                   RQARAKAEKQ RADLARELEE LGERLEEAGG ATSAQIELNK KREAELAKLR
XP_001814139.1_Tc        RQARAKAEKQ RADLARELEE LGERLEEAGG ATSAQIELNK KREAELAKLR
GMOY005703-PA            RQARAKAEKQ RADLARELEE LGERLEEAGG ATSAQIELNK KREAELSKLR
FBpp0080463              RQARAKAEKQ RADLARELEE LGERLEEAGG ATSAQIELNK KREAELSKLR
BAG30740.1               RQARAKAEKQ RADLARELEE LGERLEEAGG ATSAQIELNK KREAELSKLR
LLOTMP009501-PA          RQARSKAEKQ RADLARELEE LGERLEEAGG ATSAQIELNK KREAELAKLR


RPRC012274-PA            RDLEEANIQH ESTLANLRKK HNDAVSEMGD QIDQLNKLKT KVEKEKCQYL
CPIJ000848-RA            RDLEEANIQH ESTLASLRKK HNDAVAEMAE QVEQLNKLKM KAEHDRANMY
AGAP010147-PA            RDLEEANIQH EGTLANLRKK HNDAVAEMAE QVDQLNKLKT KAEKERTQYF
CPIJ000853-RA            RDLEESNIQH EGTLANLRKK HNDAVAEMAE QVDQLNKLKT KAEHDRANMY
AAEL005733-RB            RDLEESNIQH EGTLANLRKK HNDAVAEMAE QVDQLNKLKT KAEKERSQYY
CPIJ000849-RA            ---------- ---------- ---------- ---------- -PEHDRANMY
AAEL005656-RA            RDLEEANIQH EGTLANLRKK HNDAVAEMAE QVDQLNKLKT KAEHDRANMY
MyoSex                   RDLEEANIQH EGTLANLRKK HNDAVAEMAE QVDQLNKLKT KAEHDRANMY
XP_001814139.1_Tc        RDLEESNIQH ESTLANLRKK HNDAVSEMGE QIDQLNKLKA KAERDRASIY
GMOY005703-PA            RDLEEANIQH ESTLANLRKK HNDAVAEMAE QVDQLNKLKA KAEHDRQTCH
FBpp0080463              RDLEEANIQH ESTLANLRKK HNDAVAEMAE QVDQLNKLKA KAEHDRQTCH
BAG30740.1               RDLEEANIQH ESTLANLRKK HNDAVAEMGE QLDQLNKLKA KAEKERSQYF
LLOTMP009501-PA          RDLEEANIQH ESTLANLRKK HNDAVAEMAE QVDQLNKLKA KAEKEKAQYF


RPRC012274-PA            CELNDVRASI DHLTN----- ---------- ---------- ----------
CPIJ000848-RA            NELNKTRSSC DQLAR----- ---------- ---------- ----------
AGAP010147-PA            AELNDARIGC DQLSN----- ---------- ---------- ----------
CPIJ000853-RA            NDLNNTRTAC DQLAR----- ---------- ---------- ----------
AAEL005733-RB            AEMNDARLSL DHMAN----- ---------- ---------- ----------
CPIJ000849-RA            NELNNTRSAC DTLAR----- ---------- ---------- ----------
AAEL005656-RA            NELNNTRSAC DQLSR----- ---------- ---------- ----------
MyoSex                   NELNNTRSAC DQLAR----- ---------- ---------- ----------
XP_001814139.1_Tc        TELQQTRSAV EQVGR----- ---------- ---------- ----------
GMOY005703-PA            NELNQTRSAC DQLAREKFLN YFIQQTATKW VSVMQRAYQI FYPLHNMAEK
FBpp0080463              NELNQTRTAC DQLGR----- ---------- ---------- ----------
BAG30740.1               SEVNDLRAGL DHLSN----- ---------- ---------- ----------
LLOTMP009501-PA          SEVNELRHSV DHLSN----- ---------- ---------- ----------


RPRC012274-PA            ---------- ---------- -EKAATEKVA KQLQHQINEV QGKLDEANRT
```

```
CPIJ000848-RA            ---------- ---------- -DKAGQEKIA KQLQHSLNHI HGKHDETNRT
AGAP010147-PA            ---------- ---------- -EKAAQEKIA KQLQHTLNEV QSKLDETNRT
CPIJ000853-RA            ---------- ---------- -EKASQEKIA KQLQHTLNEV QGKLDETNRT
AAEL005733-RB            ---------- ---------- -EKAAQEKVA KQLQHTLNEV QGKLDETNRT
CPIJ000849-RA            ---------- ---------- -EKAAQEKIA KQLQHTLNEV QGKLDETNRT
AAEL005656-RA            ---------- ---------- -EKAAQEKIA KQLQHTLNEV QGKLDETNRT
MyoSex                   ---------- ---------- -EKAAQEKIA KQLQHTLNEV QGKLDETNRT
XP_001814139.1_Tc        ---------- ---------- -EKAAVEKVS KQLQQQLNDV QGKLDETNRT
GMOY005703-PA            EKNEYYAQLN DLRSCVDHLT NEKAAQEKIA KQLQHTLNEV QSKLDETNRT
FBpp0080463              ---------- ---------- -DKAAQEKIA KQLQHTLNEV QSKLDETNRT
BAG30740.1               ---------- ---------- -EKAAQEKVV KQLQHNLNEV QNKADEANRT
LLOTMP009501-PA          ---------- ---------- -EKASQEKIA KQLQHTLNDV QGKLDETNRT

RPRC012274-PA            LNDFDAAKKK LSIENSDLLR QLEEAESQVS QLSKIKISLT TQLEDTKRLA
CPIJ000848-RA            LNDFDASKKK LSVENSDLLR RLETAESQVA QLSKLKISLS QQLEDTKRLA
AGAP010147-PA            LNDFDASKKK LSIENSDLLR QLEDAESQVS QLSKIKISLT QQLEDTKRLA
CPIJ000853-RA            LNDFDTSKKK LSIENSDLLR QLEDAESQVS QLSKIKISLT QQLEDTKRLA
AAEL005733-RB            LNDFDSAKKK LSIENSDLLR QLEDAESQVS QLSKIKISLT QQLEDTKRLA
CPIJ000849-RA            LNDFDASKKK LSIENSDLLR QLEDAESQVS QLSKIKISLS QQLEDTKRLA
AAEL005656-RA            LNDFDAAKKK LSIENSDLLR QLEDAESQVS QLSKIKISLT QQLEDTKRLA
MyoSex                   LNDFDAAKKK LSIENSDLLR QLEDAESQVS QLSKIKISLT QQLEDTKRLA
XP_001814139.1_Tc        LNDFDAAKKK LSIENSDLLR QLEEAESQVS QLSKIKVSLT TQLEDTKRLA
GMOY005703-PA            LNDFDAAKKK LSIENSDLLR QLEEAESQVS QLSKIKISLT TQLEDTKRLA
FBpp0080463              LNDFDASKKK LSIENSDLLR QLEEAESQVS QLSKIKISLT TQLEDTKRLA
BAG30740.1               LNDLDAAKKK LSIENSDLLR QLEEAESQVS QLSKIKVSLT TQLEDTKRLA
LLOTMP009501-PA          LNDFDAAKKK LSIENSDLLR QLEEAESQVS QLSKIKISLT TQLEDTKRLA

RPRC012274-PA            DEEARERATL LGKFRNLEHD LDNLREQVEE EAEAKADIQR QLSKANAEAQ
CPIJ000848-RA            DEESRERATL LGKFRNLEHD LDNLRDQVEE EAEGKGDIRR QLSKTYAEAQ
AGAP010147-PA            DEEARERATL LGKFRNLEHD LDNLREQVEE EAEGKGDIQR QLSKANAEAQ
CPIJ000853-RA            DEESRERATL LGKFRNLEHD LDSLREQVEE EAEGKGDIQR QLSKANAEAQ
AAEL005733-RB            DEESRERATL LGKFRNLEHD LDSLREQVEE EAEGKADIQR QLSKANAEAQ
CPIJ000849-RA            DEESRERATL LGKFRNLEHD LDNLREQVEE EAEGKGDIQR QLSKSNAEAQ
AAEL005656-RA            DEEARERATL LGKFRNLEHD LDNLREQVEE EAEGKGDIQR QLSKANAEAQ
MyoSex                   DEEARERATL LGKFRNLEHD LDNLREQVEE EAEGKGDIQR QLSKANAEAQ
XP_001814139.1_Tc        DEEGRERATL LGKFRNLEHD LDNIREQVEE EAEAKADIQR QLSKANAEAQ
GMOY005703-PA            DEEARERATL LGKFRNLEHD LDNLREQVEE EAEGKADLQR QLSKANAEAQ
FBpp0080463              DEESRERATL LGKFRNLEHD LDNLREQVEE EAEGKADLQR QLSKANAEAQ
BAG30740.1               DEEARERATL LGKFRNLEHD LDNIREQVEE EAEGKADLQR QLSKANAEAQ
LLOTMP009501-PA          DEESRERATL LGKFRNLEHD LDNLREQVEE EAEGKADLQR QLSKANAESQ

RPRC012274-PA            LWRSKYESEG IARAEELEEA KRKLQARLAE AEETIESLNQ KVIALEKTKQ
CPIJ000848-RA            LWRSRYETEG VSRAEELEEG KRKLQARLAE AEETIESLTQ KVIALEKTKQ
AGAP010147-PA            LWRSKYESEG VARAEELEEA KRKLQARLAE AEETIESLNQ KCIALEKTKQ
CPIJ000853-RA            LWRTKYESEG VARAEELEEA KRKLQARLAE AEETIESLNQ KCIALEKTKQ
AAEL005733-RB            LWRTKYESEG VARAEELEEA KRKLQARLAE AEETIESLNQ KCVALEKTKQ
CPIJ000849-RA            LWRTKYESEG VARAEELEEA KRKLQARLAE AEETIESLNQ KCIALEKTKQ
AAEL005656-RA            LWRSKYESEG VARAEELEEA KRKLQARLAE AEETIESLNQ KCVALEKTKQ
MyoSex                   LWRSKYESEG VARAEELEEA KRKLQARLAE AEETIESLNQ KCVALEKTKQ
XP_001814139.1_Tc        LWRQKYESEG VAKSEELEEA KRKLQARLAE AEETIESLNQ KVVALEKTKQ
GMOY005703-PA            LWRSKYESDG VARSEELEEA KRKLQARLAE AEETIESLNQ KCIGLEKTKQ
FBpp0080463              VWRSKYESDG VARSEELEEA KRKLQARLAE AEETIESLNQ KCIGLEKTKQ
BAG30740.1               LWRSKYESEG VARSEELEEA KRKLQARLAE AEETIESLNQ KVVALEKTKQ
LLOTMP009501-PA          LWRSKYESDG VARSEELEEA KRKLQARLAE AEETIESLNQ KVVALEKTKQ

RPRC012274-PA            RLATEVEDLQ LEVDRANAIA NAAEKKAKAI DKIIGEWKLK VDDLAAELDA
CPIJ000848-RA            RLSTEIEDLQ LEVGRSTVIA NSAEKKHRSF DKIIGEWKLK VDDLAGELDA
AGAP010147-PA            RLATEVEDLQ LEVDRASSIA NAAEKKQKAF DKIIGEWKLK VDDLAAELDA
CPIJ000853-RA            RLSTEVEDLQ LEVDRATSIA NSAEKKQKAF DKIIGEWKLK VDDLAAELDA
AAEL005733-RB            RLSTEVEDLQ LEVDRATSIA NAAEKKQKAF DKIIGEWKLK VDDLAAELDA
CPIJ000849-RA            RLSTEVEDLQ LEVDRATTIA NSAEKKQKAF DKIIGEWKLK VDDLAAELDA
AAEL005656-RA            RLSTEVEDLQ LEVDRATSIA NAAEKKQKAF DKIIGEWKLK VDDLAAELDA
```

```
MyoSex                     RLSTEVEDLQ LEVDRATSIA NAAEKKQKAF DKIIGEWKLK VDDLAAELDA
XP_001814139.1_Tc          RLATEVEDLQ IEVDRANAIA NAAEKKQKAF DKIIGEWKLK VDDLAAELDA
GMOY005703-PA              RLSTEVEDLQ LEVDRANAIA NAAEKKQKAF DKIIGEWKLK VDDLAAELDA
FBpp0080463                RLSTEVEDLQ LEVDRANAIA NAAEKKQKAF DKIIGEWKLK VDDLAAELDA
BAG30740.1                 RLATEVEDLQ LEVDRATAIA NAAEKKQKAF DKIIGEWKLK VDDLAAELDA
LLOTMP009501-PA            RLATEVEDLQ LEVDRANAIA SAAEKKQKAF DKIIGEWKLK VDDLAAELDA

RPRC012274-PA              SQKECRNYST ELFRLKGAYE EGQEQLEAVR RENKNLADEV KDLLDQIGEG
CPIJ000848-RA              SQKECRNYST ELFRLKGAYE EGQEQLEAVR RENKNLADEV KDLLDQIGEG
AGAP010147-PA              SQKECRNYST ELFRLKGAYE EGQEQLEAVR RENKNLADEV KDLLDQIGEG
CPIJ000853-RA              SQKECRNYST ELFRLKGAYE EGQEQLEAVR RENKNLADEV KDLLDQIGEG
AAEL005733-RB              SQKECRNYST ELFRLKGAYE EGQEQLEAVR RENKNLADEV KDLLDQIGEG
CPIJ000849-RA              SQKECRNYST ELFRLKGAYE EGQEQLEAVR RENKNLADEV KDLLDQIGEG
AAEL005656-RA              SQKECRNYST ELFRLKGAYE EGQEQLEAVR RENKNLADEV KDLLDQIGEG
MyoSex                     SQKECRNYST ELFRLKGAYE EGQEQLEAVR RENKNLADEV KDLLDQIGEG
XP_001814139.1_Tc          SQKECRNYST ELFRLKGAYE EGQEQLEAVR RENKNLADEV KDLLDQIGEG
GMOY005703-PA              SQKECRNYST ELFRLKGAYE EGQEQLEAVR RENKNLADEV KDLLDQIGEG
FBpp0080463                SQKECRNYST ELFRLKGAYE EGQEQLEAVR RENKNLADEV KDLLDQIGEG
BAG30740.1                 SQKECRNYST ELFRLKGAYE EGQEQLEAVR RENKNLADEV KDLLDQIGEG
LLOTMP009501-PA            SQKECRNYST ELFRLKGAYE EGQEQLEAVR RENKNLADEV KDLLDQIGEG

RPRC012274-PA              GRNIHEIEKQ RKRLEVEKDE LQAALEEAEA ALEQEENKVL RSQLELSQVR
CPIJ000848-RA              GRNIHEIEKS RKRLEAEKDE LQAALEEAEA ALEQEENKVL RAQLELSQVR
AGAP010147-PA              GRNIHEIEKS RKRLEAEKDE LQAALEEAEA ALEQEENKVL RAQLELSQVR
CPIJ000853-RA              GRNIHEIEKS RKRLEAEKDE LQAALEEAEA ALEQEENKVL RAQLELSQVR
AAEL005733-RB              GRNIHEIEKS RKRLEAEKDE LQAALEEAEA ALEQEENKVL RAQLELSQVR
CPIJ000849-RA              GRNIHEIEKS RKRLEAEKDE LQAALEEAEA ALEQEENKVL RAQLELSQVR
AAEL005656-RA              GRNIHEIEKS RKRLEAEKDE LQAALEEAEA ALEQEENKVL RAQLELSQVR
MyoSex                     GRNIHEIEKS RKRLEAEKGE LQAALEEAEA ALEQEENKVL RAQLELSQVR
XP_001814139.1_Tc          GRNIHEIEKA RKRLEAEKDE LQAALEEAEA ALEQEENKVL RSQLELSQVR
GMOY005703-PA              GRNIHEIEKA RKRLEAEKDE LQAALEEAEA ALEQEENKVL RAQLELSQVR
FBpp0080463                GRNIHEIEKA RKRLEAEKDE LQAALEEAEA ALEQEENKVL RAQLELSQVR
BAG30740.1                 GRNIHEIEKA RKRLEAEKDE LQAALEEAEA ALEQEENKVL RAQLELSQVR
LLOTMP009501-PA            GRNIHEIEKA RKRLEAEKDE LQAALEEAEA ALEQEENKVL RAQLELSRVR

RPRC012274-PA              QEIDRRIQEK EEEFENTRKN HQRALDSMQA SLEAEAKGKA EALRMKKKLE
CPIJ000848-RA              QEIDRRIQEK EEEFENTRKN HQRSLESVQA SHEADAKGKA EALRMKKKLE
AGAP010147-PA              QEIDRRIQEK EEEFENTRKN HQRALDSMQA SLEAEAKGKA EALRMKKKLE
CPIJ000853-RA              QEIDRRIQEK EEEFENTRKN HQRALDSMQA SLEAEAKGKA EALRMKKKLE
AAEL005733-RB              QEIDRRIQEK EEEFENTRKN HQRALDSMQA SLEAEAKGKA EALRMKKKLE
CPIJ000849-RA              QEIDRRIQEK EEEFENTRKN HQRALDSMQA SLEAEAKGKA EALRMKKKLE
AAEL005656-RA              QEIDRRIQEK EEEFENTRKN HQRALDSMQA SLEAEAKGKA EALRMKKKLE
MyoSex                     QEIDRRIQEK EEEFENTRKN HQRALDSMQA SLEAEAKGKA EALRMKKKLE
XP_001814139.1_Tc          QEIDRRIQEK EEEFENTRKN HQRALDSMQA SLEAEAKGKA EALRMKKKLE
GMOY005703-PA              QEIDRRIQEK EEEFENTRKN HQRALDSMQA SLEAEAKGKA EALRMKKKLE
FBpp0080463                QEIDRRIQEK EEEFENTRKN HQRALDSMQA SLEAEAKGKA EALRMKKKLE
BAG30740.1                 QEIDRRIQEK EEEFENTRKN HQRALDSMQA SLEAEAKGKA EALRMKKKLE
LLOTMP009501-PA            QEIDRRIQEK EEEFENTRKN HQRALDSMQA SLEAEAKGKA EALRMKKKLE

RPRC012274-PA              ADINELEIAL DHANKANAEA QKSIKKYQQQ LKDVQTALEE EQRARDDARE
CPIJ000848-RA              ADINELEIAL DHANKANAEA QKNIKRYQQE LQDVQGITQE EQRARDEARE
AGAP010147-PA              ADINELEIAL DHANKANAEA QKNIKRYQQQ LKDVQSALEE EQRARDDARE
CPIJ000853-RA              ADINELEIAL DHANKANAEA QKNIKRYQQQ MKDVQSALEE EQRARDDARE
AAEL005733-RB              ADINELEIAL DHANKANAEA QKNIKRYQQQ LKDVQSALEE EQRARDDARE
CPIJ000849-RA              ADINELEIAL DHANKANAEA QKNIKRYQQQ LKDTQGALEE EQRARDDARE
AAEL005656-RA              ADINELEIAL DHANKANAEA QKNIKRYQQQ LKDLQGALEE EQRARDDARE
MyoSex                     ADINELEIAL DHANKANAEA QKNIKRYQQQ LKDVQGALEE EQRARDDARE
XP_001814139.1_Tc          ADINELEIAL DHANKANAEA QKTIKRYQQQ LKDTQTALEE EQRARDEARE
GMOY005703-PA              ADINELEIAL DHANKANAEA QKNIKRYQQQ LKDLQTALEE EQRARDDARE
FBpp0080463                ADINELEIAL DHANKANAEA QKNIKRYQQQ LKDIQTALEE EQRARDDARE
BAG30740.1                 ADINELEIAL DHANKANAEA QKNIKRYQQQ IKDLQTALEE EQRARDDARE
LLOTMP009501-PA            ADINELEIAL DHANKANAEA QKNIKRYQQQ LKDVQTALEE EQRARDDARE
```

```
RPRC012274-PA          QLGIAERRAN ALGNELEESR TLLEQADRGR RQAEQELGDA HEQINELAAQ
CPIJ000848-RA          QLGISKRRAH ALQNELEESR VLLEHSDRSR RQAEQELSDA HEQLSEVSAE
AGAP010147-PA          QLGISERRAN ALQNELEESR TLLEQADRGR RQAEQELSDA HEQLNEVSAQ
CPIJ000853-RA          QLGISERRAN ALQNELEESR TLLEQADRGR RQAEQELGDA HEQLNDVSAQ
AAEL005733-RB          QLGISERRAN ALQNELEESR TLLEQADRGR RQAEQELSDA HEQLNEVSAQ
CPIJ000849-RA          QLGISERRAN ALQNELEESR TLLEQADRGR RQAEQELGDA HEQLNEVSAQ
AAEL005656-RA          QLGISERRAN ALQNELEESR TLLEQADRGR RQAEQELSDA HEQLNEVSGQ
MyoSex                 QLGISERRAN ALQNELEESR TLLEQADRGR RQAEQELSDA HEQLNEVSAQ
XP_001814139.1_Tc      QLGISERRAN ALQNELEESR TLLEQADRAR RQAEQELGDA HEQLNDLSAQ
GMOY005703-PA          QLGISERRAN ALQNELEESR TLLEQADRGR RQAEQELADA HEQLNEVSAQ
FBpp0080463            QLGISERRAN ALQNELEESR TLLEQADRGR RQAEQELADA HEQLNEVSAQ
BAG30740.1             QLGISERRAN ALQNELEESR TLLEQADRAR RQAEQELGDA HEQLNELSAQ
LLOTMP009501-PA        QLGISERRAN ALQNELEESR TLLEQADRGR RQAEQELGDA HEQLNELSAQ

RPRC012274-PA          ATSASAAKRK LEGELQTLHA DLDELLNEAK NSEEKAKKAM VDAARLADEL
CPIJ000848-RA          SASNAAAKRK RDSELHTLHS DLDELLNEAK NSEEKAKKAM IDAARLADEL
AGAP010147-PA          NASIAAAKRK LESELQTLHS DLDELLNEAK NSEEKAKKAM VDAARLADEL
CPIJ000853-RA          NASIAAAKRK LESELQTLHS DLDELLNEAK NSEEKAKKAM VDAARLADEL
AAEL005733-RB          NASIAAAKRK LESELQTLHS DLDELLNEAK NSEEKAKKAM VDAARLADEL
CPIJ000849-RA          NASIAAAKRK LESELQTLHS DLDELLNEAK NSEEKAKKAM VDAARLADEL
AAEL005656-RA          NASIGAAKRK LESELQTLHS DLDELLNEAK NSEEKAKKAM VDAARLADEL
MyoSex                 NASIAAAKRK LESELQTLHS DLDELLNEAK NSEEKAKKAM VDAARLADEL
XP_001814139.1_Tc      NSSLSAAKRK LETELQTLHS DLDELLNEAK NSEEKAKKAM VDAARLADEL
GMOY005703-PA          NASIAAAKRK LESELQTLHS DLDELLNEAK NSEEKAKKAM VDAARLADEL
FBpp0080463            NASISAAKRK LESELQTLHS DLDELLNEAK NSEEKAKKAM VDAARLADEL
BAG30740.1             SASLSAAKRK LESELQTLHS DLDELLNEAK NSEEKAKKAM VDAARLADEL
LLOTMP009501-PA        NASISAAKRK LESELQTLHS DLDELLNEAK NSEEKAKKAM VDAARLADEL

RPRC012274-PA          RAEQDHAQTQ EKLRKALETQ IKELQVRLDE AENNALKGGK KAIAKLEQRV
CPIJ000848-RA          RAEQDHAQTQ AKLRKALEVQ IIALQARLDD AESSTLKGGK KATQKQEDRI
AGAP010147-PA          RAEQDHAQTQ EKLRKALEQQ IKELQVRLDE AESNALKGGK KAIQKLEQRV
CPIJ000853-RA          RAEQDHAQSQ EKMRKALEQQ IKELQVRLDD AETNALKGGK KAIQKLEQRV
AAEL005733-RB          RAEQDHAQTQ EKLRKALEQQ IKELQVRLDD AETNALKGGK KAIQKLEQRV
CPIJ000849-RA          RAEQDHAQTQ EKLRKALEQQ IKELQVRLDD AETNALKGGK KAIQKMEQRV
AAEL005656-RA          RAEQDHAQTQ EKLRKALEQQ IKELQVRLDD AETNALKGGK KAIQKLEQRV
MyoSex                 RAEQDHAQTQ EKLRKALEQQ IKELQVRLDE AETNALKGGK KAIQKLEQRA
XP_001814139.1_Tc      RAEQDHAQTQ EKLRKALETQ IKDLQVRLDE AEANALKGGK KLIQKLEQRV
GMOY005703-PA          RAEQDHAQTQ EKLRKALEQQ IKELQVRLDE AEANALKGGK KAIQKLEQRC
FBpp0080463            RAEQDHAQTQ EKLRKALEQQ IKELQVRLDE AEANALKGGK KAIQKLEQRV
BAG30740.1             RAEQEHAQTQ EKLRKALEQQ IKELQVRLDE AEANALKGGK KAIQKLEQRV
LLOTMP009501-PA        RAEQDHAQTQ EKLRKALETQ IKELQVRLDE AEANALKGGK KAIQKLEQRV

RPRC012274-PA          RELENELDGE QRRHADAQKN LRKSERRIKE LSFQSDEDRK NHERMQDLVD
CPIJ000848-RA          RELEAALDSE QSKHNEAQKN LRKAERRVKE LSFQSEEDRK NHERTQDLVD
AGAP010147-PA          RELESELDSE QRRHADAQKN LRKSERRIKE LTFQSEEDRK NHERMQDLVD
CPIJ000853-RA          RELEAELDSE QRRHTDAQKN LRKSERRIKE LTFQSEEDRK NHERMQDLVD
AAEL005733-RB          RELESELDSE QRRHTDAQKN LRKSERRIKE LTFQSEEDRK NHERMQDLVD
CPIJ000849-RA          RELESELDNE QRRHADAQKN LRKSERRIKE LTFQSEEDRK NHERMQDLVD
AAEL005656-RA          REIETELDSE QRRHADAQKN LRKSERRIKE LTFQSEEDRK NHERMQDLVD
MyoSex                 RELESELDSE QRRHADAQKN LRKSERRIKE LTFQSEEDRK NHERMQDLVD
XP_001814139.1_Tc      RELENELDGE QRRHADAQKN LRKSERRIKE LSFQAEEDRK NHERMQDLVD
GMOY005703-PA          RELENELDGE QRRHADAQKN LRKSERRIKE LSFQSEEDRK NHERMQDLVD
FBpp0080463            RELENELDGE QRRHADAQKN LRKSERRVKE LSFQSEEDRK NHERMQDLVD
BAG30740.1             RELENELDGE QRRHADAQKN LRKSERRIKE LSFQAEEDRK NHERMQDLVD
LLOTMP009501-PA        RELENELDGE QRRHADAQKN LRKSERRIKE LTFQSEEDRK NHERMQDLVD

RPRC012274-PA          KLQQKIKTYK RQIEEAEEIA ALNLAKFRKA QQELEEAEER ADLAEQAVSK
CPIJ000848-RA          KLQQKSTTYK KQIEEAEEIA ALNLAKFRKA QLELEEAEER ANVAEQAATK
AGAP010147-PA          KLQQKIKTYK RQIEEAEEIA ALNLAKFRKA QQELEEAEER ADIAEQAATK
CPIJ000853-RA          KLQQKIKTYK RQIEEAEEIA ALNLAKFRKA QQELEEAEER ADIAEQTATK
```

```
AAEL005733-RB             KLQQKIKTYK RQIEEAEEIA ALNLAKFRKA QQELEEAEER ADIAEQAATK
CPIJ000849-RA             KLQQKIKTYK RQIEEAEEIA ALNLAKFRKA QQELEEAEER ADIAEQAATK
AAEL005656-RA             KLQQKIKTYK RQIEEAEEIA ALNLAKFRKA QQELEEAEER ADLAEQAATK
MyoSex                    KLQQKIKTYK RQIEEAEEIA ALNLAKFRKA QQELEEAEER ADIAEQAATK
XP_001814139.1_Tc         KLQQKIKTYK RQIEEAEEIA ALNLAKFRKA QQELEEAEER ADLAEQAIAK
GMOY005703-PA             KLQQKIKTYK RQIEEAEEIA ALNLAKFRKA QQELEEAEER ADLAEQAISK
FBpp0080463               KLQQKIKTYK RQIEEAEEIA ALNLAKFRKA QQELEEAEER ADLAEQAISK
BAG30740.1                KLQQKIKTYK RQIEEVEEIA ALNLAKFRKA QQELEEAEER ADLAEQAISK
LLOTMP009501-PA           KLQQKIKTYK RQIEEAEEIA ALNLAKFRKA QQELEEAEER ADLAEQAINK


RPRC012274-PA             FRTKGAGRAG SAAR-GLSPL AHRQQPVRPQ LDGSAFPPRF DLHPD-EF
CPIJ000848-RA             LRSR--GRAS STAQSGGGGS AR-------- ---------- --------
AGAP010147-PA             FRTKG-GRAG SVQR-GASPA PQRQPSAMPA LAGLNLPT-- --FDDHGF
CPIJ000853-RA             FRTKG-GRAG SVQR-GASPA PQRQ-SAMPS LAALGLPT-- --FDDHAF
AAEL005733-RB             FRTKG-GRAG SVQR-GASPA PQRQPSVMPG LAGLNFPT-- --FDDHGF
CPIJ000849-RA             FRSKG-GRGG SAGR-GASPA QQRQQPALPP LAALGLPS-- --FEDHAL
AAEL005656-RA             FRSKG-GRGG SAQR-GGSPA LSTV------ ---------- --------
MyoSex                    FRSKG-GRSG SVQR-GVSPA VSTV------ ---------- --------
XP_001814139.1_Tc         FRAK--GRGG SVAR-GGSPA PPRQRPQMDG L---TFPPRF DLAPD-EI
GMOY005703-PA             FRAK--GRGG SVGR-GASPA PRAM-SVRPQ LDGMAFPPRF DLAPENEF
FBpp0080463               FRAK--GRAG SVGR-GASPA I--------- ---------- --------
BAG30740.1                FRGK--GRAG STAR-GVSPA PQRTRPALDG FG--TFPPRF DLAPESDF
LLOTMP009501-PA           FRTKG-GRAG SIGR-GGSPA PRASARPFDG M---AFPPRF DLAPENDF


;

endblock;

prset aamodelpr = mixed;
mcmc ngen=1000000 stoprule=yes nchains=40 stopval=0.001;
sump burnin=25;
sumt burnin=25;
```
