# Supplementary material for: Insights into the Preservation of the Homomorphic Sex-Determining Chromosome of Aedes aegypti from the Discovery of a Male-Biased Gene Tightly Linked to the M-Locus
Source: Genome Biol Evol. 2014 Jan 6;6(1):179–91. doi: 10.1093/gbe/evu002 (PMC3914700; doi:10.1093/gbe/evu002)
Supplement: Supplementary Data [file supp_evu002_supplemental-table-S1-primer-supplemental.pdf]

Supplemental Table S1: Primer Sequences

Sequence Name	Primer Sequences
Myo-sex	F: CCTTCAAGCACACCGTTACA<br>R: TCACTATGCAGGAGTTGTTTCG<br>F: GATGCAACGCACAAAATGAG<br>R:TGTGCAGTGTATTTTTCCCTGA<br>F: AGGATGCTCGAAACCAGCTA<br>R: TGAGCAATTTCGTCGTTCAG<br>F: CTGAACGACGAAATTGCTCA<br>R: CTTGGCAGACAATGCTGAGA<br>F: CTCGAAACCAGCTATTCCAAAC<br>R: CTTTTTCGACATCACCACGTAA<br>F: TCAGAAAACTGCTGAAGAGCTG<br>R: ACGCTTCAAGTTCCTTACCAAA<br>F: CACATGGGAGGCATGAAAT<br>R: GGCATTGGAAACCTGATCTT<br>F: GGGACGAGAAGAACAAGCAG<br>R: CGCGATATCCAGTACACCAA<br>F: AATCAGGAAGACACCAACACG<br>R: CTCCAATTGACGAAGCAAAT<br>F: CCACTTACCCCGCAGTTCTA<br>R: GTAGCTCGTTCTCTGGCCTCT
***Myo-sex* RACE**	5’:CTTAGCAACTCGATCTCCTTCATCCGTT<br>5’:TCTTTGGAGGCCTTGTCCTGTTCAA<br>3’:AAGTGCGGGTTAGATGAAGCAGAAACA<br>3’:GAACGTTTGGCACAAGAGGAGGATG
**BAC NDL62N22**	F: TGCATGAATCTGCTTGGGTA<br>R: AGGCGTGTAGACACGAAGGT<br>F: CAGTGGTTGGAAAGCACGTA<br>R: ATTTCCGAAGTGTGGAGCTG
