# Supplementary material for: Insights into the Preservation of the Homomorphic Sex-Determining Chromosome of Aedes aegypti from the Discovery of a Male-Biased Gene Tightly Linked to the M-Locus
Source: Genome Biol Evol. 2014 Jan 6;6(1):179–91. doi: 10.1093/gbe/evu002 (PMC3914700; doi:10.1093/gbe/evu002)
Supplement: Supplementary Data [file supp_evu002_supplemental-table-S2-RNA-seq_table.pdf]

Supplemental Table S2: RNA-seq alignments to *myo-sex*

Total RNA-seq Reads

0-2 hr. embryo	2-4 hr. embryo	4-8 hr. embryo	8-12 hr. embryo	Larva	Pupa	Adult Male	Adult Female
6,629,888	8,172,615	7,989,804	12,681,907	12,011,730	14,584,158	12,691,887	25,101,990

RNA-seq alignments to *myo-sex* with 100 percent nucleotide identity

0-2 hr. embryo	2-4 hr. embryo	4-8 hr. embryo	8-12 hr. embryo	Larva	Pupa	Adult Male	Adult Female
0	0	4	3	553	67,627	3,641	0
