# Supplementary material for: Insights into the Preservation of the Homomorphic Sex-Determining Chromosome of Aedes aegypti from the Discovery of a Male-Biased Gene Tightly Linked to the M-Locus
Source: Genome Biol Evol. 2014 Jan 6;6(1):179–91. doi: 10.1093/gbe/evu002 (PMC3914700; doi:10.1093/gbe/evu002)
Supplement: Supplementary Data [file supp_evu002_Supplemental_FigureS1_additionalDataOn_myo-sex_expression.pdf]

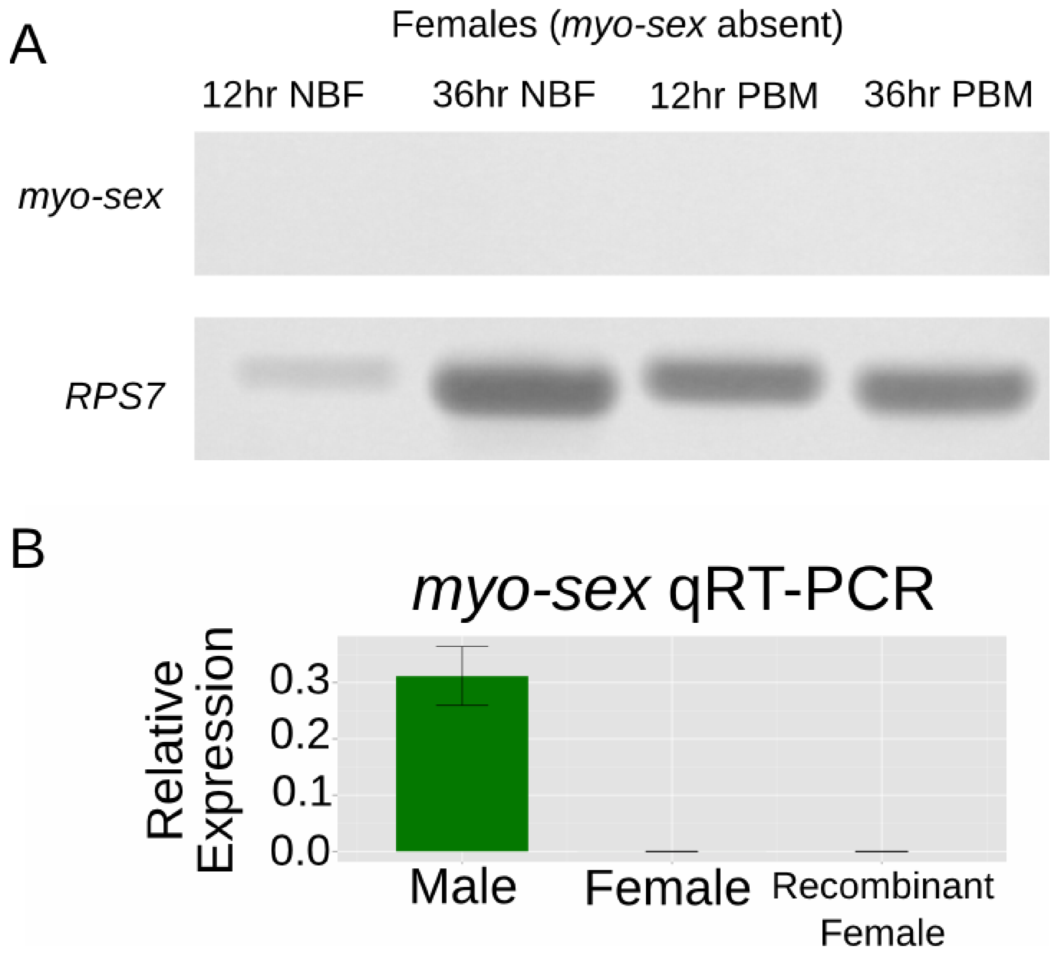

Supplemental Figure S1. A) *Myo-sex* is not expressed in wildtype adult females. *Myo-sex* is not normally present in females, and is therefore not normally expressed in females. RT-PCR performed on virgin and blood-fed female Liverpool strain *A. aegypti* cDNA. Bands were present in all four samples for the RPS7 positive control. No bands were visible from the four samples after 32 cycles of amplification using *myo-sex* primers. B) Quantitative RT-PCR performed on males, non-recombinant females, and the $m/m^{J2sensor}$ recombinant females. Data are from three biological replicates of pools of three individuals for each test group. Relative expression levels were calculated using the ΔCt method with RPS7 as the endogenous control.
